# Disruptive Selection of Human Immunostimulatory and Immunosuppressive Genes Both Provokes and Prevents Rheumatoid Arthritis, Respectively, as a Self-Domestication Syndrome

**DOI:** 10.3389/fgene.2021.610774

**Published:** 2021-06-22

**Authors:** Natalya V. Klimova, Evgeniya Oshchepkova, Irina Chadaeva, Ekaterina Sharypova, Petr Ponomarenko, Irina Drachkova, Dmitry Rasskazov, Dmitry Oshchepkov, Mikhail Ponomarenko, Ludmila Savinkova, Nikolay A. Kolchanov, Vladimir Kozlov

**Affiliations:** ^1^Institute of Cytology and Genetics, Siberian Branch of the Russian Academy of Sciences (ICG SB RAS), Novosibirsk, Russia; ^2^Research Institute of Fundamental and Clinical Immunology (RIFCI SB RAS), Novosibirsk, Russia

**Keywords:** rheumatoid arthritis, human, gene, promoter, TATA box, candidate SNP marker, self-domestication syndrome, RNA-**seq** verification

## Abstract

Using our previously published Web service SNP_TATA_Comparator, we conducted a genome-wide study of single-nucleotide polymorphisms (SNPs) within core promoters of 68 human rheumatoid arthritis (RA)-related genes. Using 603 SNPs within 25 genes clinically associated with RA-comorbid disorders, we predicted 84 and 70 candidate SNP markers for overexpression and underexpression of these genes, respectively, among which 58 and 96 candidate SNP markers, respectively, can relieve and worsen RA as if there is a neutral drift toward susceptibility to RA. Similarly, we predicted natural selection toward susceptibility to RA for 8 immunostimulatory genes (e.g., *IL9R*) and 10 genes most often associated with RA (e.g., *NPY*). On the contrary, using 25 immunosuppressive genes, we predicted 70 and 109 candidate SNP markers aggravating and relieving RA, respectively (e.g., *IL1R2* and *TGFB2*), suggesting that natural selection can simultaneously additionally yield resistance to RA. We concluded that disruptive natural selection of human immunostimulatory and immunosuppressive genes is concurrently elevating and reducing the risk of RA, respectively. So, we hypothesize that RA in human could be a self-domestication syndrome referring to evolution patterns in domestic animals. We tested this hypothesis by means of public RNA-Seq data on 1740 differentially expressed genes (DEGs) of pets vs. wild animals (e.g., dogs vs. wolves). The number of DEGs in the domestic animals corresponding to worsened RA condition in humans was significantly larger than that in the related wild animals (10 vs. 3). Moreover, much less DEGs in the domestic animals were accordant to relieved RA condition in humans than those in the wild animals (1 vs. 8 genes). This indicates that the anthropogenic environment, in contrast to a natural one, affects gene expression across the whole genome (e.g., immunostimulatory and immunosuppressive genes) in a manner that likely contributes to RA. The difference in gene numbers is statistically significant as confirmed by binomial distribution (*p* < 0.01), Pearson’s χ^2^ (*p* < 0.01), and Fisher’s exact test (*p* < 0.05). This allows us to propose RA as a candidate symptom within a self-domestication syndrome. Such syndrome might be considered as a human’s payment with health for the benefits received during evolution.

## Introduction

Rheumatoid arthritis (RA) is an autoimmune disease involving autoantibodies (e.g., anti-citrullinated protein antibodies) and proinflammatory cytokines (e.g., TNF-α and IL-6) that participate in the induction of chronic synovitis and bone erosion, followed by deformity (Smolen and Aletaha, 2015), which are some of the most prevalent causes of disability (Koller and Nobauer-Huhmann, 2009). Currently, it is widely accepted that RA immunopathogenesis is mostly mediated by the mechanisms involving a breakdown of immune tolerance to self antigens that is characterized by an increase in the activity of effector T cells causing RA symptoms (Sarkander et al., 2016). Two subpopulations of helper cells, Th1 and Th17, are mostly responsible for the increase in the activity of effector T cells (Frey et al., 2010). Additionally, memory cells of adaptive immunity in a given individual keep information on all the diseases survived; these cells will increase resistance to these diseases in the future (Sarkander et al., 2016). On the contrary, low activity of regulatory cells [e.g., regulatory T cells (Tregs) and myeloid suppressor cells] is often seen in RA (Alsaed et al., 2018). The immunosuppressive-activity deficit is one of the central features of RA pathogenesis (Lawson et al., 2006). Moreover, in patients with RA, an increase in the resistance of effector T cells to the suppressive action of Tregs is detectable too (Malemud, 2018). There are numerous factors related to disturbances in the mechanisms regulating immunocompetent cells, e.g., primarily, cytokines, and their receptors as well as transcription factors.

Rheumatoid arthritis cannot be completely cured (Smolen et al., 2016) in terms of autoimmunity because memory immune cells in the blood are able to retain information about antigens for a long time after successful treatment (Sarkander et al., 2016). For this reason, clinicians usually talk only about RA remission (Smolen and Aletaha, 2015). Because the immune system is more potent in women, their risk of RA is three times higher than that of men (Krasselt and Baerwald, 2017) and increases with the level of sex hormones after menopause and during pregnancy (Ho and Weinshilboum, 2017) but decreases during lactation (Karlson et al., 2004). A retrospective clinical and pharmacological meta-analysis of 29,880 RA patients in comparison with 73,758 relatively healthy volunteers identified 42 loci and 98 genes as candidate therapeutic targets in RA within the framework of predictive, preventive personalized, and participatory (4P) medicine (Okada et al., 2014).

Conventionally, RA risk is dependent on genetic factors and the lifestyle approximately equally (Nair et al., 2017), namely, the microbiome (reflecting a diet) (Sato et al., 2017), previous illnesses (Scott et al., 2010), environmental pollution (Klareskog et al., 2006), and bad habits (Malm et al., 2016) such as smoking (Erlandsson et al., 2016), physical inactivity, and overeating (Somers et al., 2014). That is why RA fits well the main idea of post-genomic 4P medicine, thus giving a chance to people to reduce their disease risks by correction of the lifestyle in line with their individual sequenced genomes (Trovato, 2014). This is important because at late RA stages, fibroblast-like synoviocytes are capable of hyperproliferation, which can cause leukemia in children with leukopenia (Jones et al., 2006) and, in adulthood, may lead to synovial hyperplasia as an impairment of the shape and mobility of joints (Lim and Bae, 2011).

The keystone of 4P medicine is the top scientific project of the 21st century, “1000 Genomes” (Telenti et al., 2016), due to which hundreds of thousands of individual human genomes are sequenced referenced in the variome data. This Big Data set contains consensus human genome sequences and hundreds of millions of single-nucleotide polymorphisms (SNPs) publicly available within databases Ensembl (Zerbino et al., 2015), the UCSC Genome Browser (Haeussler et al., 2015), and dbSNP (Day, 2010). Additionally, databases ClinVar (Landrum et al., 2014) and OMIM (Amberger et al., 2015) document, systematize, and prioritize only clinically proven and experimentally studied human disease SNP markers, respectively, whose allele frequencies significantly differ between cohorts of patients and conventionally healthy volunteers as a mandatory criterion (Varzari et al., 2019). Finally, the dbWGFP database (Wu et al., 2016) does the same in whole-genome mode for all the 10 billion potential SNPs in humans. If we assume that each SNP can affect at least 1 of known human 55,000 diseases (Pocai, 2019), such genetic load will be too high to survive in evolution. So, both Kimura’s theory (1968) and Haldane’s dilemma (1957) lead to the conclusion about neutrality of the vast majority of human SNPs. These neutral SNPs might be bioinformatically identified and discarded without time-consuming clinical testing. Although the current accuracy of bioinformatic calculations is not above the threshold of clinical applicability yet (Yoo et al., 2015), this accuracy grows each year (Putlyaeva et al., 2018; Zorlu et al., 2019).

Most of the SNPs documented in the OMIM database (Amberger et al., 2015) are within protein-coding regions of human genes and correspond to aberrations in protein structure and therefore function (Mitsuyasu et al., 1998). Indeed, these damages are uniform within any tissue and thus are easily detectable but cannot be corrected either therapeutically or *via* lifestyle changes. In contrast, SNPs within regulatory gene regions (Deplancke et al., 2016) have pathogenic manifestations correctable both by medication and by lifestyle changes within the framework of 4P medicine because these manifestations are limited to alterations of gene expression levels without any protein damage; the latter is negligibly rare among experimentally studied regulatory SNPs (Amberger et al., 2015). Certainly, many factors can independently modulate expression levels of the majority of genes; this situation complicates interpreting the expression patterns of these genes as partial contributions of such modulators as SNPs, somatic mutations, stressors, silencers, inhibitors, and activators. Actually, exogenous recombinant activated coagulation factor VII (*F7*), as an adjunctive therapy, can successfully help to urgently stop internal bleeding caused by acquired hemophilia as an autoimmune complication of RA, which is treated with immunosuppressive therapy at the same time (Drobiecki et al., 2013). The best-studied regulatory SNPs are mostly within 70-bp regions upstream of transcription start sites (Bhuiyan and Timmers, 2019) and affect gene expression levels proportionally with effect of these SNPs on the binding affinity of TATA-binding protein (TBP) for TBP-sites in these promoter regions (Mogno et al., 2010) when TATA box is canonical (Ponomarenko et al., 2013). According to the EPD database (Dreos et al., 2017), only ∼15% of eukaryotic gene promoters contain canonical TATA boxes as TBP-sites (Bucher, 1990), whereas genome-wide chromatin immunoprecipitation experiments (ChIP-seq) have detected such sites upstream of all the transcription start sites within eukaryotic genomes (Rhee and Pugh, 2012). Indeed, the binding of TBP to TBP-sites of genes shifts the equilibrium from transcriptionally inactive packing of these genes to pre-initiation complexes necessary to initiate the expression of these genes (Godde et al., 1995) as proven in TBP knockout mice (Martianov et al., 2002).

We developed our Web service SNP_TATA_Comparator^1^ (Ponomarenko et al., 2015), whose input consists of two promoter DNA sequences representing ancestral and minor alleles of the SNP being examined; the software generates TBP-binding affinity estimates for these promoter alleles (± standard error) and significance α of their difference with Fisher’s *Z*-test (Waardenberg et al., 2015). We applied it from SNP to SNP to predict their contribution to diseases [e.g., chronopathologies (Ponomarenko et al., 2016)] and selectively verified the obtained results using F1-hybrid mice (Chadaeva I. et al., 2019), real-time polymerase chain reaction (Oshchepkov et al., 2019), RNA-Seq data (Vasiliev et al., 2021), gel retardation assay, stopped-flow spectrometry, biosensors, or bioluminescence, as reviewed (Ponomarenko et al., 2017). SNP_TATA_Comparator (Ponomarenko et al., 2015) is already used in independent clinical studies [e.g., in a pulmonary tuberculosis case-control study (Varzari et al., 2018)]. In the present work, at the stage of comprehensive experimental validation, we tested whole-genome sequence-based predictions for RA-associated candidate biomedical SNP markers *in vivo* using publicly available RNA-Seq data (Albert et al., 2012; Hekman et al., 2018; Yang et al., 2018). Finally, we discuss the results of the verification of the SNP_TATA_Comparator predictions vis-à-vis the semiquantitative RNA-Seq data for the next step: further comprehensive experimental verification of our biomedical predictions using SNP_TATA_Comparator compared with genome-wide data on quantitative trait loci, QTLs [e.g., in human cardiopathology (Koopmann et al., 2014)].

## Materials and Methods

### DNA Sequences

We retrieved 1896 SNPs of 68 human genes from the dbSNP database, build No. 151, and DNA sequences from Ensembl (reference genome assembly GRCh38/hg38) using the UCSC Genome Browser.

### Analysis of DNA Sequences

**FIGURE 1:**
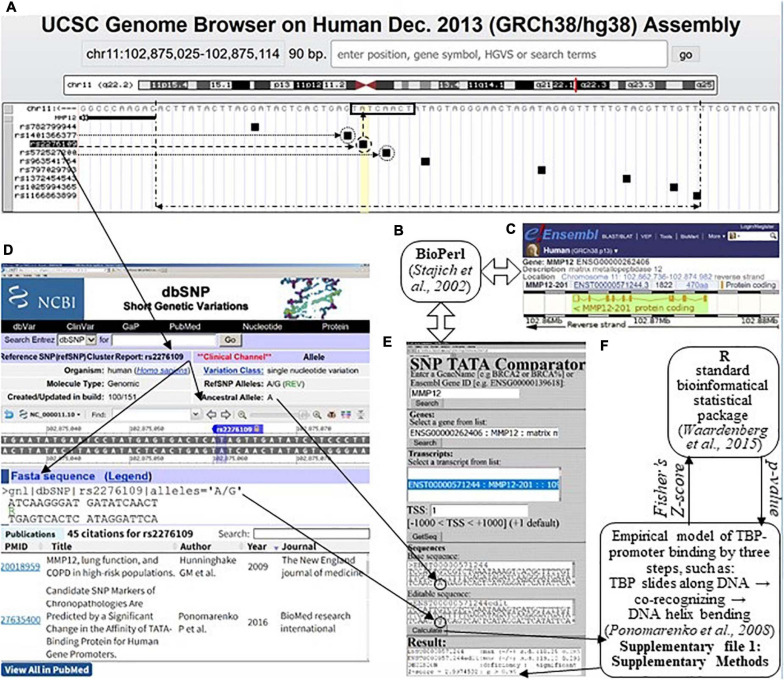
The result produced by SNP_TATA_Comparator (Ponomarenko et al., 2015) in the case of a clinically proven SNP marker (rs2276109) of an asthma risk reduction due to MMP12 downregulation (Hunninghake et al., 2009), which is associated with a reduced risk of rheumatoid arthritis, RA (Liu et al., 2004). **(A)** The UCSC Genome Browser (Haeussler et al., 2015) visualizes a 70-bp promoter (double-headed dash-and-dot arrow) of a given human gene (here: MMP12, row 2, left column), where there are a TBP-binding site (TBP-site; framed, □) and SNPs retrieved from dbSNP (Day, 2010); their IDs constitute the left-hand column. **(B)** The Bioperl toolkit (Stajich et al., 2002). **(C)** The Ensembl database (Zerbino et al., 2015). **(D)** The current build (No. 151) of the dbSNP database describes the SNP under study (rs2276109 in this example). **(E)** Our prediction of MMP12 downregulation (textbox “Result”: row 3) caused by SNP rs2276109 (circled) by means of its both alleles (i.e., ancestral and minor ones as input data within two textboxes “Basic sequence” and “Editable sequence,” respectively). Double-headed open arrows (⇔) depict how SNP_TATA_Comparator retrieved the ancestral DNA sequence of the human *MMP12* promoter from the Ensembl database (Zerbino et al., 2015) using the Bioperl toolkit (Stajich et al., 2002). **(F)** Our bioinformatics model of the TBP-promoter binding *via* three steps, as follows: (1) TBP sliding along DNA ↔ (2) TBP retarded by the TBP-site ↔ the TBP-promoter complex is fixed due to the bending of the DNA double helix at right angles (Ponomarenko et al., 2008). This model is based on the standard bioinformatical package of R (Waardenberg et al., 2015), as described in Supplementary File 1: Supplementary Methods. Solid arrows: data flows, when one predicts the manifestation of a given SNP using SNP_TATA_Comparator; dashed arrow: the SNP in this example (rs2276109); dotted arrows: two more SNPs, rs572527200 and rs1401366377 (Figure 3A), which correspond to either underexpression or overexpression of *MMP12*, respectively, as predicted in this work.

We used the web tool SNP_TATA_Comparator (Ponomarenko et al., 2015), the input of which are two proximal promoter sequences carrying either an ancestral (wt) or a minor (min) allele of an SNP being analyzed, as shown within two textboxes, “Basic sequence” and “Editable sequence,” respectively (Figure 1E). The double-headed open arrows (⇔) between Figures 1B,C,E explain how SNP_TATA_Comparator uses the Bioperl toolkit (Stajich et al., 2002) to retrieve the ancestral variant of the DNA sequence of the human *MMP12* promoter from database Ensembl (Zerbino et al., 2015) in an automated mode. The solid arrows between Figures 1A,D,E show the input of a minor variant of this sequence into SNP_TATA_Comparator according to its description within the database dbSNP (Day, 2010) visualized using the UCSC Genome Browser (Haeussler et al., 2015).

Using the “Calculate” option, we first estimated two (−ln(*K*_*D*_) ± δ) pairwise value sets of the highest observed estimate of TBP-promoter affinity ± its standard error according to our three-step approximation of their complex formation on each of these sequences independently, as depicted in Figure 1F and described in depth in Supplementary File 1 entitled Section “Supplementary DNA Sequence Analysis.” To this end, we took into account non-specific TBP-DNA affinity (Hahn et al., 1989), the position-weight matrix of TBP-sites (Bucher, 1990), minor-groove width of B-helical DNA (Karas et al., 1996), DNA melting during its bending, which fixes the TBP-promoter complex (Flatters and Lavery, 1998), and abundance levels of TA-rich dinucleotides (Ponomarenko et al., 1999) in the sequences analyzed. Then, we calculated Fisher’s *Z*-score and, finally, converted it into its *p*-value of statistical significance taken from standard software R (Waardenberg et al., 2015), as one can see in Figure 1F. Eventually, the “Result” textbox (Figure 1E) shows all the intermediate and final results, namely, −ln(*K*_*D*_^(wt)^) ± δ_(wt)_ and −ln(*K*_*D*_^(min)^) ± δ_(min)_ in lines 1 and 2, respectively; the prediction made using the terms “deficiency,” “excess,” “significant,” and “insignificant” in line 3 as proven experimentally (Mogno et al., 2010); and the *Z*-score and *p*-value in line 4.

Thus, we examined the SNPs one by one independently from the others and, as a result, either discarded those with insignificant effects on the TBP-promoter binding affinity according to our predictions (data not shown) or presented the SNPs in Supplementary Tables 1–4 (hereinafter: see Supplementary File 2 section “Supplementary Results”).

### Keyword Searches in the PubMed Database

For each SNP that can statistically significantly alter the expression of the studied human genes according to our prediction above, we handmade a standard keyword search in the PubMed database (Lu, 2011) as depicted in Supplementary Figure 1 (hereinafter: see Supplementary File 3 entitled section “Supplementary Keyword Search”).

### *In vivo* Validation of Our Current Predictions Using Public RNA-Seq Data

**FIGURE 2:**
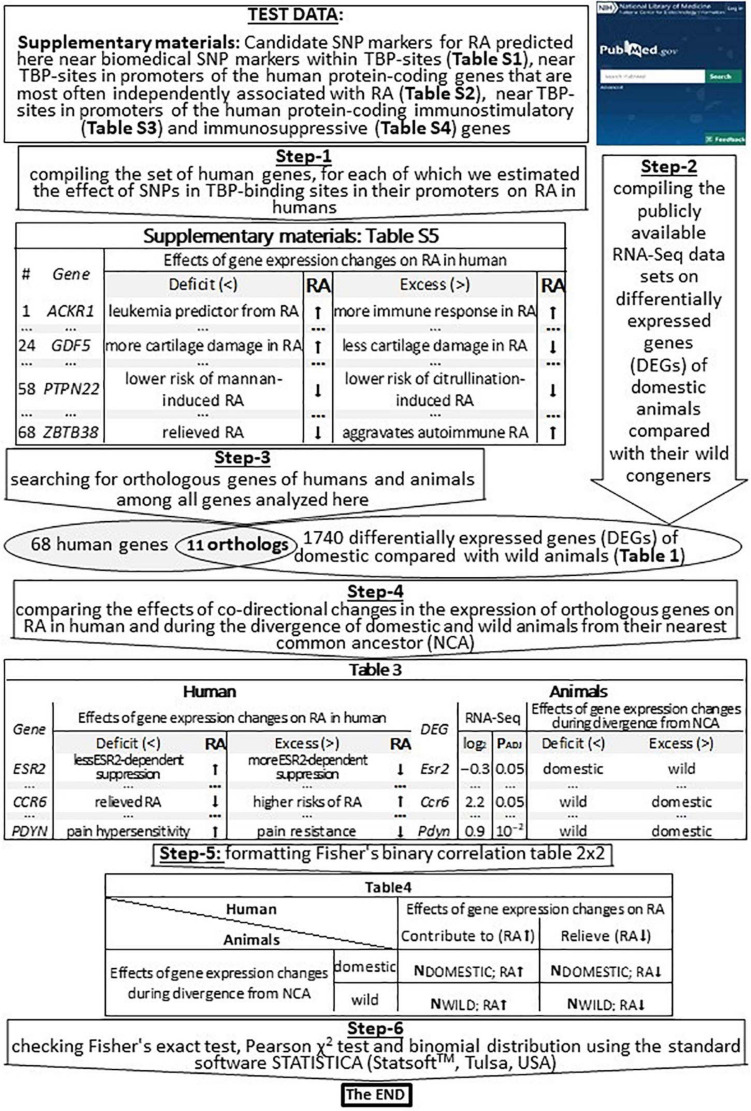
An algorithmic flowchart of *in vivo* validation of our current predictions by means of publicly available RNA-Seq data on differentially expressed genes (DEGs) of domestic vs. wild animals. RA, rheumatoid arthritis; effect on RA, contributes to (↑), relieves (↓); NCA, nearest common ancestor; *P*_*ADJ*_, significance [Fisher’s *Z*-test with those corrections on multiple comparisons that have been published by Albert et al. (2012), Hekman et al. (2018), and Yang et al. (2018)].

For *in vivo* validation of our predictions made in this work (Supplementary Tables 1–4) using public RNA-Seq data, we compiled all the 68 human genes whose effects on RA in humans have been estimated here, as shown in Figure 2 (Step-1), and we present them in Supplementary Table 5 (hereinafter, see Supplementary File 2 entitled section “Supplementary Results”).

We verified our computer-based prediction (that RA can be a self-domestication syndrome) by means of previously published publicly available whole-genome RNA-Seq data, as described in Table 1 and depicted in Figure 2 (Step-2). For minimizing the effects of false–positive errors, we selected only the differentially expressed genes (DEGs) that were statistically significant according to Fisher’s *Z*-test, with those corrections on multiple comparisons (*P*_*ADJ*_ < 0.05) that have been published by their authors (Albert et al., 2012; Hekman et al., 2018; Yang et al., 2018). Therefore, these data included DEGs in the frontal cortex of guinea pigs (*Cavia porcellus*) vs. cavies (*C. aperea*; 883 DEGs), pigs vs. boars (*Sus scrofa*; 30 DEGs), domesticated vs. wild rabbits (*Oryctolagus cuniculus*; 17 DEGs), tame vs. aggressive rats (*Rattus norvegicus*; 20 DEGs), and dogs (*Canis familiaris*) vs. wolves (*C. lupus*; 13 DEGs); these data were retrieved from another study (Albert et al., 2012). Additionally, we used 450 DEGs in the blood of dogs vs. wolves retrieved from Yang et al. (2018). Besides, we analyzed 327 DEGs within anterior pituitary tissues of adult male foxes (*Vulpes vulpes*) of two unique outbred lines artificially selected for domestication (tameness) or aggressiveness (Hekman et al., 2018). Thus, the total number of DEGs analyzed in this study was 1740.

**TABLE 1:** The investigated genome-wide RNA-Seq transcriptomes of domestic animals with their wild congeners publicly available in database PubMed.

No.	Domestic animals	Wild animals	Number of DEGs	Tissue	References
1	Guinea pigs (*Cavia porcellus*): three females and three males	Cavy (*C. aperea*): three females and three males	883	Frontal cortex	Albert et al., 2012
2	Pigs (*Sus scrofa*): five females	Boars (*S. scrofa*): five females	30		
3	Domesticated rabbits (*Oryctolagus cuniculus domesticus*): three females and 3 males	Wild rabbits (*Oryctolagus cuniculus*): three females and three males	17		
4	Tame rats (*Rattus norvegicus*): three females and three males	Aggressive rats (*R. norvegicus*): three females and three males	20		
5	Dogs (*C. familiaris*): three females and three males	Wolves (*C. lupus*): three females and one male	13		
6	Dogs (*Canis familiaris*): one female and one male	Wolves (*C. lupus*): two females and one male	450	Blood	Yang et al., 2018
7	Tame foxes (*Vulpes vulpes*): six males	Aggressive foxes (*V. vulpes*): six males	327	Pituitary	Hekman et al., 2018
Total	Six domestic animal species: 17 females and 19 males	Six wild animal species: 18 females and 17 males	1740	3 tissues	

For the RNA-Seq data analysis, we employed the conventional concept of “divergence from the nearest common ancestor” (Samet, 1985), which we applied to domestic and wild animals whose DEGs were compared with orthologous human genes studied here, as suggested recently (Vasiliev et al., 2021). This procedure yielded 11 pairs of orthologous genes in humans and animals, as shown in Figure 2 (Step-3 and a Venn diagram). In this context, we compared the effects of codirected changes in the expression of orthologous genes on RA in humans and during the divergence of domestic and wild animals from their NEAREST COMMON ANCESTOR, as depicted in Figure 2 (Step-4). Accordingly, we filled out Fisher’s table 2 × 2 (Figure 2: Step-5) and, finally, tested this binary correlation table for statistical significance using standard package STATISTICA (StatSoft^TM^, Tulsa, OK, United States), as depicted in Figure 2 (Step-6).

### Statistical Analysis

Using options “Statistics” → “Non-parametrics” → “2 × 2 Table” in STATISTICA (StatSoft, Tulsa, OK, United States), we verified our predictions (about the candidate SNP markers of worse RA as a self-domestication syndrome) with the 1740 DEGs of domestic vs. wild animals (Table 1) by three criteria, namely, binomial distribution, Pearson’s χ^2^, and Fisher’s exact test.

## Results and Discussion

To test our Web service SNP_TATA_Comparator for robustness vis-à-vis the annual increase in the SNP count, we compared its predictions between two cases, namely, (1) a previous build (No. 147) of the dbSNP database dated 2016, and after that, (2) its current build (No. 151) dated 2017. As one can see in rows 3 and 4 of Table 2, during the period considered, the progress of biomedicine yielded a 1.5-fold increase (from 18 to 27) in the number of human genes with biomedical SNP markers in known TBP-sites and an almost threefold increase (from 225 to 646) in the number of SNPs within the gene regions in question. That is why here we first analyzed SNPs within only the human genes that were analyzed in our previous study on RA (Chadaeva I. V. et al., 2019), to compare the results obtained using the current build (No. 151) of database dbSNP (Day, 2010) with those obtained by means of the previous build (No. 147) of this database. Below, we present the results on some genes.

**TABLE 2:** Candidate SNP markers of rheumatoid arthritis (RA) that are located near TBP-sites of human gene promoters as predicted in this work, in comparison with genome-wide patterns.

Data: GRCh38 (Zerbino et al., 2015) dbSNP build 151 (Day, 2010)				Result	Neutral drift (Haldane, 1957; Kimura, 1968; Kasowski et al., 2010)			H_0_: ↑↓-equivalence		
		
No.	SNPs	N_*G*_	N_*S*_	N_*R*_	N_>_	N_<_	*p*(H_0_: N_>_ < N_<_)	N_↑_	N_↓_	*p*(H_0_: N_↑_≡N_↓_)
1	Whole-genome norm for SNPs of TBP-sites (The 1000 Genomes Project Consortium (GPC) et al., 2012)	10^4^	10^5^	10^3^	200	800	>0.99	–	–	–
2	Clinical SNP markers of diseases at TBP-sites (Ponomarenko et al., 2015)	33	203	51	14	37	>0.99	–	–	–
3	Candidate SNP markers of RA near clinical SNP markers of diseases at TBP-sites (Chadaeva I. V. et al., 2019)	18	225	42	12	30	>0.99	32	10	<10^–4^
4	Candidate SNP markers of RA near clinical SNP markers of diseases at TBP-sites (this work)	25	603	154	84	70	>0.15	96	58	<10^–2^
5	Candidate SNP markers of RA near TBP-sites of the genes most often associated with RA (this work)	10	466	69	46	23	<10^–2^	42	27	<0.05
6	Candidate SNP markers of RA near TBP-sites of immunostimulatory genes (this work)	8	479	114	71	43	<0.01	71	43	<10^–2^
7	Candidate SNP markers of RA near TBP-sites of immunosuppressive genes (this work)	25	928	179	104	75	<0.025	70	109	<10^–2^
8	**Total**	**68**	**1896**	**516**	–	–	–	–	–	–

*Rheumatoid arthritis (RA): contribute to (↑) and relieve (↓). N_*G*_ and N_*S*_: total numbers of the human genes and of their SNPs meeting the criteria of this study. N_*R*_: the total number of the candidate SNP markers increasing (N_>_) or decreasing (N_<_) the affinity of TATA-binding protein (TBP) for these promoters studied in this work. N_]↑_ and N_↓_: total numbers of the candidate SNP markers that can contribute to or relieve RA, respectively. P(H_0_): the estimate of probability for the acceptance of this H_0_ hypothesis, according to a binomial distribution. TBP-site: TBP-binding site.*

### Candidate SNP Markers of RA Near Clinical SNP Markers of Diseases at TBP-Sites

The human *MMP12* gene encodes macrophage elastase and carries an SNP, rs2276109. Rs2276109 is a clinically proven SNP marker of an asthma risk reduction due to MMP12 deficiency (Hunninghake et al., 2009), as one can see in Figures 1A,D. Figure 1E presents how, using SNP_TATA_Comparator (Ponomarenko et al., 2015), we predicted that this SNP causes MMP12 deficiency (i.e., the “Decision” line of the “Result” textbox) because this SNP damages the TBP-site within the promoter of this gene, as shown by the dashed arrows in Figure 1A. This match between our prediction and the clinical data in question (Hunninghake et al., 2009) indicates suitability of SNP_TATA_Comparator for biomedical studies *in silico*, as highlighted in bold in the first row of Supplementary Table 1. In the two rightmost columns of this table, just below the citation of these clinical data, readers can see other data on *MMP12* downregulation as a clinically proven physiological marker of RA (Liu et al., 2004); we found these data by means of keywords in the PubMed database. This finding allows us to predict that the clinical SNP marker (rs2276109) of the reduced risk of asthma is a candidate SNP marker of a reduced risk of RA too, as we highlighted in *italics* in Supplementary Table 1.

**FIGURE 3:**
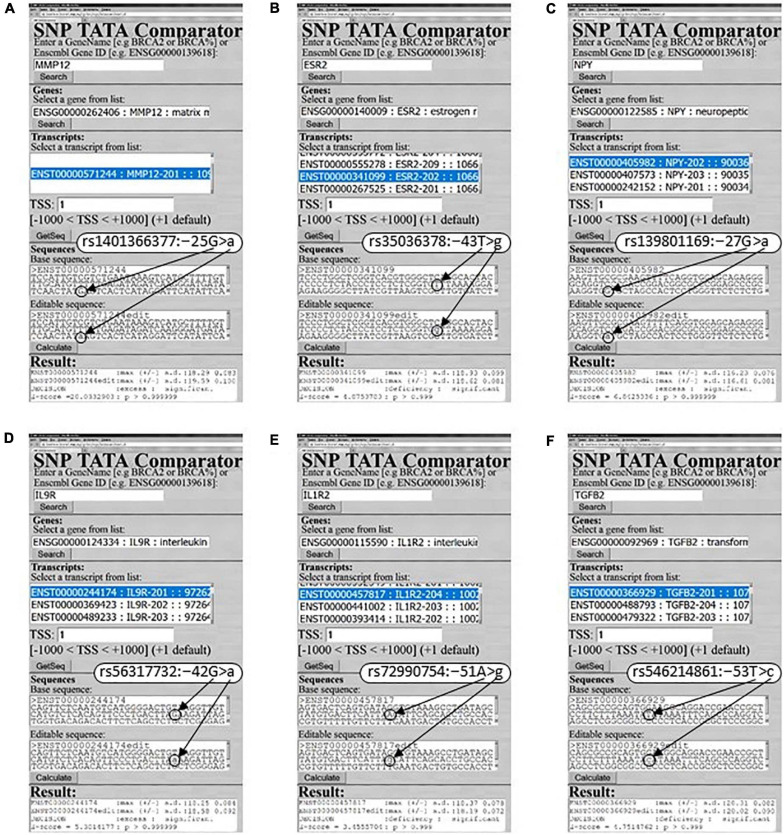
Examples of our predictions for human RA-related genes in this work. **(A)** *MMP12*: rs1401366377; **(B)** *ESR2*: rs35036378; **(C)** *NPY*: rs139801169; **(D)** *IL9R*: rs56317732; **(E)** *IL1R2*: rs72990754; **(F)** *TGFB2*: rs546214861.

As one can see in Figure 1A, there are eight more SNPs within the analyzed 70-bp proximal promoter region depicted by a double-headed dash-and-dot arrow; two of them can either decrease (rs572527200) or increase (rs1401366377) *MMP12* expression (dotted arrows and circles) according to our predictions, as exemplified by Figure 3A. With this in mind, on the basis of the same clinical data (Liu et al., 2004), we predicted two more candidate SNP markers of either decreased (rs572527200) or increased (rs1401366377) risk of RA, as shown in Supplementary Table 1.

Likewise, we one-by-one updated the calculation results for those 13 human genes (*ACKR1*, *APOA1*, *DHFR*, *F3*, *F7*, *HBB*, *HBD*, *IL1B*, *INS*, *MBL2*, *NOS2*, *SOD1*, and *TPI1*) that we have already examined earlier and described in detail in our previous article on RA (Chadaeva I. V. et al., 2019); the updated results on these genes are found in Supplementary Table 1. Finally, after the publication of our previous article on RA (Chadaeva I. V. et al., 2019), within PubMed (Lu, 2011), we found clinically proven SNP disease markers located within 70-bp proximal promoters of the 11 human genes (*ADH7*, *CETP*, *COMT*, *ESR2*, *FGFR2*, *HSD17B1*, *HTR2C*, *MLH1*, *PDYN*, *RET*, and *TGFBR2*), as described below for the first time.

Human gene *PDYN* (i.e., prodynorphin as a basic building block of endorphins inhibiting pain and causing euphoria as hormones/neurotransmitters of joy) has a known SNP marker (rs886056538) of spinocerebellar ataxia when PDYN is in excess (Supplementary Table 1) according to the ClinVar database. Our keyword search in PubMed pointed to a rat model of human diseases, where the pain sensitivity threshold in RA positively correlates with this hormone’s abundance (Zheng et al., 2014). With this in mind, we predicted that rs886056538 is a candidate SNP marker of relieved RA as presented in Supplementary Table 1. As readers can see in this table, in the vicinity of rs886056538, we selected nine SNPs (e.g., rs1195765727), each of which can reduce the PDYN level and thus may be a candidate SNP marker of relieved RA too (Supplementary Table 1).

The human *COMT* gene encoding catechol-O-methyltransferase bears two SNP markers, rs370819229 and rs777650793, implicated by the ClinVar database in dilated cardiomyopathy and other cardiovascular diseases, respectively, because of COMT downregulation and upregulation calculated in our study (Supplementary Table 1). Within PubMed, we found a short clinical communication (Finan and Zautra, 2013) on a negative correlation between chronic pain sensitivity in RA and COMT. In accordance with these clinical observations, we proposed two known SNP markers of cardiovascular pathologies (rs370819229 and rs777650793) as candidate SNP markers of worsened and alleviated RA, respectively, as shown in Supplementary Table 1. In total, nearby we predicted two more candidate SNP markers (rs901020754 and rs779542396) worsening RA and 13 more candidate SNP markers relieving RA (e.g., rs748298389), all of which are given in Supplementary Table 1.

Human gene *RET* codes for the Ret proto-oncogene, whereas database ClinVar shows two SNPs, rs10900297 and rs10900296, located within its 70-bp proximal promoter, because they occur in patients with either pheochromocytoma or renal dysplasia (Supplementary Table 1). According to our predictions (Supplementary Table 1), these SNPs can surprisingly cause over- and under-expression of this gene, respectively, as confirmed by two clinical studies (Bridgewater et al., 2008; Sarin et al., 2014) retrieved by our keyword search in PubMed. Moreover, in this way, we found a clinical case report (Townsend et al., 1994) where both pheochromocytoma and RA were mutually complicating diagnoses. For this reason, we suggest two candidate SNP markers (rs10900297 and rs10900296) of complications in RA diagnosis and the same for four other SNPs, which can either significantly reduce the RET level (i.e., rs551321384 and rs1191017949) or significantly elevate it (i.e., rs1237152255 and rs1372293149), as readers can see in Supplementary Table 1.

Human gene *MLH1* encodes DNA mismatch repair protein MLH1; in this gene’s promoter, ClinVar lists two SNP markers (rs63750527 and rs756099600) of colon cancer that elevate the MLH1 level, thus preventing cancer cell apoptosis during cancer chemotherapy and an immune response (Supplementary Table 1). We found a retrospective association (Jeong et al., 2017) (comorbidity) of colon cancer and RA. Accordingly, we predicted two candidate SNP markers (rs63750527 and rs756099600) of high risk of comorbidities in RA (Supplementary Table 1). Additionally, near rs63750527 and rs756099600, we identified three more SNPs also increasing both the MLH1 amount and colon cancer risk (e.g., rs752622244) and hence elevating the risk of an RA comorbidity (Supplementary Table 1). In the table, the reader can see three other SNPs that are located within the *MLH1* promoter (e.g., rs864622145) and reduce an expression of this gene, thereby possibly accelerating the progression of RA to cancer because of reduced DNA repair (Kullmann et al., 2000).

Human gene *ADH7* encoding alcohol dehydrogenase 7 carries a biomedically identified SNP marker (rs17537595) of esophageal cancer; the minor allele of this SNP can reduce *ADH7* expression (Abbas et al., 2006) in agreement with our prediction presented in Supplementary Table 1. Our keyword search in PubMed revealed that both a deficit and an excess of this enzyme occur quite often in digestive-tract cancers (Jelski et al., 2008) that are comorbid with RA (Hemminki et al., 2012). That is why, for increased risk of comorbidities of RA, we predicted candidate SNP marker rs17537595 as well as three more markers nearby (rs372329931, rs755152695, and rs1238877951) having the same effect on this gene’s expression (Supplementary Table 1).

The promoter of human gene *HSD17B1* contains SNP rs201739205, which decreases the amount of hydroxysteroid (17-β) dehydrogenase 1 encoded by this gene (Supplementary Table 1), as revealed in patients with hereditary breast cancer (Peltoketo et al., 1994), which is discordant to RA (Chen et al., 2019) according to the results of our keyword search in database PubMed. Therefore, we predicted a candidate SNP marker (rs201739205) of low risk of RA (Supplementary Table 1). Additionally, around it, we chose two more SNPs (rs748743528 and rs779674159) that decrease the HSD17B1 level and therefore are candidate SNP markers of an RA risk reduction too (Supplementary Table 1). Finally, within the promoter under study, we predicted three SNPs able to cause HSD17B1 overexpression (e.g., rs1332869256), each of which can also cause breast cancer (He et al., 2016) according to our PubMed search and therefore may be a candidate SNP marker of decreased risk of RA (Supplementary Table 1).

The human *ESR2* gene coding for estrogen receptor 2 (β) carries a clinically proven SNP marker (rs35036378) of an ESR2-deficient primary pT1 tumor of the mammary gland (Philips et al., 2012), for which we also correctly predicted ESR2 underexpression, as shown in both Figure 3B and Supplementary Table 1. Our PubMed keyword search yielded a mouse model of human diseases with ESR2-dependent suppression of inflammation (Armstrong et al., 2013). These data allowed us to predict a candidate SNP marker (rs35036378) of reduced suppression of inflammation in RA (Supplementary Table 1). Nearby, we identified the only SNP (rs766797386) that can decrease the ESR2 level and consequently is a candidate SNP marker of reduced suppression of inflammation in RA (Supplementary Table 1).

The human *FGFR2* gene (fibroblast growth factor receptor 2) is annotated in the ClinVar database showing two biomedical SNP markers in its promoter: rs886046768 for craniosynostosis and rs387906677 for bent bone dysplasia, which correspond, respectively, to an excess and deficit of this receptor according to our predictions (Supplementary Table 1). As for RA, using our keyword search in PubMed, we found a physiological *in vitro* model of human angiogenesis, where neovascularization increases with an FGFR2 concentration increase and vice versa (Brown et al., 1996). This finding permits us to predict that all SNPs in this gene’s promoter, which can significantly elevate (e.g., rs886046768) and reduce (e.g., rs387906677) this receptor’s level, are candidate SNP markers of elevated and reduced neovascularization in RA, respectively (Supplementary Table 1).

The promoter of human gene *TGFBR2* has a known SNP marker (rs138010137) of aortic thoracic aneurysm according to database ClinVar. Our prediction for this SNP is underexpression of transforming growth factor beta receptor 2 encoded by this gene (Supplementary Table 1). We learned that a TGFBR2 deficit disrupts regulatory T-cell homeostasis in RA (Wang et al., 2018). This allows us to suggest a candidate SNP marker (rs138010137) of higher risk of RA as well as nearby, one more candidate SNP marker (rs1300366819) of the same RA parameter because of a decrease in the TGFBR2 level, according to our prediction (Supplementary Table 1). Finally, in the same way, we found out that synovial-fibroblast proliferation increases with an increase in the TGFBR2 level (Bira et al., 2005). Consequently, yet another SNP (rs1310294304) located within this promoter can be a candidate SNP marker of worsened RA because of TGFBR2 upregulation (Supplementary Table 1).

The human *CETP* gene codes for plasma lipid transfer protein and has a widely used SNP marker (rs1427119663) of hyperalphalipoproteinemia, which is an 18-bp deletion including the TBP-site in the promoter region of this gene; this mutation causes CETP underexpression (Plengpanich et al., 2011), as described in Supplementary Table 1. Because there is a known phenomenon of CETP-deficient mortality in RA (Ferraz-Amaro et al., 2013), in line with our keyword search in PubMed, we predicted rs1427119663 as a candidate SNP marker of complicated RA (Supplementary Table 1). In its vicinity, we identified five SNPs (e.g., rs17231520), all of which can increase this gene’s expression as a known risk factor of RA (Kim et al., 2016), hence our suggestion to regard them as candidate SNP markers of higher risk of RA (Supplementary Table 1).

Human gene *HTR2C* coding for serotonin receptor 2C is mentioned in ClinVar owing to its clinically documented SNP marker (rs3813929) of obesity as a complication of olanzapine-based antipsychotic treatment; this marker is associated with an HTR2C excess according to our calculations (Supplementary Table 1). As for RA, using our keyword search *via* PubMed, we revealed that HTR2C overexpression elevates the risk of RA with obstructive sleep apnea (Jagannathan et al., 2017) in contrast to HTR2C underexpression, which reduces adipogenesis and thus the obesity-related risk of RA (Priyadarshini et al., 2018). Keeping this in mind, we predicted that rs3813929 and five more SNPs nearby (e.g., rs886838672) are candidate SNP markers of the obesity-related complications in RA, as readers can see in Supplementary Table 1.

### Predictions Based Upon dbSNP: Build No. 151 Versus Build No. 147

Row 4 of Table 2 sums up the aforementioned predictions on the basis of dbSNP, build No. 151, about the 603 SNPs within 25 genes’ promoters containing biomedical SNP markers, namely, a total number of candidate SNP markers is 154, that is, ∼fourfold higher than 42 for build No. 147 (row 3). In the three rightmost columns, readers can see the statistically significant predominance (a greater number) of candidate SNP markers contributing to RA (i.e., 32 in row 3 and 96 in row 4) over candidate SNP markers preventing RA (i.e., 10 in row 3 and 58 in row 4). This result supports the robustness of our predictions in both compared cases. This predominance of predisposition over resistance to RA fits the notion that diversity of adaptive-immunity memory cells in an individual grows with an increase in the number of diseases patient survived, thus elevating the disease resistance in the future (Sarkander et al., 2016) and the risk of false activation of these cells according to Ashby’s law (Schuldberg, 2015).

Besides, the three central columns illustrate how we verified the robustness of our predictions with the annual growth of dbSNP from the standpoint of both Kimura’s theory (1968) and Haldane’s dilemma (1957), which highlight neutral drift of most SNPs as the cornerstone of the human genome as a whole. As a bioinformatics criterion of neutral drift within regulatory regions of the genome, some authors (Kasowski et al., 2010) first noted empirically and next proposed heuristically to estimate an excess in the number of SNPs damaging protein-binding sites over those improving them. In row 1, readers can see that the genome-wide pattern of SNPs in humans that was identified using ChIP-seq data (The 1000 Genomes Project Consortium (GPC) et al., 2012) fits this neutral drift criterion at a probability rate (*P*) of >0.99 and actually follows a binomial distribution, indeed. Row 2 presents the genome-wide pattern of the clinically proven biomedical SNP markers near the TBP-sites within human gene promoters according to our predictions made by means of SNP_TATA_Comparator, and this pattern also matches the same criterion of neutral drift. This finding reflects the essence of Haldane’s dilemma (1957), who hypothesized an under-threshold level of the human genetic load. In row 3, there are relevant results of our previous research (Chadaeva I. V. et al., 2019) on the candidate SNP markers of RA within a previous build of dbSNP No. 147; again, they seem to be in agreement with the neutral drift criterion used. As for the candidate SNP markers of RA predicted using build No. 151 of this database (row 4), contrary to all of the above, there is a lower, not higher, number of SNPs damaging TBP-sites than those improving them; this result is insignificant (*P* > 0.15) and does not allow to rule out their neutral drift completely. This indicates that almost a fourfold greater number of SNPs within the current build of dbSNP relative to the previous one may allow us to detect other genome-wide patterns of candidate SNP markers of RA besides their neutral drift, which is unquestionable (Haldane, 1957; Kimura, 1968). Therefore, we next examined the human genes that are most often associated with RA according to independent sources, as presented in Supplementary Table 2 and described below.

### Candidate SNP Markers of RA Near TBP-Sites in Promoters of the Human Protein-Coding Genes That Are Most Often Independently Associated With RA

The human *NPY* gene (neuropeptide Y) has four SNPs causing an excess of this protein (e.g., Figure 3C: rs139801169) and four SNPs diminishing its level (e.g., rs1223788416), which correspond to either higher or lower risk of obesity that is comorbid with RA (Stofkova et al., 2009), as found in PubMed. Thus, we predicted eight RA-related candidate SNP markers (Supplementary Table 2).

The human *CCR6* promoter contains two SNPs rs1433814180 and rs1047738754 that both can decrease the amount of C–C motif chemokine receptor 6 encoded by this gene, as shown in Supplementary Table 2 presenting our predictions. Our keyword search within the PubMed database revealed a CCR6-deficient mouse model of human RA with reduced autoimmunity (Bonelli et al., 2018). This result allows us to propose two candidate SNP markers of decreased risk of RA (Supplementary Table 2).

Human gene *CTLA4* (cytotoxic T-lymphocyte associated protein 4) carries four SNPs causing an overabundance this protein (e.g., rs127192924), which is a molecular biomarker of the overlapping autoimmunity proven by a case-control study (AlFadhli, 2013), as indicated in Supplementary Table 2. In addition, within its proximal promoters, there are four other SNPs whose minor alleles reduce the CTLA4 level according to our predictions (Supplementary Table 2: e.g., rs561368432), while there are CTLA4-inducible knockout mice as a laboratory animal model of human RA with an elevated autoimmune response (Alissafi et al., 2017). Considering all of the above, we predicted eight candidate SNP markers of worsened RA.

In total, within 10 human genes *CCR6*, *CTLA4*, *HLA-A*, *IL23R*, *IRF5*, *NPY*, *PADI4*, *PTPN22*, *STAT4*, and *TRAF1*, we predicted 42 and 27 candidate SNP markers, respectively, corresponding to a high and low risk of RA (Supplementary Table 2). On the other hand, 46 and 23 of all of them can, respectively, improve and damage TBP-sites within the promoters of the RA-related human genes examined, as shown in row 5 of Table 2. Thus, thanks to a fourfold increased number of SNPs within the current build No. 151 of dbSNP, besides the neutral drift toward predisposition to RA in humans (Table 2: rows 3 and 4), we first detected the natural selection against underexpression of the human genes often associated with RA; this selection could indeed elevate the risk of RA, as explained above. In hopes of further detailing this trend of natural selection, we next investigated human immunostimulatory and immunosuppressive genes independently from one another, as presented in Supplementary Tables 3, 4, respectively, and discussed below.

### Candidate SNP Markers of RA Near TBP-Sites in Promoters of Human Protein-Coding Immunostimulatory Genes

Human gene *IL9R* codes for interleukin 9 receptor and includes two SNPs rs56317732 and rs945044791 corresponding to its overexpression and underexpression, respectively, according to our predictions exemplified by Figure 3D as well as to high and low both inflammation and fibroblast-like synoviocyte proliferation in RA (Raychaudhuri et al., 2018). Therefore, we proposed them as candidate SNP markers for aggravated and alleviated RA (Supplementary Table 3).

Looking through Supplementary Table 3, readers can see a significant number of candidate SNP markers of improved TBP-sites than candidate SNP markers of damaged TBP-sites (71 vs. 43) within promoters of eight human immunostimulatory genes *ATF3*, *CCR7*, *IL3RA*, *IL9R*, *IL25*, *LCK*, *NFKB1*, and *ZBTB38* (*p* < 0.01, binomial distribution), as summarized in row 6 of Table 2. This corresponds to a significant predominance of 71 candidate SNP markers increasing RA-related risks over 43 such markers reducing these risks (*p* < 0.01), as presented *ibid*. Therefore, we can conclude that there is the pressure of natural selection on human immunostimulatory genes, and its direction both prevents their underexpression and elevates the human predisposition to RA, thus fitting the trends in the abovementioned rows 3, 4, and 5.

### Candidate SNP Markers of RA Near TBP-Sites in the Promoters of Human Protein-Coding Immunosuppressive Genes

Human *IL1R2* gene promoters contain two SNPs rs960068265 and rs946299576 able to increase the level of interleukin 2 receptor subunit β encoded by this gene as well as two other SNPs rs72990754 and rs960678696 capable of decreasing this level, as exemplified in Figure 3E. Using a keyword search in the PubMed database, we found a murine laboratory model of human diseases (Ocsko et al., 2018) where epigenetic silencing of this anti-inflammatory gene supported RA. Accordingly, Supplementary Table 4 presents four RA-related candidate SNP markers within this gene’s promoters, as predicted here.

The human gene *TGFB2* promoter has only one SNP, rs546214861, that can lower the production of transforming growth factor beta 2 (synonym: glioblastoma-derived T-cell suppressor factor) encoded by this gene (Figure 3F), thereby improving the healing of bone-related tissues in inflammatory RA (Um et al., 2018) as a candidate SNP marker of RA alleviation (Supplementary Table 4).

If we look through Supplementary Table 4, there are more (104 vs. 75) candidate SNP markers corresponding to enhancement of (than damage to) TBP-sites of 25 human immunosuppressive genes *BCL6*, *CD4*, *CNMD*, *EBI3*, *FGF21*, *GAS6*, *GDF5*, *DUSP1*, *FGF22*, *FOXP3*, *IL1R2*, *IL2RA*, *IL2RB*, *IL4*, *IL10*, *IL10RA*, *IL10RB*, *IRF2*, *IRF4*, *IRF8*, *PDCD1*, *PIAS1*, *SOCS3*, *TGFB2*, and *TNFRSF8* (Table 2, row 7: *p* < 0.025, binomial distribution). This means natural-selection pressure directed against underexpression of the human immunosuppressive genes, consistently with the abovementioned rows 6 and 7 of Table 2; these rows correspond to genes often associated with RA and immunostimulatory genes, respectively. As for predisposition to RA, contrary to all the abovementioned rows 3, 4, 5, and 6 of Table 2, when summarizing Supplementary Table 4, we were surprised by the significant predominance (109 over 70) of candidate SNP markers corresponding to alleviation over aggravation of RA (*p* < 0.01) binomial distribution rather than vice versa, as presented in row 7 of Table 2. This result indicates that natural-selection pressure on the human immunosuppressive genes is directed toward resistance to RA rather than predisposition to RA presented in rows 3, 4, 5, and 6 of this table. First, within row 7 compared with rows 3 and 4 of Table 2, readers can see two statistically significant genome-wide patterns opposite to each other, namely: (1) neutral drift increasing RA-related risks and (2) natural selection decreasing them; superposition of these patterns can stabilize them and thus establish the normal level of RA-related risks as a human trait. Finally, rows 5, 6, and 7 indicate the only consistently bidirectional natural selection at the whole-genome scale [either (1) for RA resistance in case of immunosuppressive genes or (2) for RA predisposition in case of immunostimulatory and all remaining genes] that can disrupt the RA norm in humans as if self-domestication (Theofanopoulou et al., 2017) has occurred with its disruptive natural selection (Belyaev, 1979). According to the mainstream point of view on Human Origins, there is not enough scientific evidence that this could actually happen. In this work, we verified the bidirectional natural selection on the human genome-wide scale simultaneously for resistance and predisposition to RA during their comparison with publicly available data on DEGs in pets vs. wild animals, as presented below.

### *In vivo* Validation of Our Predictions Using DEGs Within Pets Versus Wild Animals

Here, we compared 68 human genes within whose promoters there are RA-related candidate SNP markers predicted in this work (Supplementary Tables 1–4, the essence of which is Supplementary Table 5) with 1740 DEGs of pets vs. wild animals (Albert et al., 2012; Hekman et al., 2018; Yang et al., 2018). Their description is given in Table 1, as depicted in Figure 2. The obtained results are presented in Table 3.

**TABLE 3:** Comparing the effects of the orthologous-gene expression changes on rheumatoid arthritis (RA) in humans and during the divergence of domestic and wild animals from their nearest common ancestor.

Humans					Animals				
Gene	Effect of gene expression change (Δ) on RA, i.e., either contributes to (↑) or relieves (↓)				*DEG*	*RNA-Seq*		Δ During divergence from the nearest common ancestor	
	Deficit	RA	Excess	RA		log_2_	*P*_*ADJ*_	Deficit	Excess
					Tame vs. aggressive foxes (Hekman et al., 2018)				
*ESR2*	Less ESR2-dependent suppression (Armstrong et al., 2013)	↑	More ESR2-dependent suppression (Armstrong et al., 2013)	↓	*Esr2*	−0.3	0.05	Domestic	Wild
*IL1R2*	More inflammation (Ocsko et al., 2018)	↑	Less inflammation (Ocsko et al., 2018)	↓	*Il1r2*	−0.4	0.05	Domestic	Wild
*IL9R*	Less inflammation judging by fibroblast-like synoviocytes (Raychaudhuri et al., 2018)	↓	More inflammation judging by fibroblast-like synoviocytes (Raychaudhuri et al., 2018)	↑	*Il9r*	0.4	0.05	Wild	Domestic
*NPY*	Lower risk of obesity-caused RA (Stofkova et al., 2009)	↓	Higher risk of obesity-caused RA (Stofkova et al., 2009)	↑	*Npy*	0.4	10^–2^	Wild	Domestic
*TGFB2*	Better recovery from RA (Um et al., 2018)	↓	Inhibited bone repair in RA (Um et al., 2018)	↑	*Tgfb2*	0.5	10^–2^	Wild	Domestic
					Domesticated vs. wild rabbits (Albert et al., 2012)				
*F7*	Higher risk of hemorrhagic forms of RA (Thornorsteinsson et al., 2004)	↑	Recombinant F7 is a drug in hemophilia comorbid with RA (Drobiecki et al., 2013)	↓	*F7*	−2.7	0.05	Domestic	Wild
					Guinea pigs vs. cavies (Albert et al., 2012)				
*CCR6*	Relieved RA (Bonelli et al., 2018)	↓	Higher risk of RA (Jatczak-Pawlik et al., 2020)	↑	*Ccr6*	2.2	0.05	Wild	Domestic
*CETP*	CETP deficiency promotes mortality in RA (Ferraz-Amaro et al., 2013)	↑	Higher risk of RA (Kim et al., 2016)	↑	*Cetp*	2.1	10^–3^	Wild	Domestic
*IL1B*	Relieved RA (Rzepecka et al., 2015)	↓	Circadian pain in RA (Olkkonen et al., 2015)	↑	*Il1b*	2.3	10^–2^	Wild	Domestic
*PDYN*	Pain hypersensitivity (Zheng et al., 2014)	↑	Pain resistance (Zheng et al., 2014)	↓	*Pdyn*	0.9	10^–2^	Wild	Domestic
					Dog vs. wolf (blood) (Yang et al., 2018)				
*HBB*	Thalassemia-related osteoporosis worsens RA (Giakoumi et al., 2005)	↑	Hemolytic-origin extracellular HBB releases thrombogenic heme that adds to RA-related thrombogenesis (Bisoendial et al., 2010; Gall et al., 2018)	↑	*Hbbl*	−5.9	10^–8^	Domestic	Wild

*log_2_: differential expression of a gene of pets normalized to that in wild animals (log_2_ units).*

First of all, as readers can see in Table 3, underexpression of three human genes *ESR2*, *IL1R2*, and *F7* corresponds to attenuated ESR2-dependent suppression (Armstrong et al., 2013), increased inflammation (Ocsko et al., 2018), and hemorrhagic forms of RA (Thornorsteinsson et al., 2004); this pattern is consistent with underexpression of their orthologous animal genes during domestication and *vice versa*. Besides, overexpression of five human genes *CCR6*, *IL9R*, *NPY*, *IL1B*, and *TGFB2* elevates the risk of RA (Jatczak-Pawlik et al., 2020), inflammation (Raychaudhuri et al., 2018), obesity (Stofkova et al., 2009), and circadian pain (Olkkonen et al., 2015) and inhibits bone repair (Um et al., 2018), respectively; these data match the pattern of overexpression of their orthologous genes during animal domestication, and *vice versa*. Additionally, both underexpression and overexpression of two human genes *CETP* and *HBB* contribute to RA, as shown in Table 3. Finally, overexpression of only human gene *PDYN* in our gene set reduces pain sensitivity (Zheng et al., 2014), thus relieving RA, and in only this case in our study corresponds to the orthologous gene’s overexpression during the guinea pig domestication and *vice versa* (Table 3).

Table 4 sums up the findings of the comparative analysis of the above orthologous genes from humans and animals, namely, 10 and 1 of these domestic-animal DEGs were found to correspond to human gene-markers of aggravated and relieved RA, and the same is true for three and eight DEGs in the wild animals. Therefore, the DEGs in domestic animals are significantly consistent with their human orthologous genes that contribute to RA, according to Pearson’s χ^2^ test (*p* < 0.01), Fisher’s exact test (*p* < 0.05), and binomial distribution (*p* < 0.01). Finally, Table 4 indicates that the DEGs of wild animals correspond equally to human orthologous genes that aggravate and relieve RA (*p* > 0.1, binomial distribution) in agreement with the conventional wild-type norm.

**TABLE 4:** Correlations between the effects of codirected changes in the expression of orthologous genes on rheumatoid arthritis (RA) in humans and during the divergence of domestic and wild animals from their nearest common ancestor.

Animals	Humans	Effect of gene expression changes on RA		Binomial distribution	χ^2^-test		Fisher’s exact test, *p*
		Contribute to (↑)	Relieve (↓)		χ^2^	*p*	
Effect of gene expression changes during divergence from the nearest common ancestor	Domestic	10	1	<10^–2^	9	10^–2^	0.05
	Wild	3	8	>0.1			

These findings mean that during domestication, the anthropogenic environment, in contrast to a natural environment, may alter gene expression in the animals on the genome-wide scale (e.g., immunostimulatory and immunosuppressive genes) in a manner that more often contributes to RA than not.

## Conclusion

Because it is best to study TBP-sites genome-wide, we created SNP_TATA_Comparator and applied it to build No. 147 dbSNP of 2016 to predict candidate SNP markers of RA (Chadaeva I. V. et al., 2019) as a cause of disability (Koller and Nobauer-Huhmann, 2009). The robustness of this tool was verified here with the growth of the SNP number, as seen in dbSNP build No. 151 of 2017. Here we analyzed a fourfold higher SNP number allowing us to detect previously unknown whole-genome SNP patterns besides neutral drift (Haldane, 1957; Kimura, 1968) in relation to RA predisposition because diversity of adaptive-immunity memory cells grows with an increase in the number of diseases experienced, thereby enhancing both disease resistance (Sarkander et al., 2016) and the risk of false activation of these cells according to Ashby’s law of requisite variety (Schuldberg, 2015). That is why we additionally investigated both immunostimulatory genes and genes quite often linked to RA, and we noted natural selection against underexpression of these genes in the same direction, toward predisposition to RA. Likewise, we analyzed immunosuppressive genes and surprisingly observed the same natural selection against underexpression of these genes, which nevertheless acts in the opposite direction, toward resistance to RA. Overall, the natural-selection pressure seems bidirectional, e.g.: (1) on immunosuppressive genes toward RA alleviation and (2) on immunostimulatory gene toward RA aggravation, suggesting that self-domestication events (Theofanopoulou et al., 2017) have happened in humans because of the disruptive natural selection (Belyaev, 1979) found in our study. It is common knowledge that the use of gene promoters alone is not enough for the analysis of modes of evolution as a whole. Belyaev (1979) defined the domestication-related disruptive selection as “… what may be selected for are changes in the regulation of genes — that is, in the timing and the amount of gene expression rather than changes in individual structural genes” (Belyaev, 1979). This is a microevolution event when domestic and wild populations of the same species diverge from their nearest common ancestor, to which it is appropriate to compare our human-promoter-based predictions *in silico* (Ponomarenko et al., 2015) and the DEGs of domestic vs. wild animals *in vivo*, as demonstrated recently (Vasiliev et al., 2021).

In accordance with the mainstream opinion that scientific evidence for the human self-domestication is insufficient, we tested our predictions of relief and aggravation in RA using public data on 1740 DEGs in pets vs. wild animals (Albert et al., 2012; Hekman et al., 2018; Yang et al., 2018) as a bioinformatic animal model of human diseases. Among the DEGs examined, this approach yielded 10 and 1 DEGs that correspond to alleviation and aggravation of RA in pets, in contrast to three and 8 DEGs in the case of wild animals. Consequently, during domestication, the anthropogenic habitat conditions in comparison with a natural environment may change gene expression in the animals on the whole-genome scale (e.g., immunostimulatory and immunosuppressive genes) and thus contribute more often to RA according to three independent statistical criteria, such as Pearson’s χ^2^ test (*p* < 0.01), Fisher’s exact test (*p* < 0.05), and the binomial distribution test (*p* < 0.01). This finding allows us to propose RA as a candidate symptom within a self-domestication syndrome (Theofanopoulou et al., 2017). Such syndrome might be considered as a human’s payment with health for the benefits received during evolution.

Besides, the RA-related candidate SNP markers predicted here, which are expected to survive obligatory clinical “case-control” studies in the future, may become useful for physicians for optimizing the treatment of patients according to their individual sequenced genome reducing RA-related risks.

The presented verification of SNP_TATA_Comparator’s predictions vis-à-vis the semiquantitative RNA-Seq data is statistically significant. Therefore, the next step of further comprehensive experimental verification of SNP_TATA_Comparator’s biomedical predictions by means of genome-wide data on QTLs [e.g., in human cardiopathology (Koopmann et al., 2014)] seems to be justified and timely.

## Data Availability Statement

The original contributions presented in the study are included in the article/Supplementary Material, further inquiries can be directed to the corresponding author/s.

## Author Contributions

NAK and VK contributed to study conception. MP wrote the manuscript. EO, IC, PP, DO, and DR contributed to the development and optimization of the software. NVK, ES, ID, and LS contributed to the data analysis. All authors contributed to the article and approved the submitted version.

## Conflict of Interest

The authors declare that the research was conducted in the absence of any commercial or financial relationships that could be construed as a potential conflict of interest.

## Footnotes

1 http://beehive.bionet.nsc.ru/cgi-bin/mgs/tatascan/start.pl

## Supplementary Material

Supplementary File 1 : Supplementary Methods An estimate of the affinity of TATA-binding protein (TBP) for a 70 bp proximal promoter in human genes.

Supplementary File 2 : Supplementary Results Supplementary Tables 1–5. Candidate SNP markers of RA predicted in this work near TBP-binding sites within a promoter of human protein-coding genes.

Supplementary File 3 : Supplementary Keyword Search Supplementary Figure 1. A decision logic of the keyword search for RA-related studies in the PubMed database (Lu, 2011).
