# Supplementary material for: Disruptive Selection of Human Immunostimulatory and Immunosuppressive Genes Both Provokes and Prevents Rheumatoid Arthritis, Respectively, as a Self-Domestication Syndrome
Source: Front Genet. 2021 Jun 22;12:610774. doi: 10.3389/fgene.2021.610774 (PMC8259950; doi:10.3389/fgene.2021.610774)
Supplement: Supplementary File 1 : Supplementary Methods — An estimate of the affinity of TATA-binding protein (TBP) for a 70 bp proximal promoter in human genes. [file Data_Sheet_1.PDF]

Supplementary Material

Disruptive selection of human immunostimulatory and immunosuppressive genes both provokes and prevents rheumatoid arthritis, respectively, as a self-domestication syndrome

Natalya V. Klimova, Evgeniya Oshchepkova, Irina Chadaeva, Ekaterina Sharypova, Petr Ponomarenko, Irina Drachkova, Dmitry Rasskazov, Dmitry Oshchepkov, Mikhail Ponomarenko*, Ludmila Savinkova, Nikolay A. Kolchanov, and Vladimir Kozlov

***Correspondence:** Mikhail Ponomarenko (pon@bionet.nsc.ru)

Supplementary Method

An estimate of the affinity of TATA-binding protein (TBP) for a 70 bp proximal promoter in human genes

The input data are two 70 bp DNA sequences, which are $S_{wt} = \{s^{wt}_{-70}\ldots s^{wt}_{i}\ldots s^{wt}_{-1}\}$ and $S_m = \{s^{min}_{-70}\ldots s^{min}_{i}\ldots s^{min}_{-1}\}$ immediately upstream of the transcription start site (TSS, $s^{wt}_0 = s^{min}_0$, where: $s^{\bullet}_i \in \{a, c, g, t\}$) of the human protein-coding gene's proximal promoter carrying either ancestral (wt) or minor (min) allele, respectively, of a given SNP under study. Within our three-step approximation (Ponomarenko et al., 2008) of the TBP–promoter binding (i.e., TBP slides along DNA ↔ co-recognition between TBP and TBP-site ↔ a DNA bend fixes the TBP–promoter complex) as observed experimentally (Delgadillo et al., 2009), we calculated two $-\ln[K_D(S_\bullet)]$ values in natural-logarithm units (ln-units), which evaluate the TBP–promoter affinity on each DNA sequence ($S_\bullet$) independently from one another, namely:

$$-\ln[K_D(S_\bullet)] = 10.9 - 0.2\ \{\ln[K_{SLIDE}(S_\bullet)K_{STOP}(S_\bullet)K_{BEND}(S_\bullet)]\}, \qquad (1)$$

where K_D is the equilibrium dissociation constant estimate (in moles per liter, M); 10.9 (ln-units) is nonspecific TBP–DNA affinity (10 μM) as measured elsewhere (Hahn et al., 1989); 0.2 is a stoichiometric coefficient of the three-step TBP–promoter binding as explained elsewhere (Ponomarenko et al., 2008).

In Eq. 1, $-\ln[K_{STOP}(S_\bullet)]$ is an estimate of the equilibrium dissociation constant of the mutual recognition between TBP and the best TBP-site that met at the second step among the three steps under consideration, i.e.:

$$\ln[K_{STOP}(S_\bullet)] = MAX_{-70 \le i \le -20;\ k \in \{-1; +1\}}\{\Sigma_{\ i-1 \le j \le i+13}\ w\{i,s^{\bullet}_{j;k}\}, \qquad (2)$$

where $w\{i,s^{\bullet}_j\}$ is Bucher's weight of nucleotide $s^{\bullet}_j$ at the jth position of the TBP-site (Bucher, 1990); k is an indicator of either direct (+1) or complementary (−1) chains of the double-stranded B-helical DNA of the promoter in question.

In Eq. 1, $-\ln[K_{SLIDE}(S_\bullet)]$ is an estimate of the equilibrium dissociation constant of a contact between TBP and the promoter DNA when they slide one over the other at the first step among the three within this model, as follows:

$$\ln[K_{SLIDE}(S_\bullet)] = MEAN_{[\xi - 7;\ \xi + 19];\ k \in \{-1; +1\}}\ (35.1\mu + 0.8[TA]), \qquad (3)$$

where ξ is the position of the best putative TBP-site according to Eq. 2; μ is minor-groove width of the B-helical DNA at this site center as estimated elsewhere (Karas et al., 1996); [TA] is the concentration of dinucleotide TA; 0.8 and 35.1 are linear regression coefficients (Ponomarenko et al., 1999).

In Eq. 1, $-\ln[K_{BEND}(S_\bullet)]$ is an estimate of the equilibrium dissociation constant of complexes between TBP and each of two DNA chains of the TBP-site separately from one another during DNA melting resulting in a bend that stabilizes the TBP–promoter complex (Flatters, Lavery, 1998) at the last step of their binding, namely:

$$\ln[K_{BEND}(S_\bullet)] = MEAN_{[\xi - 7;\xi + 19];\ k \in \{-1; +1\}}\ (0.9[TA, AA, TG, AG] + 2.5[TA, TC, TG] + 14.4), \qquad (4)$$

where 0.9, 2.5, and 14.4 are linear regression coefficients (Ponomarenko et al., 1999).

After that, by examining all the possible substitutions, $s^{\bullet}_j \rightarrow \varphi$, at each jth position among 26 positions of the best TBP-site (Eq. 2), we evaluated standard deviation δ● of the $-\ln[K_D(S_\bullet)]$ values calculated using (Eq. 1), i.e.:

$$\delta_\bullet = \{(\Sigma_{\xi-7 \le j \le \xi}\Sigma_{\varphi \in \{a,c,g,t\}}\ln[K_D(s^{\bullet}_{\xi-7}\ldots\xi\ldots s^{\bullet}_{\xi+19}) / K_D(s^{\bullet}_{\xi-7}\ldots s_j\ldots s^{\bullet}_{\xi+19})]^2\} / (3{*}26)]^{1/2}. \qquad (5)$$

Supplementary Material

On both ancestral S_{wt} and minor S_{min} sequences in question, using Eqs. 1–5 we calculated two $\{-\ln[K_D(S_{wt})] \pm \delta_{wt}\}$ and $\{-\ln(K_D(S_{min})) \pm \delta_{min}\}$ pairs of values, respectively, and, next, Fisher's Z-score (Waardenberg et al., 2015), as

$$Z = \text{abs}\{\ln[K_D(S_{wt})/K_D(S_m))]/[\delta_{wt}^2 + \delta_m^2\}^{1/2}. \quad (6)$$

Finally, by means of a special package of R (Waardenberg et al., 2015), from this Z-value we calculated a *p*-value (probability rate) of the accepted hypothesis "H_0: $K_D(S_{wt}) \neq K_D(S_{min})$." If statistical significance *p* was >0.95, we predicted the following:

IF {INEQUALITY "$K_D(S_{min}) < K_D(S_{wt})$" is statistically significant},

THEN {PREDICTION is "the minor allele being considered causes overexpression of the gene relative to the ancestral allele"};

ELSE [IF {INEQUALITY "$K_D(S_m) > K_D(S_{wt})$" is statistically significant}, (7)

THEN {PREDICTION is "the minor allele being considered causes underexpression of the gene relative to the ancestral allele"},]

OTHERWISE {PREDICTION is "the expression change of this gene is insignificant"}.

Our Web service SNP_TATA_Comparator (Ponomarenko M et al., 2015) presents this prediction in the "Decision" line of the "Result" textbox, while all the intermediate results are in the other lines of this textbox as shown Figure 1c (see Main text).

References

Bucher, P. (1990) Weight matrix descriptions of four eukaryotic RNA polymerase II promoter elements derived from 502 unrelated promoter sequences. *J. Mol, Biol.* **212**, 563-578. doi: 10.1016/0022-2836(90)90223-9

Delgadillo, R.F., Whittington, J.E., Parkhurst, L.K., and Parkhurst, L.J. (2009). The TATA-binding protein core domain in solution variably bends TATA sequences via a three-step binding mechanism. *Biochemistry*. **48**, 1801-1809. doi: 10.1021/bi8018724

Flatters, D. and Lavery, R. (1998) equence-dependent dynamics of TATA-Box binding sites. Biophys J. 75, 372-381. doi: 10.1016/S0006-3495(98)77521-6

Karas H, Knuppel R, Schulz W, Sklenar H, Wingender E. (1996) Combining structural analysis of DNA with search routines for the detection of transcription regulatory elements. *Comput Applic Biosci*. **12**, :441-446. doi: 10.1093/bioinformatics/12.5.441

Ponomarenko, M., Ponomarenko, J., Frolov, A., Podkolodny, N., Savinkova, L., Kolchanov, N., and Overton, G. (1999) Identification of sequence-dependent features correlating to activity of DNA sites interacting with proteins. *Bioinformatics*. **15**, 687-703. doi: 10.1093/bioinformatics/15.7.687

Ponomarenko, P.M., Savinkova, L.K., Drachkova, I.A., Lysova, M.V., Arshinova, T.V., Ponomarenko, M.P., and Kolchanov, N.A. (2008) A step-by-step model of TBP/TATA box binding allows predicting human hereditary diseases by single nucleotide polymorphism. *Dokl Biochem Biophys*. **419**, 88-92. doi: 10.1134/S1607672908020117

Ponomarenko, M., Rasskazov, D., Arkova, O., Ponomarenko, P., Suslov, V., Savinkova, L., et al. (2015). How to use SNP_TATA_Comparator to find a significant change in gene expression caused by the regulatory SNP of this gene's promoter via a change in affinity of the TATA-binding protein for this promoter. *Biomed Res Int.* **2015**: 359835. doi: 10.1155/2015/359835

Waardenberg, A.J., Basset, S.D., Bouveret, R., and Harvey, R.P. (2015). CompGO: an R package for comparing and visualizing Gene Ontology enrichment differences between DNA binding experiments. *BMC Bioinformatics*. **16**:275. doi:10.1186/s12859-015-0701-2.
