# Supplementary material for: Disruptive Selection of Human Immunostimulatory and Immunosuppressive Genes Both Provokes and Prevents Rheumatoid Arthritis, Respectively, as a Self-Domestication Syndrome
Source: Front Genet. 2021 Jun 22;12:610774. doi: 10.3389/fgene.2021.610774 (PMC8259950; doi:10.3389/fgene.2021.610774)
Supplement: Supplementary File 2 : Supplementary Results — Supplementary Tables 1–5. Candidate SNP markers of RA predicted in this work near TBP-binding sites within a promoter of human protein-coding genes. [file Data_Sheet_2.PDF]

frontiers

Supplementary Material

Disruptive selection of the human immunostimulatory and immunosuppressive genes both provoke and prevent rheumatoid arthritis, respectively, as a self-domestication syndrome

Natalya V. Klimova, Evgeniya Oshchepkova, Irina Chadaeva, Ekaterina Sharypova, Petr Ponomarenko, Irina Drachkova, Dmitry Rasskazov, Dmitry Oshchepkov, Mikhail Ponomarenko*, Ludmila Savinkova, Nikolay Kolchanov, and Vladimir Kozlov

***Correspondence:** Mikhail Ponomarenko (pon@bionet.nsc.ru)

Supplementary Results

Table S1. Candidate SNP markers for rheumatoid arthritis (RA) near biomedical SNP markers within TBP-sites

Gene, OMIM ID	dbSNP ID or Gene:mutation	5′ flank	wt	mut	3′ flank	K_D, nM, prediction: wt	mut	Δ	Z	α	ρ	**Known physiological** or *candidate* SNP markers	*RA*	Ref.: **fact** or *hypothesis*
MMP12 601046	**rs2276109$**	**CAACT**	**A**	**g**	**TGAGT**	**11**	**14**	**<**	**3**	**10^{-2}**	**C**	**less srisks to asthma**	↓	**Hunninghake et al, 2009**
	rs572527200$	*TATCA*	*A*	*g*	*CTATG*	*11*	*14*	*<*	*3*	*10^{-2}*	*C*	*lower risk of RA*	↓	*Liu et al., 2004*
	rs1401366377	*ACTAT*	*G*	*a*	*AGTCA*	*11*	*3*	*>*	*20*	*10^{-6}*	*A*	*higher risks of RA*	↑	
SOD1 147450	**rs7277748$**	**GGCCT**	**A**	**g**	**TAAAG**	**2**	**7**	**<**	**17**	**10^{-6}**	**A**	**amyotrophic lateral sclerosis**	↑	**Niemann et al., 2007**
	rs1438766715	*GGGCG*	*26bp*	-	*AGTCG*	*2*	*79*	*<*	*59*	*10^{-6}*	*A*	*higher both risks of RA and pain in RA, whereas bee venom apitoxin is both anti-RA drug within oriental medicine and, a perspective drug to slow motor neuron loss in amyotrophic lateral sclerosis within current alternative medicine*	↑	*Staron et al., 2012; Hemshekhar et al., 2017; Yang, Choi , 2013*
	rs132505255	*CCTAT*	*A*	*g*	*AAGTA*	*2*	*9*	*<*	*22*	*10^{-6}*	*A*		↑	
	rs966452334	*TGGCC*	*T*	*c*	*ATAAA*	*2*	*6*	*<*	*16*	*10^{-6}*	*A*		↑	

Notes: hereinafter, Alleles: wt, ancestral; mut, minor; "−", deletion; K_D, dissociation constant of TBP–DNA complex; α = 1 – p, significance (where p value is given in Figure 1 (see Main Text); **Δ, changes**: excess (>) and deficit (<); **RA, rheumatoid arthritis risks**: increase (↑) and decrease (↓); ρ, heuristic rank of candidate SNP markers varying in alphabetical order from the "best" (A) to the "worst" (E). $)This SNP is in both issues #151 and #147 of database dbSNP corresponding to this study and our previous study (Chadaeva et al., 2019) that is why here we are additionally citing our earlier published results for this SNP (Chadaeva et al., 2019), as labeled for brevity by the "$" symbol superscripted just after the proper dbSNP IDs. *)This SNP also includes other neutral alleles. #)Distance from this SNP to a given alternative transcription start site (TSS) whose transcription activity is altered by this SNP. Reference, the *italicized references* found by our manual keyword search in the PubMed database (Figure S1, Supplementary file 3) the contents of which are *italicized* in the third rightmost column. **Genes**: *ACKR1,* atypical chemokine receptor; *ADH7,* alcohol dehydrogenase 7; *APOA1,* apolipoprotein A1; *CETP,* cholesteryl ester transfer protein; *COMT,* catechol-O-methyltransferase; *DHFR,* dihydrofolate reductase; *ESR2,* estrogen receptor 2 (β); *F3,* coagulation factor III (synonyms: thromboplastin, tissue factor); *F7,* F7, coagulation factor VII (synonyms: proconvertin, serum prothrombin conversion accelerator); *FGFR2,* fibroblast growth factor receptor 2; *GCG,* glucagon; *HBB,* hemoglobin subunits β; *HBD,* hemoglobin subunits δ; *HSD17B1,* hydroxysteroid 17β dehydrogenase 1; *HTR2C,* 5-hydroxytryptamine (serotonin) receptor 2C; *IL1B,* interleukin 1β; *INS,* insulin; *LEP,* leptin *MBL2,* mannose-binding lectin 2 (synonyms: collectin-1); *MLH1,* DNA mismatch repair protein Mlh1; *MMP12,* matrix metallopeptidase 12 (synonym: macrophage elastase); *NOS2,* nitric oxide synthase 2; *PDYN,* prodynorphin; *RET,* Ret proto-oncogene; *SOD1,* superoxide dismutase 1; *TGFBR2,* , transforming growth factor β receptor 2; *TPI1*, triosephosphate isomerase 1;. **Deletion/insertion**, *APOA1*: 6 bp = gacata; *CETP*: 18 bp = gggcggacatacatatac; *COMT*: 15 bp = ccgccacggcctgcg; *DHFR* 35 bp = ctcgcctgcacaaatggggacgaggggggcggggc; *MLH1:* 21 bp = ggatacaacaaaggggacttc; *SOD1:* 26 bp = aggcgcggaggtctggcctataaagt.

Supplementary Material

Table S1. Continued

Gene, OMIM ID	dbSNP ID or Gene:mutation	5′ flank	wt	mut	3′ flank	K_D, nM, prediction						**Known physiological** or *candidate* SNP markers	*RA*	Ref.: **fact** or *hypothesis*
						wt	mut	Δ	Z	α	ρ			
INS 176730	**rs5505**[$]	**ACTGT**	**C**	**t**	**CTTCT**	**53**	**44**	**>**	**4**	**10^{-3}**	**B**	**neonatal diabetes mellitus, hyperinsulinemia**	↑	**Landrum et al., 2014**
	rs1389349459	*TCCCA*	*G*	*t*	*ATCAC*	*53*	*14*	>	*25*	10^{-6}	*A*	*more pain in RA, that is relievable due to both fish-full and Mediterranean diets*	↑	*Abrahamson, 1952; Philippou, Nikiphorou, 2018*
	rs563207167[$]	*CCTGC*	*C*	*t*	*TGTCT*	*53*	*44*	>	*4*	10^{-3}	*B*		↑	
	rs1367101897	*GCCCT*	*G*	*a*	*CCTGT*	*53*	*32*	>	*9*	10^{-6}	*A*		↑	
	rs11557611	*CTGTC*	*C*	*t*	*CTTCT*	*53*	*60*	<	*2*	*0.05*	*D*	*less pain in RA*	↓	
PDYN 131340	**rs886056538**	**AGCAA**	**G**	**a, c**	**GGCTG**	**107**	**94**	**>**	**3**	**0.05**	**D**	**spinocerebellar ataxia**	↓	**Landrum et al., 2014**
	rs1162229303	*GACAG*	*G*	*a*	*GGAGG*	*107*	*59*	>	*12*	10^{-6}	*A*	*increased threshold of pain sensitivity in RA, whereas PDYN deficit lowers it*	↓	*Zheng et al., 2014*
	rs371345545	*TGAGC*	*G*	*a*	*ACAGG*	*107*	*85*	>	*5*	10^{-3}	*B*		↓	
	rs557431815	*CTGAG*	*C*	*a, t*	*GACAG*	*107*	*59*	>	*12*	10^{-6}	*A*		↓	
	rs1184656767	*GCAAG*	*G*	*a*	*GCTGA*	*107*	*93*	>	*3*	10^{-2}	*C*		↓	
	rs1195765727	*CTCAG*	*G*	*t*	*AAGGG*	*107*	*27*	>	*25*	10^{-6}	*A*		↓	
	rs1397278307	*GTGCT*	*C*	*t*	*AGCAA*	*107*	*37*	>	*19*	10^{-6}	*A*		↓	
	rs1253196075	*GCAGC*	*C*	*t*	*TGTGC*	*107*	*45*	>	*16*	10^{-6}	*A*		↓	
	rs1437838635	*AGCAG*	*C*	*a*	*CTGTG*	*107*	*82*	>	*5*	10^{-6}	*A*		↓	
	rs1293511692	*TTCCC*	*C*	*t**	*ATTGG*	*107*	*67*	>	*9*	10^{-6}	*A*		↓	
COMT 116790	**rs370819229**	**CGCCA**	**C**	**a***	**CCGCC**	**187**	**211**	**<**	**2**	**0.05**	**D**	**dilated cardio-myopathy**	↑	**Landrum et al., 2014**
	rs901020754	*CTGCG*	*T*	*c*	*CCGCC*	*187*	*243*	<	*5*	10^{-3}	*B*	*higher chronic pain sensitivity in RA*	↑	*Finan, Zautra, 2013*
	rs779542396	*CGGCC*	*T*	*c*	*GCGTC*	*187*	*243*	<	*5*	10^{-3}	*B*		↑	
	rs45593642	*GGAAG*	*C*	*a*	*GCCCT*	*187*	*115*	>	*9*	10^{-6}	*A*	*lesser chronic pain sensitivity in RA*	↓	
	rs45581136	*CGGAA*	*G*	*a*	*CGCCC*	*187*	*160*	>	*3*	10^{-2}	*C*		↓	
	rs868447575	*GCCAC*	*C*	*a, t*	*GGAAG*	*187*	*74*	>	*17*	10^{-6}	*A*		↓	
	rs1369731401	*CGCCA*	*C*	*a*	*CGGAA*	*187*	*118*	>	*8*	10^{-6}	*A*		↓	
	rs928358205	*TCCGC*	*C*	*t*	*ACCGG*	*187*	*82*	>	*13*	10^{-6}	*A*		↓	
	rs1060501404	*CGTCC*	*G*	*a*	*CCACC*	*187*	*137*	>	*5*	10^{-6}	*A*		↓	
	rs1296549321	*GCGTC*	*C*	*t*	*GCCAC*	*187*	*152*	>	*4*	10^{-3}	*B*		↓	
	rs1333679310	*CCTGC*	*G*	*a*	*TCCGC*	*187*	*110*	>	*10*	10^{-6}	*A*		↓	
	rs981175339	*GCCTG*	*C*	*a, t*	*GTCCG*	*187*	*113*	>	*9*	10^{-6}	*A*		↓	
	rs748298389	*GGCCT*	*G*	*t*	*CGTCC*	*187*	*70*	>	*18*	10^{-6}	*A*		↓	
	rs1428300695	*ACGGC*	*C*	*t**	*TGCGT*	*187*	*117*	>	*9*	10^{-6}	*A*		↓	
	rs562298402	*GCCAC*	*G*	*a*	*GCCTG*	*187*	*150*	>	*4*	10^{-3}	*B*		↓	
	rs1249101844	*CCCGC*	*15bp*	-	*CCGCC*	*187*	*83*	>	*15*	10^{-6}	*A*		↓	
	**rs777650793**	**CCACC**	**G**	**a**	**GAAGC**	**187**	**82**	>	**15**	**10^{-6}**	**A**	**cardiovascular disease**	↓	**Landrum et al., 2014**

Supplementary Material

Table S1. Continued

Gene, OMIM ID	dbSNP ID or Gene:mutation	5′ flank	wt	mut	3′ flank	K_D, nM, prediction						**Known physiological** or *candidate* SNP markers	*RA*	Ref.: **fact** or *hypothesis*
						wt	mut	Δ	Z	α	ρ			
RET 164761	**rs10900296**	**CGCTT**	**A**	**g, c**	**CCTCG**	**30**	**90**	**<**	**20**	**10^{-6}**	**A**	**pheochromocytoma, renal dysplasia**	↑	**Landrum et al., 2014**
	rs551321384	*GCGCT*	*T*	*c*	*ACCTC*	*30*	*75*	<	*17*	10^{-6}	*A*	*pheochromocytoma and RA complicate diagnosis each other*	↑	*Sarin et al., 2014; Bridgewater et al., 2008; Townsend et al., 1994*
	rs1191017949	*GGCGC*	*T*	*c*	*TACCT*	*30*	*48*	<	*7*	10^{-6}	*A*		↑	
	rs1237152255	*CGGCG*	*C*	*t*	*TTACC*	*30*	*20*	>	*6*	10^{-6}	*A*		↑	
	rs1372293149	*CCGGC*	*G*	*a*	*CTTAC*	*30*	*16*	>	*10*	10^{-6}	*A*		↑	
	**rs10900297**	**TACCT**	**C**	**a**	**GCTTC**	**30**	**9**	**>**	**17**	**10^{-6}**	**A**	**pheochromocytoma, renal dysplasia**	↑	**Landrum et al., 2014**
IL1B 147720	**rs1143627[$]**	**AAAGC**	**C**	**t**	**ATAAA**	**5**	**1**	**>**	**15**	**10^{-6}**	**A**	**gastric cancer susceptibility after *Helicobacter pylori* infection**	↑	**Landrum et al., 2014**
												circadian pain in RA		*Olkkonen et al. 2015*
	rs549858786	*GCCAT*	*A*	*t*	*AAAAC*	*5*	*7*	<	*8*	10^{-6}	*A*	*relieved RA*	↓	*Rzepecka et al, 2015*
MLH1 120436	**rs63750527**	**CAAAG**	**G**	**c**	**GGACT**	**11**	**9**	**>**	**4**	**10^{-3}**	**B**	**nonpolyposis colon cancer**	↑	**Landrum et al., 2014**
	**rs756099600**	**ACAAA**	**G**	**a, c**	**GGGAC**	**11**	**10**	**>**	**3**	**0.05**	**D**		↑	
	rs753671152	*GAGGG*	*T*	*a*	*TCAGA*	*11*	*9*	>	*4*	10^{-3}	*B*	*higher comorbid risks of RA*	↑	*Jeong et al., 2017*
	rs1424963586	*AACAA*	*A*	*c*	*GGGGA*	*11*	*9*	>	*3*	10^{-2}	*C*		↑	
	rs752622244	*AGGGG*	*G*	*t*	*ATACA*	*11*	*3*	>	*17*	10^{-6}	*A*		↑	
	rs587778905	*GAGGG*	*21bp*	*taaa*	*AGAAA*	*11*	*19*	<	*9*	10^{-6}	*A*	*higher risk of cancer development in RA as reduced DNA repair*	↑	*Kullmann et al., 2000*
	rs34285587	*ATACA*	*A*	*g, t*	*CAAAG*	*11*	*20*	<	*9*	10^{-6}	*A*		↑	
	rs864622145	*GGGAT*	*A*	*g*	*CAACA*	*11*	*27*	<	*17*	10^{-6}	*A*		↑	
ADH7 600086	**rs17537595**	**TGTTA**	**T**	**c**	**ATACA**	**1**	**3**	**<**	**13**	**10^{-6}**	**A**	**esophageal cancer**	↑	**Abbas et al., 2006**
	rs372329931	*CTGTT*	*A*	*g*	*TATAC*	*1*	*3*	<	*13*	10^{-6}	*A*	*higher comorbid risks of RA as well as those in the case of ADH7 overexpression too*	↑	*Hemminki et al., 2012; Jelski et al., 2008*
	rs755152695	*TGCTG*	*T*	*c*	*TATAT*	*1.0*	*1.4*	<	*4*	10^{-3}	*B*		↑	
	rs1238877951	*AGCTG*	*C*	*a*	*TGTTA*	*1.0*	*1.2*	<	*3*	10^{-2}	*C*		↑	
HSD17B1 109684	**rs201739205**	**ATATC**	**A**	**c**	**AGCCC**	**13**	**18**	**<**	**5**	**10^{-3}**	**B**	**breast cancer**	↓	**Peltoketo et al., 1994**
	rs748743528	*TGATA*	*T*	*c*	*CAAGC*	*13*	*28*	<	*13*	10^{-6}	*A*	*elevated breast cancer risks at both deficit and excess of this enzyme are dystonic reducing RA risks*	↓	*Chen H et al., 2019; He et al., 2016*
	rs779674159	*GTGAT*	*A*	*t*	*TCAAG*	*13*	*35*	<	*18*	10^{-6}	*A*		↓	
	rs1282820277	*CAGGT*	*G*	*a*	*ATATC*	*13*	*7*	>	*9*	10^{-6}	*A*		↓	
	rs755636251	*AGCAG*	*G*	*t*	*TGATA*	*13*	*11*	>	*2*	*0.05*	*D*		↓	
	rs1332869256	*AAGCA*	*G*	*c*	*GTGAT*	*13*	*11*	>	*4*	10^{-3}	*B*		↓	

Table S1. Continued

Gene, OMIM ID	dbSNP ID or Gene:mutation	5′ flank	wt	mut	3′ flank	K_D, nM, prediction: wt	mut	Δ	Z	α	ρ	**Known physiological** or *candidate* SNP markers	*RA*	Ref.: **fact** or *hypothesis*
ESR2 601663	**rs35036378**	**CGGTC**	**T**	**g**	**TTAAA**	**6**	**8**	**<**	**5**	**10^{-3}**	**B**	**ESR2-deficient pT1 breast tumor**	↑	**Philips et al., 2012**
	rs766797386	*AGGAA*	*G*	*t*	*AAGGG*	*6*	*7*	*<*	*3*	*10^{-2}*	*C*	*reduced ESR2-dependent suppression of inflammation in RA*	↑	*Armstrong et al., 2013*
DHFR 126060	**rs10168[$]**	**CAAAT**	**G**	**a**	**GGGAC**	**15**	**9**	**>**	**9**	**10^{-6}**	**A**	*methotrexate resistance in leukemia*	↑	**Al-Shakfa et al., 2009**
	rs750793297[$]	*AAATG*	*G*	*t*	*GGACG*	*15*	*13*	*>*	*3*	*10^{-2}*	*C*	*methotrexate resistance in RA*	↑	
	rs1464445339	*GGGGC*	*35bp*	*-*	*GGCCA*	*15*	*170*	*<*	*44*	*10^{-6}*	*A*		↓	
	rs766799008[$]	*CACAA*	*A*	*g*	*TGGGG*	*15*	*19*	*<*	*3*	*10^{-3}*	*B*		↓	*Bennike et al., 2017*
	rs764508464[$]	*CACAA*	*A*	*-*	*TGGGG*	*15*	*37*	*<*	*17*	*10^{-6}*	*A*	*methotrexate susceptibility in RA*	↓	
	rs754122321[$]	*CTGCA*	*C*	*g*	*AAATG*	*15*	*25*	*<*	*9*	*10^{-6}*	*A*		↓	
	rs1328822484	*CCTGC*	*A*	*g*	*CAAAT*	*15*	*58*	*<*	*25*	*10^{-6}*	*A*		↓	
FGFR2 609579	**rs886046768**	**GCGGT**	**G**	**a**	**GAGAG**	**116**	**31**	**>**	**22**	**10^{-6}**	**A**	**craniosynostosis**	↑	**Landrum et al., 2014**
	rs1212347974	*GAGAG*	*C*	*t*	*GCGGT*	*116*	*99*	*>*	*3*	*10^{-2}*	*C*		↑	
	rs1027484343	*GGAGA*	*G*	*t[*]*	*CGCGG*	*116*	*101*	*>*	*3*	*10^{-2}*	*C*		↑	
	rs1226640384	*GAGGA*	*G*	*c*	*AGCGC*	*116*	*99*	*>*	*3*	*10^{-2}*	*C*	*elevated neovascularization in RA*	↑	
	rs1189849606	*TGGAG*	*G*	*a*	*AGAGC*	*116*	*48*	*>*	*17*	*10^{-6}*	*A*		↑	
	rs971411400	*GGTAA*	*C*	*t[*]*	*AGTCA*	*13*	*11*	*>*	*3*	*10^{-2}*	*C*		↑	*Brown et al., 1996*
	rs778187292	*ATGGT*	*G*	*a*	*GTAAC*	*13*	*7*	*>*	*9*	*10^{-6}*	*A*		↑	
	rs1377663539	*GCGGC*	*T*	*c*	*GGAGG*	*116*	*139*	*<*	*3*	*10^{-2}*	*C*		↓	
	rs751951199	*CCTGT*	*A*	*g*	*TGGTG*	*13*	*30*	*<*	*13*	*10^{-6}*	*A*	*reduced neovascularization in RA*	↓	
	rs757648006	*ATCGC*	*C*	*g*	*TGTAT*	*13*	*17*	*<*	*3*	*10^{-2}*	*C*		↓	
	**rs387906677**	**CTGTA**	**T**	**g**	**GGTGG**	**13**	**27**	**<**	**11**	**10^{-6}**	**A**	**bent bone dysplasia**	↓	**Landrum et al., 2014**
TGFBR2 190182	**rs138010137**	**CGCTG**	**A**	**g**	**GTTGA**	**29**	**39**	**<**	**6**	**10^{-6}**	**A**	**aortic thoracic aneurysm**	↑	**Landrum et al., 2014**
	rs1300366819	*AGTTG*	*A*	*g*	*AGTTG*	*29*	*53*	*<*	*12*	*10^{-6}*	*A*	*disrupted T_{reg}-homeostasis*	↑	*Wang et al., 2018*
	rs1310294304	*TGAGT*	*T*	*a*	*GAAGT*	*29*	*11*	*>*	*17*	*10^{-6}*	*A*	*more proliferation of synovial fibroblast*	↑	*Bira et al., 2005*
NOS2 163730	***NOS2*:–51T→c[$] (Burgner et al., 2003)**	**AATAC**	**T**	**c**	**TCTTG**	**2**	**1**	**>**	**3**	**10^{-2}**	**C**	**malaria resistance**	↑	**Hobbs et al., 2002**
	rs1339255364	*GGGTG*	*A*	*g[*]*	*GTATA*	*1.7*	*2.0*	*<*	*2*	*0.05*	*D*	*RA-related chemotherapy target gene*	↓	*Ohtsuka et al., 2002*

Table S1. Continued

Gene, OMIM ID	dbSNP ID or Gene:mutation	5′ flank	wt	mut	3′ flank	K_D, nM, prediction						**Known physiological** or *candidate* SNP markers	*RA*	Ref.: **fact** or *hypothesis*
						wt	mut	Δ	Z	α	ρ			
HBB 141900	**rs33931746[$]**	**GCATA**	**A**	**g, c**	**AAGTC**	**5**	**11**	**<**	**14**	10^{-6}	**A**	**malaria resistance, thalassemia**	↑	**Martiney et al., 1996**
	**rs34598529[$]**	**GGCAT**	**A**	**g**	**AAAGT**	**5**	**18**	**<**	**24**	10^{-6}	**A**		↑	
	**rs397509430[$]**	**GGGCA**	**T**	**-**	**AAAAG**	**5**	**29**	**<**	**34**	10^{-6}	**A**		↑	
	**rs33980857[$]**	**GGGCA**	**T**	**a,g,c**	**AAAAG**	**5**	**21**	**<**	**27**	10^{-6}	**A**		↑	
	**rs33981098[$]**	**TGGGC**	**A**	**g, c**	**TAAAA**	**5**	**9**	**<**	**10**	10^{-6}	**A**		↑	
	rs281864525[$]	*ATAAA*	*A*	*c**	*GTCAG*	*5*	*7*	<	*7*	10^{-6}	*A*	*thalassemia-related osteoporosis worsens RA*	↑	*Giakoumi et al., 2005*
	rs63750953[$]	*CATAA*	*AA*	*-*	*GTCAG*	*5*	*8*	<	*9*	10^{-6}	*A*		↑	
	rs1160543272:t	*GCTGG*	*G*	*t*	*CATAA*	*4.6*	*5.3*	<	*2*	*0.05*	*D*		↑	
	rs1160543272:a	*GCTGG*	*G*	*a*	*CATAA*	*5*	*4*	>	*3*	10^{-2}	*C*	*hemolytically extracellular hemoglobin releases thrombogenic heme, which adds RA-related thrombogenesis*	↑	*Bisoendial et al., 2010; Gall et al., 2018*
	rs34500389[$]	*CTGGG*	*C*	*t*	*ATAAA*	*5*	*2*	>	*14*	10^{-6}	*A*		↑	
	***HBB*:-27A→t (Collins, Weissman, 1984)**	**GCATA**	**A**	**t**	**AAGTC**	**5**	**3**	**>**	**8**	10^{-6}	**A**	**norm (silent SNP)**	↑	**Collins, Weissman, 1984**
HBD 42000	*rs996092254*	*AGGAC*	*C*	*t*	*AGCAT*	*4.2*	*3.5*	>	*4*	10^{-3}	*B*	*hemolytically extracellular HBB releases thrombogenic heme reinforcing RA-related thrombogenesis*	↑	*Bisoendial et al., 2010; Gall et al., 2018*
	rs1473693473	*AGGCA*	*G*	*t*	*GGCAG*	*4*	*5*	<	*3*	10^{-2}	*C*	*thalassemia-related osteoporosis worsens RA*	↑	*Giakoumi et al., 2005*
	rs34166473[$]	*CAGCA*	*T*	*c*	*AAAAG*	*4*	*12*	<	*18*	10^{-6}	*A*		↑	
	**rs35518301[$]**	**CCAGC**	**A**	**g**	**TAAAA**	**4**	**8**	**<**	**11**	10^{-6}	**A**	**malaria resistance, thalassemia**	↑	**Martiney et al., 1996**
ACKR1 613665	**rs2814778[$]**	**TCTTA**	**T**	**c**	**CTTGG**	**10**	**12**	**<**	**4**	10^{-3}	**B**	**malaria resistance, leukopenia**; *leukemia predictor from RA*	↑	**Michon et al., 2001; Nalls et al., 2008**; *Jones et al., 2006*
	rs1185314734	*CTTAT*	*C*	*g*	*TTGGA*	*10*	*6*	>	*8*	10^{-6}	*A*	*more immune response in RA*	↑	*Smith et al., 2008*
F3 134390	**rs563763767[$]**	**TATAG**	**C**	**t***	**GCGCG**	**3**	**2**	**>**	**6**	10^{-6}	**A**	**myocardial infarction, thrombosis**	↑	**Arnaud et al., 2000**
	rs1439518731	*TTATA*	*-*	*ta*	*GCGCG*	*3*	*1*	>	*12*	10^{-6}	*A*	*elevated comorbid risks of RA*	↑	*Lyberg et al., 1982*
	rs966076891:t	*CGGCC*	*C*	*t*	*TTTAT*	*3*	*2*	>	*6*	10^{-6}	*A*		↑	
	rs966076891:g	*CGGCC*	*C*	*g*	*TTTAT*	*3*	*4*	<	*2*	*0.05*	*D*	*less migration of fibroblast-like synoviocyte*	↓	*Lee et al., 2018*
	rs1190659847	*CCCTT*	*T*	*c*	*ATAGC*	*3*	*12*	<	*19*	10^{-6}	*A*		↓	

Table S1. Continued

Gene, OMIM ID	dbSNP ID or Gene:mutation	5′ flank	wt	mut	3′ flank	K_D, nM, prediction						**Known physiological** or *candidate* SNP markers	RA	Ref.: **fact** or *hypothesis*
						wt	mut	Δ	Z	α	ρ			
F7 613878	**F7:-33A→c[$] Kavlie et al., 2003**	**GAGGC**	**A**	**c**	**GAGAA**	**53**	**62**	**<**	**3**	**10^{-2}**	**C**	**moderate bleeding**	↑	**Kavlie et al., 2003**
	rs749691733[$]	*TTTGC*	*C*	*t*	*CGTCA*	*53*	*66*	*<*	*4*	*10^{-3}*	*B*	*higher risks of hemorrhagic forms of RA*	↑	*Thornorsteinsson et al., 2004*
	rs997515289	*GGCAG*	*A*	*t*	*GAACT*	*53*	*68*	*<*	*5*	*10^{-3}*	*B*		↑	
	rs549591993	*TCAGT*	*C*	*a*	*CCATG*	*53*	*25*	*>*	*13*	*10^{-6}*	*A*	*adjuvant therapy based on exogenous recombinant activated coagulation factor VII (F7) against acquired hemophilia as autoimmune complication of RA*	↓	*Drobiecki et al., 2013*
	rs367732974	*TGCCC*	*G*	*a*	*TCAGT*	*53*	*47*	*>*	*2*	*0.05*	*D*		↓	
	rs777947114	*ACTTT*	*G*	*a*	*CCCGT*	*53*	*19*	*>*	*19*	*10^{-6}*	*A*		↓	
	rs1187329967	*GCAGA*	*G*	*c*	*AACTT*	*53*	*32*	*>*	*9*	*10^{-6}*	*A*		↓	
	rs770113559	*CCTTG*	*G*	*a*	*AGGCA*	*53*	*41*	*>*	*5*	*10^{-6}*	*A*		↓	
	rs781338265	*GTCAC*	*C*	*a[*]*	*CTTGG*	*53*	*30*	*>*	*11*	*10^{-6}*	*A*		↓	
	rs754814507	*CCCAT*	*C*	*t*	*CCTCT*	*53*	*45*	*>*	*3*	*10^{-3}*	*B*		↓	
	rs1296764751	*TCCCC*	*C*	*t*	*ATCCC*	*53*	*46*	*>*	*2*	*0.05*	*D*		↓	
TPI1 190450	**rs1800202[$]**	**CTATA**	**T**	**g**	**AAGTG**	**1**	**4**	**<**	**17**	**10^{-6}**	**A**	**hemolytic anemia, neuromuscular diseases**	↑	**Watanabe et al., 1996; Vives-Corrons et al. 1978**
	rs1386262216	*GCTCT*	*A*	*c, g*	*TATAA*	*1*	*2*	*<*	*8*	*10^{-6}*	*A*	*mimicked RA hindered its early diagnosis in child as well as TPI1-excess increases risks of myocardial infarction comorbid to RA*	↑	*Richardson et al., 1984; Xu et al., 2019; Skielta et al., 2020*
	rs781835924[$]	*CGCTC*	*T*	*c*	*ATATA*	*1*	*2*	*<*	*9*	*10^{-6}*	*A*		↑	
CETP 118470	**rs1427119663**	**GGGCT**	**18bp**	**-**	**GGGCT**	**4**	**7**	**<**	**7**	**10^{-6}**	**A**	**hyperalphalipo-proteinemia** / *higher CETP-deficient mortality in RA*	↑	**Plengpanich et al., 2011** / *Ferraz-Amaro et al., 2013*
	rs1002690375	*ACTTA*	*C*	*t*	*ACACC*	*4*	*2*	*>*	*11*	*10^{-6}*	*A*	*higher risks of RA*	↑	*Kim et al., 2016*
	rs757176551	*ACGGG*	*C*	*g*	*TCCAG*	*4*	*2*	*>*	*10*	*10^{-6}*	*A*		↑	
	rs569033466	*TATAC*	*G*	*a,c,t*	*GGCTC*	*4*	*3*	*>*	*4*	*10^{-3}*	*B*		↑	
	rs1451694749	*ATATA*	*C*	*t*	*GGGCT*	*4*	*2*	*>*	*12*	*10^{-6}*	*A*		↑	
	rs17231520	*TGGGC*	*G*	*a*	*GACAT*	*4*	*2*	*>*	*10*	*10^{-6}*	*A*		↑	
MBL2 154545	**rs72661131[$]**	**TTCTA**	**T**	**c**	**ATAGC**	**2**	**4**	**<**	**12**	**10^{-6}**	**A**	**stroke, variable immune-deficiency, pre-eclampsia**	↑	**Cervera et al., 2010; Boldt et al., 2006; Sziller et al., 2007**
	rs1471733364	*CTATA*	*T*	*g*	*AGCCT*	*2*	*5*	*<*	*15*	*10^{-6}*	*A*	*infection susceptibility in RA*	↑	*Nisihara et al., 2016*
	rs562962093[$]	*TTTCT*	*A*	*g*	*TATAG*	*2*	*5*	*<*	*15*	*10^{-6}*	*A*		↑	
	rs567653539[$]	*ATATA*	*G*	*a*	*CCTGC*	*2*	*1*	*>*	*4*	*10^{-3}*	*B*	*RA-caused cardiovascular events*	↑	*Troelsen et al., 2010*

Supplementary Material

Table S1. Continued

Gene, OMIM ID	dbSNP ID or Gene:mutation	5′ flank	wt	mut	3′ flank	K_D, nM, prediction: wt	mut	Δ	Z	α	ρ	**Known physiological** or *candidate* SNP markers	RA	Ref.: **fact** or *hypothesis*
HTR2C 312861	**rs3813929**	**CATCC**	**C**	**t**	**CGTTT**	**73**	**56**	**>**	**6**	**10^{-6}**	**A**	**olanzapine-caused obesity**	↑	**Landrum et al., 2014**
	rs1348095721	*GTGGT*	*G*	*a*	*CAGAT*	*73*	*56*	>	*5*	*10^{-3}*	*B*	*high risks of RA with obstructive sleep apnea, whereas HTR2C-deficit can contrary reduce both serotonin- and cortisol-induced adipogenesis within both pre-adipocytes and macrophages that can finally reduce risks of the obesity-related RA*	↑	*Jagannathan et al., 2017; Priyadarshini et al., 2018*
	rs1444133212	*TTGGC*	*C*	*t*	*CAAGA*	*73*	*39*	>	*13*	*10^{-6}*	*A*		↑	
	rs886838672	*TTTGG*	*C*	*t*	*CCAAG*	*73*	*34*	>	*15*	*10^{-6}*	*A*		↑	
	rs1376972872	*CTTTT*	*G*	*t*	*GCCAA*	*73*	*43*	>	*11*	*10^{-6}*	*A*		↑	
	rs1222709869	*CCTCC*	*C*	*t*	*CTCAT*	*73*	*50*	>	*8*	*10^{-6}*	*A*		↑	
APOA1 107680	**APOA1:-35A→c$ (Matsunaga et al. 1999)**	**ACATA**	**A**	**c**	**ATAGG**	**3**	**4**	**<**	**5**	**10^{-6}**	**A**	**hematuria, fatty liver, obesity**	↑	**Matsunaga et al. 1999**
	rs1428975217	*AATAG*	*G*	*t*	*CCCTG*	*2.6*	*3.0*	<	*2*	*0.05*	*D*	*cardiovascular events in RA, as well as APOA1 excess causes obesity worsening RA too*	↑	*Kokkonen et al.,, 2017; Mendez-Lara et al., 2019*
	rs1017922094	*ATAAA*	*T*	*c*	*AGGCC*	*3*	*6*	<	*9*	*10^{-6}*	*A*		↑	
	rs1297144980	*CTGCA*	*6bp*	*-*	*AATAG*	*3*	*21*	<	*30*	*10^{-6}*	*A*		↑	

Table S2. Candidate SNP markers for RA predicted here near TBP-sites in promoters of the human protein-coding genes that are most often independently associated with RA

Gene, OMIM ID	dbSNP (Sherry et al., 2001)	5′ flank	wt	mut	3′ flank	K_D, nM, prediction						*Candidate SNP markers*	RA	Reference *(hypothesis)*
						wt	mut	Δ	Z	α	ρ			
NPY 162640	*rs1035054020*	CCTGG	*G*	*t*	TTCTC	*89*	*68*	>	*5*	10^{-6}	*A*	*elevated risks of obesity comorbid to RA*	↑	*Stofkova et al., 2009*
	rs1004017675	CTCCT	*G*	*a*	GGTTC	*89*	*28*	>	*20*	10^{-6}	*A*		↑	
	rs902614336	GGTCG	*T*	*c*	AGCCA	*89*	*80*	>	*2*	*0.05*	*D*		↑	
	rs139801169	AGGTG	*G*	*a, t*	TGCTA	*89*	*61*	>	*7*	10^{-6}	*A*		↑	
	rs893676518	TCCAT	*A*	-	GAAGC	*6*	*10*	<	*10*	10^{-6}	*A*	*reduced risks of obesity comorbid to RA*	↓	
	rs951574217	TCCAT	*A*	*t*	GAAGC	*6*	*9*	<	*70*	10^{-6}	*A*		↓	
	rs1223788416	GCTCC	*A*	*g*	TAAAA	*6*	*11*	<	*10*	10^{-6}	*A*		↓	
	rs528363143	ACTCC	*T*	*c*	GGGTT	*89*	*102*	<	*3*	*0.05*	*D*		↓	
HLA-A 142800	*rs1052746596*	TCTCC	*C*	*t*	TTGTT	*37*	*28*	>	*6*	10^{-6}	*A*	*reduced NK-cell activity in RA*	↓	*Zhang et al., 2007*
	rs375076455	TTTCT	*C*	*a*[*]	CCTTG	*37*	*20*	>	*10*	10^{-6}	*A*		↓	
	rs776909339[S]	CATGA	*G*	*a*	TGACA	*22*	*18*	>	*4*	10^{-2}	*C*		↓	
	rs919504401	TTCAT	*G*	*a*	AGTGA	*22*	*8*	>	*18*	10^{-6}	*A*		↓	
	rs976764801	CACAA	*T*	*a*[*]	TCATG	*22*	*14*	>	*8*	10^{-6}	*A*		↓	
	rs1226503323	AAAGC	*C*	*t*	CGCAC	*12*	*14*	<	*3*	10^{-2}	*C*	*less autoimmunity in RA*	↓	*Rutten et al., 2014*
	rs1310349654	GTTCT	*A*	*g*	AAGCC	*12*	*44*	<	*23*	10^{-6}	*A*		↓	
	rs9260120[$]	TCGCT	*G*	*c*[*]	TTCTA	*12*	*14*	<	*3*	10^{-2}	*C*		↓	
	rs41560714	GTCGC	*T*	*g*	GTTCT	*12*	*14*	<	*3*	10^{-2}	*C*		↓	
	rs369883779[$]	TCCCT	*T*	*g*	GTTTC	*37*	*46*	<	*4*	10^{-3}	*B*		↓	
PTPN22 600716	*rs1391701646*	AGTCA	*G*	*a*	TAAAC	*18*	*12*	>	*6*	10^{-6}	*A*	*reduced citrullination-induced RA*	↓	*Chang et al., 2016*
	rs185537537[$]	GTTAC	*G*	*a*	TACTA	*4*	*3*	>	*4*	10^{-3}	*B*		↓	
	rs975380045	GGTTA	*C*	*t*[*]	GTACT	*4*	*2*	>	*5*	10^{-3}	*B*		↓	
	rs375600763[$]	GTACT	*A*	-	ATTTC	*3.6*	*4.4*	<	*2*	*0.05*	*D*	*reduced mannan-induced RA*	↓	*Sood et al., 2016*
	rs1227652996	GTACT	*A*	*g*	ATTTC	*3.6*	*4.8*	<	*3*	10^{-2}	*C*		↓	
PADI4 605347	*rs909387080*	TGGGC	*G*	*t*[*]	ATATA	*3*	*1*	>	*13*	10^{-6}	*A*	*citrullinated protein excess*	↑	*Seri et al., 2014*
	rs1003605669	CGATA	*T*	*g*	AAAGG	*3*	*14*	<	*20*	10^{-6}	*A*	*reduced arthritis severity*	↓	*Seri et al., 2015*
CCR6 601835	*rs1433814180*	GAATA	*A*	*c*	AACTT	*6*	*11*	<	*9*	10^{-6}	*A*	*relieved adaptive immunity-driven RA*	↓	*Bonelli et al., 2018*
	rs1047738754	CTGAG	*G*	*a*	AATAA	*6*	*7*	<	*2*	*0.05*	*D*		↓	
STAT4 600558	*rs1283135938*	CTTTG	*G*	*t*	ACTTG	*33*	*5*	>	*28*	10^{-6}	*A*	*more inflammation in RA*	↑	*Walker et al., 2006*
	rs951107380	TCCTA	*G*	*a*	GGACT	*33*	*27*	>	*3*	10^{-2}	*C*		↑	
	rs1341622257	TTCTC	*C*	-	TAGGG	*33*	*21*	>	*7*	10^{-6}	*A*		↑	
	rs1433079128	AGCGG	*C*	*t*	TTTCC	*33*	*25*	>	*5*	10^{-6}	*A*		↑	
	rs577074531	GGCTA	*C*	*t*	TCAAG	*18*	*13*	>	*5*	10^{-3}	*B*		↑	
	rs1388334044	ACAGG	*C*	*t*	TGATG	*15*	*18*	<	*3*	10^{-2}	*C*	*less severe RA*	↓	*Hildner et al., 2007*
	rs1294769632	AGGCT	*A*	*t*	CTCAA	*18*	*37*	<	*10*	10^{-6}	*A*		↓	

Notes: hereinafter, see under Table S1. **Genes**: *CCR6,* C-C motif chemokine receptor 6*; CTLA4,* cytotoxic T-lymphocyte associated protein 4*; HLA-A,* major histocompatibility complex, class I, A (synonym: leukocyte antigen class I-A); *IL23R,* interleukin 23 receptor*; IRF5,* interferon regulatory factor 5; *NPY,* neuropeptide Y; *PADI4,* peptidyl arginine deiminase 4*; PTPN22,* non-receptor protein tyrosine phosphatase type 22 (synonym: lymphoid-specific protein tyrosine phosphatase); *STAT4,* signal transducer and activator of transcription 4*; TRAF1,* TNF receptor associated factor 1.

Supplementary Material

Table S2. Continued

Gene, OMIM ID	dbSNP (Sherry et al., 2001)	5′ flank	wt	mut	3′ flank	K_D, nM, prediction: wt	mut	Δ	Z	α	ρ	*Candidate SNP markers*	RA	Reference *(hypothesis)*
	rs1410850640	GCGGC	*G*	*a*	GGATG	*40*	*32*	>	*4*	10^{-3}	*B*		↑	
	rs1368034324	GCTGC	*C*	*t*	CAGGG	*168*	*144*	>	*3*	10^{-2}	*C*		↑	
	rs1408548096	AGCTG	*C*	*t*	CCAGG	*168*	*100*	>	*9*	10^{-6}	*A*		↑	
	rs910572649	CAGCA	*G*	*t*	CAGCT	*168*	*82*	>	*13*	10^{-6}	*A*		↑	
	rs6952685	CACTG	*C*	*t*	CCGCC	*168*	*100*	>	*9*	10^{-6}	*A*		↑	
	rs1246089765	GCACT	*G*	*t*	CCCGC	*168*	*123*	>	*6*	10^{-6}	*A*		↑	
	rs1408012180	AAGAC	*G*	*a*	CGGAA	*82*	*54*	>	*9*	10^{-6}	*A*		↑	
	rs912096771	AAGCT	*G*	*a*	CCTGT	*16*	*13*	>	*3*	10^{-2}	*C*		↑	
IRF5	*rs1010860505*	CTTTC	*G*	*a*	TTTCC	*40*	*26*	>	*9*	10^{-6}	*A*	*more inflammation in RA*	↑	*Duffau et al.,*
607218	*rs1352195161*	ACTTT	*C*	*t*	GTTTC	*40*	*19*	>	*16*	10^{-6}	*A*		↑	*2015*
	rs1393549039	TCTAG	*C*	*t*	CACTT	*40*	*31*	>	*5*	10^{-6}	*A*		↑	
	rs3807306	GTCCC	*G*	*t**	GTCTA	*40*	*32*	>	*4*	10^{-3}	*B*		↑	
	rs1474165624	TGTCC	*C*	*t*	GGTCT	*40*	*35*	>	*3*	10^{-2}	*C*		↑	
	rs1295297436	CAAGA	*C*	*a*	GCGGA	*168*	*143*	>	*3*	10^{-2}	*C*		↑	
	rs898011964	CAGGG	*G*	*t*	TGTAG	*30*	*18*	>	*8*	10^{-6}	*A*		↑	
	rs1188361892	CCAGG	*G*	*t*	GTGTA	*30*	*12*	>	*13*	10^{-6}	*A*		↑	
	rs1378990920	GCTCT	*G*	*a*	AGGGA	*35*	*30*	>	*3*	10^{-2}	*C*		↑	
	rs3807135	GGGTG	*T*	*c*	AGGCA	*30*	*95*	<	*19*	10^{-6}	*A*	*less inflammation in RA*	↓	
	rs1345704660	AAAGT	*G*	*a*	AGTGC	*13*	*11*	>	*2*	*0.05*	*D*		↑	
	rs1388701964	TAAAA	*T*	*c*	AGTTG	*5*	*4*	>	*2*	*0.05*	*D*		↑	
	rs760200781	TTTAT	*C*	-	AGAGG	*8*	*4*	>	*8*	10^{-6}	*A*		↑	
	rs1426903117	AATAA	*G*	*a*	TCTCA	*8*	*7*	>	*3*	10^{-3}	*B*	*more inflammation in RA*	↑	
	rs1054563192	CTGAC	*C*	*t*	ACAGT	*33*	*17*	>	*10*	10^{-6}	*A*		↑	
IL23R	*rs79459911*	CACAA	*C*	*a*	ACCCT	*33*	*19*	>	*10*	10^{-6}	*A*		↑	*Quiniou et*
607562	*rs1406396191*	ATCTA	*A*	*g*	AGTGA	*13*	*26*	<	*11*	10^{-6}	*A*		↓	*al., 2014*
	rs1293073335	AATCT	*A*	*g*	AAGTG	*13*	*25*	<	*12*	10^{-6}	*A*		↓	
	rs1334608905	CTAAA	*T*	*c*	AAAAT	*5*	*6*	<	*2*	*0.05*	*D*	*less inflammation in RA*	↓	
	rs1188153460	ATAAG	*T*	*a*	CTCAC	*8*	*10*	<	*3*	10^{-3}	*B*		↓	
	rs1200410336	AGTGA	*A*	*g*	TAAGT	*8*	*12*	<	*5*	10^{-3}	*B*		↓	
	rs1392885494	ATCAC	*A*	*g*	ACACC	*33*	*49*	<	*8*	10^{-6}	*A*		↓	
	rs1395169663	CGGTG	*C*	*t*	GGCTG	*157*	*114*	>	*6*	10^{-6}	*A*		↑	
TRAF1	*rs1378962428*	TGCCG	*G*	*a*	TTTCC	*157*	*107*	>	*7*	10^{-6}	*A*		↑	*Shu et al.,*
601711	*rs1379816130*	CTTGC	*C*	*t**	GGTTT	*157*	*70*	>	*15*	10^{-6}	*A*	*more inflammation in RA*	↑	*2019*
	rs891537415	GCCCT	*G*	*a*	CCTTG	*157*	*45*	>	*22*	10^{-6}	*A*		↑	
	rs1432313112	AGAGC	*C*	*t**	CCCCC	*56*	*50*	>	*2*	*0.05*	*D*		↑	
	rs769380431	TACTT	*C*	*t*	CTGAA	*24*	*14*	>	*8*	10^{-6}	*A*		↑	
	rs1173196597	GTTTT	*G*	*t*	CTCTA	*24*	*17*	>	*5*	10^{-6}	*A*	*overlapping*	↑	*AlFadhli,*
	rs1271929246	GAGAA	*C*	*t*	ATATC	*10*	*5*	>	*10*	10^{-6}	*A*	*autoimmunity in RA*	↑	*2013*
CTLA4	*rs1327060988*	CTATC	*A*	*g*	TAACC	*10*	*7*	>	*6*	10^{-6}	*A*		↑	
123890	*rs1438289096*	TTTGC	*T*	*c*	AAGGT	*24*	*31*	<	*4*	10^{-3}	*B*		↑	
	rs561368432	TATCA	*T*	*c*	AACCT	*10*	*12*	<	*5*	10^{-6}	*A*	*higher autoimmune*	↑	*Alissafi et al.,*
	rs1297671328	CTATC	-	*tg*	ATAAC	*10*	*12*	<	*5*	10^{-6}	*A*	*responses in RA*	↑	*2017*
	rs1274158310	ATTCT	*A*	*g*	TCATA	*10*	*11*	<	*3*	10^{-2}	*C*		↑	

Supplementary Material

Table S3. Candidate SNP markers for RA predicted here near TBP-sites in promoters of the human protein-coding immunostimulatory genes

Gene, OMIM ID	dbSNP (Sherry et al., 2001)	5′ flank	wt	mut	3′ flank	K_D, nM, prediction						*Candidate SNP markers*	RA	Reference *(hypothesis)*
						wt	mut	Δ	Z	α	ρ			
IL9R 300007	*rs56317732*	*ACTTA*	*G*	*a*	*AAGAT*	*12*	*8*	<	*5*	10^{-6}	*A*	*more both fibroblast like synoviocyte proliferation and inflammation in RA*	↑	*Raychaudhuri et al., 2018*
	rs945044791	*GCTGC*	*A*	*g*	*GTTAT*	*10*	*15*	>	*6*	10^{-6}	*A*	*less both fibroblast like synoviocyte proliferation and inflammation in RA*	↓	
NFKB1 164011	*rs923817199*	*TGTAT*	*G*	*a*	*CTCTC*	*11*	*3*	>	*16*	10^{-6}	*A*	*more inflammation in RA*	↑	*Cutolo et al., 2004*
	rs1415516821	*TCTGT*	*C*	*t*	*TGTAT*	*11*	*7*	>	*6*	10^{-6}	*A*		↑	
	rs1189757503	*TTCCT*	*C*	*t*	*ATTCC*	*93*	*53*	>	*10*	10^{-6}	*A*		↑	
	rs1476734145	*ACTTC*	*C*	*t*	*TCATT*	*93*	*44*	>	*14*	10^{-6}	*A*		↑	
	rs1195510589	*GACTT*	*C*	*t*	*CTCAT*	*93*	*41*	>	*15*	10^{-6}	*A*		↑	
	rs917763565	*CCCTC*	*C*	*t*	*AGCCC*	*93*	*80*	>	*3*	*0.05*	*D*		↑	
	rs1345678021	*TTCCC*	*G*	*a*	*TCGGT*	*109*	*94*	>	*3*	10^{-2}	*C*		↑	
	rs1399392367	*GACAG*	*C*	*g*	*TTCCC*	*109*	*92*	>	*3*	10^{-2}	*C*		↑	
	rs912882626	*GCTGA*	*C*	*t*	*AGCTT*	*109*	*35*	>	*20*	10^{-6}	*A*		↑	
	rs927245643	*CCACC*	*G*	*a**	*GAGCG*	*109*	*77*	>	*6*	10^{-6}	*A*		↑	
	rs1446918654:t	*TTCCA*	*C*	*t*	*TTATA*	*1.5*	*1.0*	>	*6*	10^{-6}	*A*		↑	
	rs1446918654:g	*TTCCA*	*C*	*g*	*TTATA*	*1.5*	*1.9*	<	*3*	10^{-2}	*C*	*reduced inflammatory erosion in RA*	↓	*Torices et al., 2016*
	rs560591323	*CTTAT*	*A*	*g*	*GTAGT*	*1*	*5*	<	*17*	10^{-6}	*A*		↓	
	rs747770765	*ACTTA*	*T*	*c*	*AGTAG*	*1*	*5*	<	*16*	10^{-6}	*A*		↓	
	rs913226216	*CCACT*	*T*	*g*	*ATAGT*	*1*	*5*	<	*15*	10^{-6}	*A*		↓	
	rs902291349	*GTTCC*	*A*	*g*	*CTTAT*	*1*	*3*	<	*11*	10^{-6}	*A*		↓	
	rs556792045	*CTAAA*	*T*	*a*	*ATACT*	*3*	*7*	<	*14*	10^{-6}	*A*		↓	
ATF3 603148	*rs768466312*	*TGTGT*	*C*	*g, t*	*TTTCA*	*21*	*18*	>	*3*	10^{-2}	*C*	*risks of RA-related lung diseases*	↑	*Wu et al., 2019*
	rs975505699	*AAGTG*	*G*	*a*	*TCTTT*	*21*	*14*	>	*8*	10^{-6}	*A*		↑	
	rs1266144397	*TTTGG*	*G*	*a*	*TTTCA*	*21*	*17*	>	*4*	10^{-3}	*B*		↑	
	rs536691683	*ACAGT*	*T*	*c*	*TGGGT*	*21*	*18*	>	*3*	*0.05*	*D*		↑	
	rs748753911	*TTTGC*	*C*	*t*	*ATCCA*	*29*	*25*	>	*2*	*0.05*	*D*		↑	
	rs1446388774	*TTTGT*	*C*	*g, t*	*AAGGA*	*29*	*25*	>	*3*	10^{-2}	*C*		↑	
	rs764156991	*CTAAC*	*C*	*t*	*TGACG*	*29*	*22*	>	*5*	10^{-3}	*B*		↑	
	rs1338990305	*CTCCT*	*C*	*a, t*	*CACCC*	*102*	*85*	>	*3*	10^{-2}	*C*		↑	
	rs947211386	*TCTCT*	*C*	*t*	*CACCC*	*102*	*66*	>	*8*	10^{-6}	*A*		↑	
	rs964096773	*GCCCC*	*CTCT*	*-*	*CTCCA*	*102*	*55*	>	*10*	10^{-6}	*A*		↑	
	rs1247566015	*GTTTC*	*A*	*g*	*ATGTG*	*21*	*26*	<	*5*	10^{-3}	*B*	*extra virgin olive oil relieves RA because of it contains natural ATF3 inhibitor*	↓	*Rosillo et al., 2014*
	rs1326470949	*AATAT*	*A*	*g*	*GTAAC*	*2*	*6*	<	*15*	10^{-6}	*A*		↓	
	rs1016221009	*CAGAC*	*A*	*c*	*AACAG*	*22*	*61*	<	*14*	10^{-6}	*A*		↓	
	rs983061156	*CCGGC*	*CAGA*	*-*	*CAAAC*	*22*	*71*	<	*17*	10^{-6}	*A*		↓	
	rs1254617345	*TCGGC*	*-*	*21bp*	*CCCGC*	*102*	*191*	<	*11*	10^{-6}	*A*		↓	
	rs947211386	*TCTCC*	*A*	*g*	*CCCGC*	*102*	*133*	<	*5*	10^{-3}	*B*		↓	
	rs1480121382	*GCCCC*	*C*	*g*	*TCTCT*	*102*	*123*	<	*3*	10^{-3}	*B*		↓	

Notes: hereinafter, see under Table S1. TSS, transcription start site. **Genes**: *ATF3*, activating transcription factor 3; *CCR7*, C-C motif chemokine receptor 7; *IL3RA*, interleukin 3 receptor subunit α; *IL9R*, interleukin 9 receptor; *IL25*, interleukin 25*;* *LCK*, LCK proto-oncogene (synonym: lymphocyte-specific protein-tyrosine kinase); *NFKB1*, nuclear factor kappa B subunit 1; *ZBTB38*, Zinc finger and BTB domain containing protein 38. **Deletion/insertion**, *LCK*: 9bp = cctccggag; 13bp = ggagccctccgga.; *ATF3*: 21bp = gccttggcccctcctccaccc; *ZBTB38:* ins_1 = gagcc, ins_2 = cgggc, ins_3 = ggggc.

Table S3. Continued

Gene, OMIM ID	dbSNP (Sherry et al., 2001)	5′ flank	wt	mut	3′ flank	K_D, nM, prediction						*Candidate SNP markers*	R A	Reference *(hypothesis)*
						wt	mut	Δ	Z	α	ρ			
IL3RA 430000	*rs1239446017*	GAAAG	*A*	*g*	GTCTT	*18*	*15*	>	*4*	10^{-3}	*B*	*more local inflammation of the synovial tissue in RA*	↑	*Cavanagh et al., 2005*
	rs1483581212	TTTCA	*A*	*g*	AGAAA	*18*	*16*	>	*2*	*0.05*	*D*		↑	
	rs1291775566	GAACA	*T*	*c*	GATAA	*18*	*14*	>	*5*	10^{-3}	*B*		↑	
	rs1435920351	CATGA	*T*	-	AATTT	*18*	*27*	<	*8*	10^{-6}	*A*	*lower risk of RA conversion into cancer*	↓	*Wang et al., 2019*
	rs1458842073	TATAA	*A*	*g*	ACAGC	*3*	*4*	<	*7*	10^{-6}	*A*		↓	
ZBTB38 612218	*rs778590465*	GATAA	*G*	*a*	AAGCC	*7*	*5*	>	*7*	10^{-6}	*A*	*aggravates autoimmune RA*	↑	*Ocsko et al., 2018*
	rs1025269786	AGTAT	*G*	*a*	GGCAT	*20*	*8*	>	*13*	10^{-6}	*A*		↑	
	rs1447537130	ATTGT	*T*	*a*	GCATT	*20*	*14*	>	*7*	10^{-6}	*A*		↑	
	rs1263462599	ATGAT	*G*	*a*	TCACA	*20*	*14*	>	*5*	10^{-6}	*A*		↑	
	rs532126801	GTTTT	*C*	*t*	GTTAG	*28*	*11*	>	*17*	10^{-6}	*A*		↑	
	rs1227823977	TGTGA	*G*	*a*	ACCAG	*28*	*21*	>	*6*	10^{-6}	*A*		↑	
	rs1291598152	CATTG	*G*	*a*	TGTGA	*28*	*19*	>	*8*	10^{-6}	*A*		↑	
	rs151105230	AACTG	*C*	*t*	AAGTC	*12*	*8*	>	*7*	10^{-6}	*A*		↑	
	rs767373571	TATCT	*G*	*a**	ATTTT	*10*	*5*	>	*9*	10^{-6}	*A*		↑	
	rs891712734	GTTTT	*C*	*t*	CTTTA	*10*	*8*	>	*3*	10^{-2}	*C*		↑	
	rs986426568	CAACA	*C*	*t*	ACCAC	*25*	*20*	>	*4*	10^{-3}	*B*		↑	
	rs955002180	TTCAA	*C*	*t*	ACACC	*25*	*19*	>	*4*	10^{-3}	*B*		↑	
	rs1167231089	ACTCT	*C*	-	AGAGG	*25*	*15*	>	*10*	10^{-6}	*A*		↑	
	rs1424441275	GAACT	*C*	-	TCAGA	*25*	*22*	>	*3*	*0.05*	*D*		↑	
	rs959635147	GGCCG	*G*	*a*	CAGAG	*131*	*84*	>	*8*	10^{-6}	*A*		↑	
	rs961412733	CTGCC	*C*	*t*	CGCGC	*131*	*114*	>	*3*	10^{-2}	*C*		↑	
	rs1216293307	CCTCT	*G*	*a*	CCCCG	*131*	*45*	>	*17*	10^{-6}	*A*		↑	
	rs568652489	TGCGT	*C*	*t*	CTCTG	*131*	*61*	>	*14*	10^{-6}	*A*		↑	
	rs557322024	TGTTC	*C*	*t*	CTCCT	*26*	*22*	>	*4*	10^{-3}	*B*		↑	
	rs896493109	GTGTA	*T*	*c*	GTGAC	*7*	*6*	>	*2*	*0.05*	*D*		↑	
	rs1015519567	ATAAC	*G*	*a*	GCTGC	*16*	*10*	>	*6*	10^{-6}	*A*		↑	
	rs989809780:a	TTTTC	*G*	*a*	TTAGA	*28*	*12*	>	*14*	10^{-6}	*A*		↑	
	rs989809780:c	TTTTC	*G*	*c*	TTAGA	*28*	*36*	<	*3*	10^{-2}	*C*	*higher chance of apoptosis, which can relieve RA*	↓	*Oikawa et al., 2008*
	rs1445907016	GAGAT	*AAAG*	-	AAGCC	*7*	*9*	<	*4*	10^{-3}	*B*		↓	
	rs770718486	TTTAC	*A*	*c*	CTGCT	*5*	*7*	<	*4*	10^{-3}	*B*		↓	
	rs946866767	CATTT	*T*	*c*	CTGGG	*31*	*64*	<	*15*	10^{-6}	*A*		↓	
	rs1044345370	TTCGT	*T*	*c*	AGAGC	*28*	*36*	<	*5*	10^{-6}	*A*		↓	
	rs576532840	TGTTT	*A*	*g*	CCTTT	*12*	*14*	<	*4*	10^{-3}	*B*		↓	
	rs189171158	GCTCC	*G*	*a**	AGCTT	*25*	*30*	<	*3*	10^{-2}	*C*		↓	
	rs1277931326	CGGCA	-	*ins*$_1$	GAGCC	*131*	*158*	<	*9*	10^{-6}	*A*		↓	
	rs1317203791	GGGGG	-	*ins*$_2$	CGGGG	*131*	*158*	<	*9*	10^{-6}	*A*		↓	
	rs1270743645	CGCCG	-	*ins*$_3$	GGGGC	*131*	*158*	<	*9*	10^{-6}	*A*		↓	
	rs1005714559	TCCTC	*T*	*c*	GCCCC	*131*	*209*	<	*9*	10^{-6}	*A*		↓	
	rs952541747	CGTCC	*T*	*c*	CTGCC	*131*	*248*	<	*12*	10^{-6}	*A*		↓	
	rs767510274	CTTTG	*T*	*c*	TCCCT	*26*	*29*	<	*3*	10^{-3}	*B*		↓	
	rs1009536083	CTAGA	*G*	*c*	GGCGG	*32*	*39*	<	*3*	10^{-2}	*C*		↓	
	rs932043254	GTGCT	*A*	*g*	GAGGG	*32*	*77*	<	*15*	10^{-6}	*A*		↓	
	rs892531639	GGCTG	*T*	*c*	GCTAG	*32*	*58*	<	*9*	10^{-6}	*A*		↓	
	rs570189743	TGTGT	*A*	*t*	TGTGA	*7*	*9*	<	*4*	10^{-3}	*B*		↓	
	rs1467246162	CGGCT	*G*	*t*	CCACT	*16*	*19*	<	*3*	10^{-3}	*B*		↓	

Table S3. Continued

Gene, OMIM ID	dbSNP (Sherry et al., 2001)	5′ flank	wt	mut	3′ flank	K_D, nM, prediction: wt	mut	Δ	Z	α	ρ	*Candidate SNP markers*	RA	Reference *(hypothesis)*
LCK 153390	*rs963856340*	ATTTA	*C*	*t*	TTGTA	*12*	*8*	>	*7*	10^{-6}	*A*	*more autoimmunity in RA*	↑	*Meyn, Smithgall, 2008*
	rs1446090473	TGTGT	*G*	*a*	AATTA	*12*	*9*	>	*6*	10^{-6}	*A*		↑	
	rs1039562514	GGGAC	*G*	*a**	TGGGC	*68*	*54*	>	*4*	10^{-3}	*B*		↑	
	rs1339270073	AGGGT	*G*	*a*	GGACG	*68*	*50*	>	*6*	10^{-6}	*A*		↑	
	rs1386407493	AGGCA	*G*	-	GAAGT	*68*	*37*	>	*12*	10^{-6}	*A*		↑	
	rs975584636	CTCCG	*G*	*t*	AGGAG	*68*	*48*	>	*5*	10^{-6}	*A*		↑	
	rs985600504	GGGCC	*G*	*a*	TGTGT	*80*	*46*	>	*7*	10^{-6}	*A*		↑	
	rs1013537287	AGGGG	*C*	*t*	GGTTG	*80*	*41*	>	*8*	10^{-6}	*A*		↑	
	rs550743247	CAGGG	*G*	*t*	CCGTG	*80*	*55*	>	*5*	10^{-6}	*A*		↑	
	rs185050641	TAGGG	*C*	*t*	TCAGG	*80*	*54*	>	*5*	10^{-6}	*A*		↑	
	rs894710839	CTAGG	*G*	*a*	CTCAG	*80*	*40*	>	*9*	10^{-6}	*A*		↑	
	rs565698541	GGCTA	*G*	*c*	GGTCA	*115*	*87*	>	*5*	10^{-6}	*A*		↑	
	rs1259923524	TGGGG	*C*	*t*	TAGGG	*115*	*76*	>	*8*	10^{-6}	*A*		↑	
	rs1465585974	TGCCT	*G*	*a*	TGGCG	*115*	*57*	>	*12*	10^{-6}	*A*		↑	
	rs1166162543	GGAGC	*9bp*	-	GAGGC	*53*	*44*	>	*4*	10^{-3}	*B*		↑	
	rs1357276973	GCTGC	*13bp*	-	GGAGG	*53*	*44*	>	*4*	10^{-3}	*B*		↑	
	rs1381202676	TTGTG	*G*	*a*	TTGGG	*53*	*45*	>	*3*	10^{-2}	*C*		↑	
	rs910733100	TGAAT	*T*	*c**	TACTT	*12*	*21*	<	*9*	10^{-6}	*A*	*anti-RA therapy based on small molecule inhibitors LCK-targeted*	↓	
	rs145168337	GTGGC	-	*g*	GGTTT	*122*	*139*	<	*2*	*0.05*	*D*		↓	
	rs1275483696	GCCTG	*T*	*a*	GGCGG	*122*	*139*	<	*2*	*0.05*	*D*		↓	
IL25 605658	*rs1238057770*	TTTTT	*T*	*a*	AACCT	*9*	*6*	>	*8*	10^{-6}	*A*	*more bone erosion in RA*	↑	*Lu et al., 2017*
	rs1460182246	TTTTT	*G*	*a**	GTTTT	*9*	*8*	>	*2*	*0.05*	*D*		↑	
	rs926481820	TTTAA	*G*	*a*	GAGCC	*9*	*5*	>	*9*	10^{-6}	*A*		↑	
	rs980565698	TATTG	*C*	*t*	ATTTT	*9*	*4*	>	*12*	10^{-6}	*A*		↑	
	rs551973758	GTATT	*G*	*a*	CATTT	*9*	*5*	>	*9*	10^{-6}	*A*		↑	
	rs539666492: (+11 as TSS)	GTTTT	*G*	*a*	TTTTT	*9*	*8*	>	*2*	*0.05*	*D*		↑	
	rs539666492: (+63 as TSS)	GTTTT	*G*	*a*	TTTTT	*9*	*10*	<	*2*	*0.05*	*D*	*less autoimmunity in RA*	↓	*Xu et al., 2018*
CCR7 600242	*rs952969646*	TCTCC	*G*	*t*	ACAAC	*6*	*5*	>	*5*	10^{-6}	*A*	*more bone erosion in RA*	↑	*Lee et al., 2017*
	rs1036014233	TTAAA	*AG*	-	ACAAC	*6*	*10*	<	*7*	10^{-6}	*A*	*weakened RA*	↓	*Cai et al., 2016*
	rs140037728	GTGTT	*T*	*g*	TAAAG	*5*	*7*	<	*5*	10^{-6}	*A*		↓	
	rs1222686605	ACTTA	*C*	*t, g*	GTGTT	*5*	*6*	<	*4*	10^{-3}	*B*		↓	
	rs1289870901	ACTTA	-	*a*	CGTGT	*5*	*6*	<	*3*	10^{-3}	*B*		↓	
	rs1412470887	ACACT	*T*	*g*	ACGTG	*5*	*6*	<	*2*	*0.05*	*D*		↓	

Table S4. Candidate SNP markers for RA predicted here near TBP-sites in promoters of the human protein-coding immunosuppressive genes

Gene, OMIM ID	dbSNP (Sherry et al., 2001)	5′ flank	wt	mut	3′ flank	K_D, nM, prediction						*Candidate SNP markers*	RA	Reference *(hypothesis)*
						wt	mut	Δ	Z	α	ρ			
IL1R2 147811	*rs960068265*	*AGACT*	*C*	*a*	*CACCT*	*18*	*11*	*>*	*6*	10^{-6}	*A*	*less inflammation in RA*	↓	*Ocsko et al., 2018*
	rs946299576	*AGGGA*	*T*	*g*	*TCTCT*	*56*	*42*	*>*	*6*	10^{-6}	*A*		↓	
	rs72990754	*CATTA*	*A*	*g*	*CATTC*	*10*	*13*	*<*	*3*	10^{-3}	*B*	*more inflammation in RA*	↑	
	rs960678696	*TCATT*	*A*	*g*	*ACATT*	*10*	*13*	*<*	*3*	10^{-3}	*B*		↑	
IL10 124092	*rs534191384*	*CCTTC*	*G*	*a**	*CTTTG*	*17*	*13*	*>*	*4*	10^{-3}	*B*	*weakened inflammation*	↓	*Degboe et al., 2019*
	rs1406029918	*TCCTT*	*C*	*t*	*CCTTT*	*17*	*14*	*>*	*2*	*0.05*	*D*		↓	
	rs188056661	*TACAA*	*T*	*c*	*ATAAA*	*2*	*3*	*<*	*10*	10^{-6}	*A*	*IL10-KO murine model for human RA*	↑	*Chen et al., 2017*
GAS6 600441	*rs1423343525*	*AACAC*	*G*	*a*	*CCCAG*	*102*	*61*	*>*	*9*	10^{-6}	*A*	*weakened inflammation*	↓	*Degboe et al., 2019*
	rs1011648885	*CGCCC*	*G*	*a*	*AACAC*	*102*	*45*	*>*	*14*	10^{-6}	*A*		↓	
	rs1420301093	*CCGCC*	*C*	*t*	*GAACA*	*102*	*54*	*>*	*10*	10^{-6}	*A*		↓	
	rs1490493035	*GCCCG*	*A*	*g*	*ACACG*	*102*	*158*	*<*	*7*	10^{-6}	*A*	*higher risks of erosive RA*	↑	*Bassyouni et al., 2017*
PIAS1 603566	*rs373076968*	*CGGGT*	*C*	*a, t**	*GTCCC*	*161*	*123*	*>*	*5*	10^{-6}	*A*	*less inflammation in RA*	↓	*Liu, Shuai, 2008*
	rs1399720045	*GACGG*	*G*	*a*	*TCTGC*	*161*	*129*	*>*	*4*	10^{-3}	*B*		↓	
	rs899990433	*TGACG*	*G*	*a*	*GTCGT*	*161*	*105*	*>*	*8*	10^{-6}	*A*		↓	
	rs893591978	*CTGAC*	*G*	*t**	*GGTCG*	*161*	*80*	*>*	*14*	10^{-6}	*A*		↓	
	rs1018414940	*GGGGC*	*G*	*a*	*CTGAC*	*161*	*67*	*>*	*15*	10^{-6}	*A*		↓	
	rs1206677824	*GCAGA*	*31bp*	*-*	*GGGTG*	*161*	*132*	*>*	*4*	10^{-3}	*B*		↓	
	rs1477686639	*AGAGG*	*22bp*	*-*	*GGGCC*	*161*	*118*	*>*	*6*	10^{-6}	*A*		↓	
	rs937508404	*TTTAA*	*A*	*g*	*TGGAA*	*3*	*4*	*<*	*6*	10^{-6}	*A*	*more inflammation in RA*	↑	
	rs1220814602	*TATTT*	*A*	*-*	*AATGG*	*3*	*4*	*<*	*5*	10^{-6}	*A*		↑	
	rs928754420	*CATTA*	*T*	*g*	*TTAAA*	*3*	*4*	*<*	*3*	10^{-3}	*B*		↑	
	rs1044878117	*CCATT*	*A*	*t*	*TTTAA*	*3*	*5*	*<*	*8*	10^{-6}	*A*		↑	
DUSP1 604835	*rs942073926*	*ATAAA*	*C*	*t*	*GCGCT*	*3*	*2*	*>*	*3*	10^{-3}	*B*	*reduced inflammatory response*	↓	*Toh et al., 2004*
	rs528386818	*GGCCG*	*C*	*t*	*ATATA*	*3*	*1*	*>*	*13*	10^{-6}	*A*		↓	
	rs898099424	*AGGCC*	*G*	*a*	*CATAT*	*3*	*2*	*>*	*3*	10^{-2}	*C*		↓	
	rs1352234396	*GAGGC*	*C*	*t*	*GCATA*	*3*	*2*	*>*	*2*	*0.05*	*D*		↓	
	rs1012094454	*CCGCA*	*T*	*c, g*	*ATAAA*	*3*	*7*	*<*	*11*	10^{-6}	*A*	*exceed proinflammatory cytokines worsens RA*	↑	*Salojin et al., 2006*
	rs1036564176	*GCCCGC*	*A*	*g*	*TATAA*	*2.8*	*3.4*	*<*	*2*	*0.05*	*D*		↑	

Notes: hereinafter, see under Table S1. KO, kbockout. **Genes**: *BCL6*, BCL6 transcription repressor; *CD4*, CD4 molecule; *CNMD*, chondromodulin; *DUSP1*, dual specificity phosphatase 1; *EBI3*, Epstein-Barr virus induced 3; *FGF21,* fibroblast growth factor 21; *FGF22,* fibroblast growth factor 22; *FOXP3,* forkhead box protein P3 (synonyms: scurfin; immune dysregulation); *GAS6*, growth arrest specific 6; *GDF5*, growth differentiation factor 5; *IL1R2*, interleukin 1 receptor type 2; *IL2RA*, interleukin 2 receptor subunit α (synonym: *CD25*); *IL2RB*, interleukin 2 receptor subunit β; *IL4,* Interleukin 4; *IL10*, interleukin 10 (synonyms: T-cell growth inhibitory factor; cytokine synthesis inhibitory factor); *IL10RA*, interleukin 10 receptor subunit α; *IL10RB*, interleukin 10 receptor subunit β; *IRF2*, interferon regulatory factor 2; *IRF4*, interferon regulatory factor 4; *IIRF8*, interferon regulatory factor 8; *PDCD1*, programmed cell death protein 1; *TNFRSF8*, TNF receptor superfamily member 8; *PIAS1*, protein inhibitor of activated STAT 1; *SOCS3*, suppressor of cytokine signaling 3; *TGFB2*, transforming growth factor beta 2 (synonym: glioblastoma-derived T-cell suppressor factor). **Alternative TSS**s (position relatively SNP considered), *IL2RA*: #, +32, +51; &, +16. **Deletion/insertion**, *FOXP3*: A_4 = AAAA, a_3= = aaa, a_5= = aaaaa, a_6= = aaaaaa;*IL10RB*: 11bp = cccctccccac; *IRF8*: ins_1 = ggctgcacgt; ins_2 = ggtggctgca, ggtggctgcaggtggctgca; ins_3 = ggctgcaggg; ins_4 = gtggctgcag; *ins_5* = ggctgctggt, ggttgctggt; 15bp = cgcccggcccctccc; *PIAS1*: 31bp = gggggcccgcctgcggcgggccgcgggcccc; 22bp = gggcccgcctgcggcgggccgc..

Table S4. Continued

Gene, OMIM ID	dbSNP (Sherry et al., 2001)	5′ flank	wt	mut	3′ flank	K_D, nM, prediction						*Candidate SNP markers*	RA	Reference *(hypothesis)*
						wt	mut	Δ	Z	α	ρ			
TGFB2 190220	*rs546214861*	AAATA	*T*	*c*	ATAAA	*1.5*	*2.0*	<	*5*	10^{-3}	*B*	*better healing in inflammatory RA*	↓	*Um et al., 2018*
TNFRSF8 153243	*rs1022901974*	TTAGC	*C*	*t*	ATGAT	*23*	*8*	>	*12*	10^{-6}	*A*	*lesser inflammation*	↓	*Gerli et al., 2000*
	rs1433163328	TTGTA	*G*	*a*	CTAAG	*11*	*6*	>	*8*	10^{-6}	*A*		↓	
	rs1458593063	TAGCC	*A*	*g*	TGATG	*23*	*35*	<	*5*	10^{-6}	*A*	*lower risk of RA*	↓	*Oflazoglu et al., 2009*
SOCS3 604176	*rs1337440809*	ATACC	*C*	*a,t*	GCGAG	*2*	*1*	>	*2*	*0.05*	*D*	*inhibited immune signal transduction*	↓	*Chen Y et al. 2019*
	rs900950624	TATAC	*C*	*t*	CGCGA	*2*	*1*	>	*2*	*0.05*	*D*		↓	
	rs1232909562	CCGCC	*T*	*a*	ATATA	*2*	*5*	<	*14*	10^{-6}	*A*	*acute inflammatory RA*	↑	*Wong et al., 2006*
IRF2 147576	*rs1431308705*	AAGCA	*T*	*g*	AAAGA	*7*	*6*	>	*2*	*0.05*	*D*	*better homeostasis in INFα/β system*	↓	*Taki, 2002*
	rs1183853199	TCAAG	*G*	*c*	ATAAA	*7*	*6*	>	*3*	10^{-2}	*C*		↓	
	rs1247228271	GGGTT	*C*	*t*	ATAAA	*2.3*	*2.0*	>	*2*	*0.05*	*D*		↓	
	rs1327128067	CAGGA	*G*	*a*	TAGAT	*4*	*5*	<	*4*	10^{-3}	*B*	*spontaneous T cell-caused inflammations in IRF2-KO mice*	↑	
	rs1863315	ATATT	*A*	*g*	ATACT	*2*	*3*	<	*6*	10^{-6}	*A*		↑	
	rs1192481689	TAAAT	*A*	*g*	TTAAT	*2*	*4*	<	*9*	10^{-6}	*A*		↑	
	rs1248897630	AGGTT	*T*	-	AATTT	*13*	*21*	<	*9*	10^{-6}	*A*		↑	
IRF4 601900	*rs1365999558*	TCTAT	*A*	*g*	AAGTT	*3*	*10*	<	*16*	10^{-6}	*A*	*reduced RA pain, whereas IRF4 excess elevates RA risks*	↓	*Cook et al., 2018; Rodriguez-Carrio et al., 2019*
	rs1268737685	TTCTA	*T*	*c*	AAAGT	*3*	*8*	<	*13*	10^{-6}	*A*		↓	
	rs1166436496	GTTCT	*A*	*g*	TAAAG	*3*	*9*	<	*13*	10^{-6}	*A*		↓	
	rs1421742912	AGTTC	*T*	*c*	ATAAA	*3*	*8*	<	*12*	10^{-6}	*A*		↓	
FGF22 605831	*rs1056033415*	GGACG	*C*	*t*	CCCGC	*201*	*173*	>	*3*	*0.05*	*D*	*reduced IL1B level and, thus, lesser circadian pain in RA*	↓	*Olkkonen et al., 2015; Xu et al., 2017*
	rs1468375467	GAGCC	*G*	*a*	CGGAC	*201*	*180*	>	*2*	*0.05*	*D*		↓	
	rs1311849745	GCGAA	*G*	*a*	CCGCG	*201*	*133*	>	*7*	10^{-6}	*A*		↓	
	rs897054983	CGCGC	*G*	*a*	AAGGC	*201*	*94*	>	*15*	10^{-6}	*A*		↓	
	rs1359762331	CCCGC	*C*	*a*	GCGCA	*201*	*158*	>	*5*	10^{-3}	*B*		↓	
	rs555299241	CCCCG	*C*	*t*	CGCGC	*201*	*153*	>	*5*	10^{-6}	*A*		↓	
	rs1003108764	GCCCC	*G*	*a*	CCGCG	*201*	*156*	>	*5*	10^{-6}	*A*		↓	
	rs967541155	TTGGC	*C*	*t*	CGCCG	*201*	*173*	>	*3*	10^{-2}	*C*		↓	
	rs1269961485	ATTGG	*C*	*t*	CCGCC	*201*	*61*	>	*22*	10^{-6}	*A*		↓	
	rs1447264941	AGGCG	*C*	*t*	CCCAT	*201*	*165*	>	*4*	10^{-3}	*B*		↓	
	rs1466008082	CCCCC	*G*	*t**	AGGCG	*201*	*135*	>	*7*	10^{-6}	*A*		↓	
	rs1432937560	GCGCG	*A*	*g*	AGGCA	*201*	*241*	<	*3*	10^{-2}	*C*	*high circadian pain in RA*	↑	
CNMD 605147	*rs1016067318*	GTTTG	*C*	*t*	TCATC	*73*	*36*	>	*13*	10^{-6}	*A*	*suppressed autoimmune response*	↓	*Osiecka-Iwan et al., 2018*
	rs943944018	CCTGT	*G*	*a*	CTGCG	*73*	*27*	>	*15*	10^{-6}	*A*		↓	
	rs1452553366	GTGAG	*G*	*a*	CGCTG	*84*	*73*	>	*3*	10^{-2}	*C*		↓	
	rs1165448036	GGGAG	*G*	*c*	AGAGA	*84*	*69*	>	*4*	10^{-3}	*B*		↓	
	rs1425466699	GAGAG	*A*	*c*	GGTGA	*84*	*129*	<	*7*	10^{-6}	*A*	*inflammation, cartilage damage in RA*	↑	*Zhu S et al., 2019*
	rs1179086918	GGAGG	*A*	*g*	GAGAG	*84*	*103*	<	*4*	10^{-3}	*B*		↑	
GDF5 601146	*rs1401955641*	TTATA	*C*	*t*	AAGGC	*4*	*1*	>	*9*	10^{-6}	*A*	*less cartilage damage in RA*	↓	*Wu et al., 2018*
	rs1033745622	CTCAG	*C*	*t**	CTTAT	*4*	*2*	>	*4*	10^{-3}	*B*		↓	
	rs190102137	AATTA	*C*	*a**	AACTG	*7*	*5*	>	*7*	10^{-6}	*A*		↓	
	rs1440617101	ACAAT	*T*	*c*	ACAAC	*7*	*11*	<	*8*	10^{-6}	*A*	*more cartilage damage in RA*	↑	
	rs371526190	AGACT	*A*	*c, g*	CAATT	*7*	*9*	<	*3*	10^{-2}	*C*		↑	
	rs1460570560	AAACC	*A*	*g*	GACTA	*7*	*8*	<	*2*	*0.05*	*D*		↑	

Table S4. Continued

Gene, OMIM ID	dbSNP (Sherry et al., 2001)	5′ flank	wt	mut	3′ flank	K_D, nM, prediction: wt	mut	Δ	Z	α	ρ	*Candidate SNP markers*	RA	Reference *(hypothesis)*
IRF8 601565	*rs1221072958*	GCCTT	*G*	*c*	AAGGT	*48*	*37*	>	*5*	10^{-3}	*B*	*inhibited bone resorption and inflammatory osteoclastogenesis*	↓	*Zhao et al., 2009; Ivashkiv et al., 2011*
	rs761262885	GGAGG	*C*	*t*	CTTGA	*48*	*39*	>	*4*	10^{-3}	*B*		↓	
	rs1428280758	TGCAG	-	ins_1	GTTTC	*51*	*39*	>	*4*	10^{-3}	*B*		↓	
	rs1350704251	GGGCT	*G*	*a*	CTGGT	*51*	*33*	>	*8*	10^{-6}	*A*		↓	
	rs1188357392	GCACT	*C*	*t*	AGGGC	*40*	*19*	>	*14*	10^{-6}	*A*		↓	
	rs1461193558	GCTTT	*C*	*t*	AGTTT	*40*	*13*	>	*19*	10^{-6}	*A*		↓	
	rs1175222622	CGGAG	*C*	*a*	TTTCA	*40*	*32*	>	*5*	10^{-6}	*A*		↓	
	rs767221816	AATGT	*G*	*a*	CTTCT	*9*	*5*	>	*9*	10^{-6}	*A*		↓	
	rs900666057	ATTTA	*A*	*t*	TGTGC	*9*	*6*	>	*5*	10^{-6}	*A*		↓	
	rs975160223	CATGA	*A*	*t*	TTTAA	*9*	*7*	>	*3*	10^{-2}	*C*		↓	
	rs1262062609	CCATG	*A*	*t*	ATTTA	*9*	*3*	>	*16*	10^{-6}	*A*		↓	
	rs577350223	CGGCC	*A*	*t*	TGAAT	*9*	*8*	>	*2*	*0.05*	*D*		↓	
	rs202067151	TTTGT	*G*	*a*	ATTGG	*9*	*5*	>	*9*	10^{-6}	*A*		↓	
	rs1273561223	CCTAG	*C*	*t*	CCTAC	*30*	*21*	>	*7*	10^{-6}	*A*		↓	
	rs562165753	GTCTT	*C*	*a*	CCCCT	*30*	*20*	>	*7*	10^{-6}	*A*		↓	
	rs1388986138	TGTGT	*T*	*g**	TGCTT	*30*	*22*	>	*6*	10^{-6}	*A*		↓	
	rs1439483420	TGAAT	*G*	*a*	TGTTT	*30*	*10*	>	*16*	10^{-6}	*A*		↓	
	rs76221500	AGCCG	*G*	*a, c*	TGAAT	*30*	*27*	>	*2*	*0.05*	*D*		↓	
	rs8047999	CAGCC	*G*	*a, t**	GTGAA	*30*	*27*	>	*2*	*0.05*	*D*		↓	
	rs1457926458	TGAAA	*C*	*t*	AATGG	*15*	*11*	>	*6*	10^{-6}	*A*		↓	
	rs1392611308	TTTCT	*G*	*a*	CATTT	*15*	*8*	>	*10*	10^{-6}	*A*		↓	
	rs544733904	CCGGA	*G*	*a*	GCCTT	*48*	*59*	<	*4*	10^{-3}	*B*	*higher risks of bone damage and osteoclastogenesis*	↑	*Svensson et al., 2016*
	rs530918645	GCCCC	*G*	*a**	GAGGC	*48*	*60*	<	*5*	10^{-3}	*B*		↑	
	rs1396097297	AAAAG	*T*	*c*	GATTC	*7*	*8*	<	*2*	*0.05*	*D*		↑	
	rs1442244975	TGGTG	*C*	*t*	TGTAA	*6.5*	*7.5*	<	*2*	*0.05*	*D*		↑	
	rs29001489	CTGCT	-	ins_2*	GGTTT	*51*	*131*	<	*16*	10^{-6}	*A*		↑	
	rs11268025	CAGGT	-	ins_3	TTCCC	*51*	*132*	<	*17*	10^{-6}	*A*		↑	
	rs138244824	GCTGG	-	ins_4	TGGCT	*51*	*122*	<	*16*	10^{-6}	*A*		↑	
	rs1305688250	CAGGG	-	ins_5	GGCTG	*51*	*131*	<	*16*	10^{-6}	*A*		↑	
	rs1455583236	GGGAG	*G*	*t*	GGCCT	*51*	*66*	<	*5*	10^{-6}	*A*		↑	
	rs368032120	CCAGC	*A*	*t*	CAGTG	*51*	*111*	<	*13*	10^{-6}	*A*		↑	
	rs1285752687	CCTTT	*T*	*c*	TAAGG	*9*	*17*	<	*10*	10^{-6}	*A*		↑	
	rs965092772	CACGA	*C*	*a*	CCTTT	*9*	*11*	<	*2*	*0.05*	*D*		↑	
	rs1406984724	GTTTG	*C*	*a*	TTCCT	*30*	*34*	<	*2*	*0.05*	*D*		↑	
	rs1245631188	TCTGC	*A*	*g*	TTTGT	*15*	*22*	<	*7*	10^{-6}	*A*		↑	
FOXP3 300292	*rs782492645*	TTTTG	*C*	*t*	ACCCA	*6*	*5*	>	*3*	10^{-2}	*C*	*improved immuno-suppression*	↓	*Kondo et al., 2018*
	rs782684523	TCTAC	*G*	*a*	GAGCC	*13*	*11*	>	*3*	10^{-2}	*C*		↓	
	rs782017614	GTCTA	*T*	*c*	CTACA	*6*	*8*	<	*4*	10^{-3}	*B*	*more spontaneous & aggressive RA*	↑	*Nguyen et al., 1997*
	rs781797823	TCCCC	-	*c, cc*	ACCCC	*11*	*12*	<	*2*	*0.05*	*D*		↑	
	rs1307913614	CTCCC	-	*t*	CACCC	*11*	*12*	<	*2*	*0.05*	*D*		↑	
	rs1201876295	AAAAA	A_4	$-, a_{3,5,6}$	CTATG	*11*	*14*	<	*5*	10^{-3}	*B*		↑	
IL4 147780	*rs769845954*	TAAAC	*T*	*g*	AATTG	*5*	*10*	<	*9*	10^{-6}	*A*	*higher risks of RA (cohort-based estimate), while recombinant fusion F8-IL4 is anti-RA drug*	↑	*Park et al., 2017; Hemmerle et al., 2014*
	rs746071116	GATAA	*A*	*g*	CTAAT	*5*	*11*	<	*10*	10^{-6}	*A*		↑	

Table S4. Continued

Gene, OMIM ID	dbSNP (Sherry et al., 2001)	5′ flank	wt	mut	3′ flank	K_D, nM, prediction						*Candidate SNP markers*	RA	Reference *(hypothesis)*
						wt	mut	Δ	Z	α	ρ			
BCL6 109565	*rs1462308346*	AATTT	*G*	*a*	TTTGT	*16*	*8*	>	*13*	10^{-6}	*A*	*lesser humoral autoimmunity*	↓	*Fu et al., 2018*
	rs1413689557	CTGTA	*G*	*c*	GTTTT	*14*	*9*	>	*6*	10^{-6}	*A*		↓	
	rs768115242	CTCTT	*CTC*	-	TGTAG	*14*	*7*	>	*10*	10^{-6}	*A*		↓	
	rs1007314245	CAGAG	*G*	*a*	AGGAG	*31*	*25*	>	*3*	10^{-2}	*C*		↓	
	rs761301897	CCGAC	*G*	*t**	TCACA	*31*	*26*	>	*2*	*0.05*	*D*		↓	
	rs546372632	ACAGC	*G*	*t**	AAGGC	*31*	*20*	>	*6*	10^{-6}	*A*		↓	
	rs901659721	CTACA	*G*	*c*	CGAAG	*31*	*25*	>	*3*	10^{-2}	*C*		↓	
	rs933699827	TGCTA	*C*	*t*	AGCGA	*31*	*11*	>	*12*	10^{-6}	*A*		↓	
	rs183436953	TTTCA	*A*	*g*	TCCGC	*16*	*18*	<	*3*	10^{-2}	*C*	*higher humoral autoimmunity*	↑	
	rs887672681	GACTA	*T*	*c*	AGTGG	*7*	*9*	<	*3*	10^{-2}	*C*		↑	
	rs569607079	AGACT	*A*	*g*	TAGTG	*7*	*9*	<	*3*	10^{-2}	*C*		↑	
	rs1161111705	GGTGA	*A*	*g*	TCCCC	*59*	*72*	<	*4*	10^{-3}	*B*		↑	
	rs113643275	TCCCT	*G*	*t*	GGTGA	*59*	*68*	<	*2*	*0.05*	*D*		↑	
	rs6762219	CTCCC	*T*	*c*	GGGTG	*59*	*70*	<	*3*	10^{-2}	*C*		↑	
	rs1176692677	GTAGG	*T*	*a*	TTTGA	*14*	*16*	<	*2*	*0.05*	*D*		↑	
	rs997371735	GCTAC	*A*	*c*	GCGAA	*31*	*38*	<	*3*	10^{-2}	*C*		↑	
IL10RA 146933	*rs910691859*	GCGGG	*G*	*a*	CGTCT	*23*	*19*	>	*2*	*0.05*	*D*	*lower risk of autoimmune diseases*	↓	*Qi et al., 2005*
	rs1368571579	GGGCG	*T*	*g*	CTGTA	*23*	*56*	<	*12*	10^{-6}	*A*	*higher risks of autoimmune diseases*	↑	
IL10RB 123889	*rs1019656096*	CCACC	*C*	*t**	CGCCC	*236*	*157*	>	*8*	10^{-6}	*A*	*lower risk of autoimmune diseases*	↓	*Qi et al., 2005*
	rs1312276411	CCTCC	*C*	*t*	CACCC	*236*	*142*	>	*10*	10^{-6}	*A*		↓	
	rs1261356952	AGCCC	*G*	*t*	GTCGC	*236*	*193*	>	*4*	10^{-3}	*B*		↓	
	rs1447764229	GAGCC	*C*	*a*	GGTCG	*236*	*193*	>	*4*	10^{-3}	*B*		↓	
	rs1042430120	GCGGG	*G*	*a*	TGGGA	*236*	*131*	>	*12*	10^{-6}	*A*		↓	
	rs1028922610	GCAGC	*G*	*a*	GGGTG	*236*	*182*	>	*5*	10^{-6}	*A*		↓	
	rs1179733867	TGCAG	*C*	*t*	GGGGT	*236*	*124*	>	*13*	10^{-6}	*A*		↓	
	rs1490793885	CTGCA	*G*	*a, t*	CGGGG	*236*	*97*	>	*17*	10^{-6}	*A*		↓	
	rs1011204998	CGGGG	*C*	*t*	CTGCA	*236*	*161*	>	*7*	10^{-6}	*A*		↓	
	rs1423365281	TAACA	*T*	*a*	GCCCA	*10*	*8*	>	*3*	10^{-2}	*C*		↓	
	rs780042313:a	AGAAT	*G*	*a*	TAACA	*10*	*3*	>	*16*	10^{-6}	*A*		↓	
	rs780042313:c	AGAAT	*G*	*c*	TAACA	*10*	*17*	<	*8*	10^{-6}	*A*	*higher risks of autoimmune diseases*	↑	
	rs1293774639	CCCGG	-	*11bp*	CCCCT	*236*	*263*	<	*2*	*0.05*	*D*		↑	
	rs1204272904	CCGGT	-	*15bp*	CGCCC	*236*	*285*	<	*3*	10^{-3}	*B*		↑	
	rs949254279	GCCTG	*C*	*g*	AGCGG	*236*	*288*	<	*4*	10^{-3}	*B*		↑	
	rs751406960	AATGT	*A*	*c*	ACATG	*10*	*34*	<	*19*	10^{-6}	*A*		↑	
FGF21 609436	*rs191407470*	AGCAT	*C*	*t*	TGAGC	*37*	*29*	>	*4*	10^{-3}	*B*	*suppressed NFkB-passway relieves RA*	↓	*Yu et al., 2015*
	rs2231858	AACAC	*C*	*t*	AGGAT	*37*	*32*	>	*3*	*0.05*	*D*		↓	
	rs1406076547	GCATC	*T*	*g*	GAGCA	*37*	*61*	<	*9*	10^{-6}	*A*	*high autoimmunity in RA*	↑	
EBI3 605816	*rs1031035148*	CCCCT	*C*	*a*	TCAAG	*38*	*16*	>	*15*	10^{-6}	*A*	*suppressed RA*	↓	*Nakano et al., 2015*
	rs995074666	CACCT	*C*	*a*	CCCTC	*38*	*31*	>	*4*	10^{-3}	*B*		↓	
	rs768729038	AAGTC	*T*	*g*	CCCAC	*38*	*44*	<	*3*	10^{-2}	*C*	*higher risks of RA development*	↑	*Iranshahi et al., 2019*
	rs1176851319	CTCCC	*CT*	-	CTCAA	*38*	*53*	<	*7*	10^{-6}	*A*		↑	
	rs1172235466	CTCAC	*C*	*t*	TCCCC	*38*	*43*	<	*2*	*0.05*	*D*		↑	

Table S4. Continued

Gene, OMIM ID	dbSNP (Sherry et al., 2001)	5′ flank	wt	mut	3′ flank	K_D, nM, prediction						*Candidate SNP markers*	RA	Reference *(hypothesis)*
						wt	mut	Δ	Z	α	ρ			
IL2RA 147730	*rs968128754*	*TTAAA*	*G*	*t*	*AGAGC*	*5*	*4*	>	*2*	*0.05*	*D*	*relieved RA*	↓	*Kikuchi et al., 2015*
	rs1283035521	*GCCCA*	*A*	*g*	*TACTT*	*5*	*4*	>	*3*	10^{-2}	*C*		↓	
	rs1006818127	*AATAG*	*G*	*a, t*	*CTTCT*	*11*	*9*	>	*3*	10^{-2}	*C*		↓	
	rs1360807309:#	*AGCCC*	*A*	*g*	*ATACT*	*5*	*4*	>	*2*	*0.05*	*D*		↓	
	rs1360807309:&	*AGCCC*	*A*	*g*	*ATACT*	*12*	*14*	<	*2*	*0.05*	*D*	*higher risk of RA*	↑	*Morita et al., 2016*
	rs12722687	*CTAAT*	*A*	*g*	*GGCTT*	*11*	*28*	<	*18*	10^{-6}	*A*		↑	
	rs781567058	*CCTAA*	*T*	*c*	*AGGCT*	*11*	*28*	<	*17*	10^{-6}	*A*		↑	
	rs745608471	*CTTGA*	*G*	*a*	*GGCCT*	*11*	*13*	<	*3*	10^{-3}	*B*		↑	
IL2RB 146710	*rs937506747*	*GTATA*	*A*	*-*	*GGGAC*	*4*	*6*	<	*4*	10^{-3}	*B*	*susceptible to anti-RA therapy, while IL2RB-excess elevates amount of immune memory cells and, thus, risks of RA*	↓	*Sekiguchi et al., 2008; Kurzweil et al., 2014*
	rs956497172	*GTTGT*	*A*	*g*	*TAAGG*	*4*	*13*	<	*16*	10^{-6}	*A*		↓	
	rs867567292	*GGTTG*	*T*	*c*	*ATAAG*	*4*	*16*	<	*20*	10^{-6}	*A*		↓	
PDCD1 600244	*rs1310126277*	*CCCTT*	*C*	*t*	*AACCT*	*35*	*9*	>	*26*	10^{-6}	*A*	*improved immune tolerance*	↓	*Raptopoulou et al., 2010*
	rs1291136724	*ACCTG*	*C*	*a*	*AGCTT*	*55*	*50*	>	*2*	*0.05*	*D*		↓	
	rs1203794937	*ACAAC*	*G*	*a*	*CCACC*	*55*	*41*	>	*5*	10^{-6}	*A*		↓	
	rs1314754290	*GGGGA*	*C*	*t*	*AACGC*	*55*	*25*	>	*16*	10^{-6}	*A*		↓	
	rs1218751742	*AAGGG*	*G*	*t*	*ACAAC*	*55*	*17*	>	*19*	10^{-6}	*A*		↓	
	rs368829632	*TGCTC*	*G*	*a**	*TGGTG*	*55*	*40*	>	*6*	10^{-6}	*A*		↓	
	rs777033982	*CTGCT*	*C*	*t*	*GTGGT*	*55*	*39*	>	*6*	10^{-6}	*A*		↓	
	rs1376052149	*GCACC*	*C*	*a*	*TCCCT*	*35*	*39*	<	*2*	*0.05*	*D*	*severe RA*	↑	*Yang et al., 2016*
	rs1206145937	*GGGCA*	*C*	*t*	*CCTCC*	*35*	*39*	<	*2*	*0.05*	*D*		↑	
	rs141718335	*GACAA*	*C*	*t*	*GCCAC*	*55*	*62*	<	*2*	*0.05*	*D*		↑	
CD4 186940	*rs1314027038*	*GCCTT*	*G*	*a**	*CCATC*	*57*	*46*	>	*3*	10^{-2}	*C*	*CD4 excess improves self-tolerance as well as CD4-deficiency reduces susceptibility to RA*	↓	*Haque et al., 2014; Ehinger et al., 2001*
	rs71445125	*GTGTG*	*G*	*a, c*	*AGGAG*	*57*	*25*	>	*15*	10^{-6}	*A*		↓	
	rs1027099889	*GCCTA*	*G*	*a*	*GGTGT*	*57*	*39*	>	*7*	10^{-6}	*A*		↓	

Table S5. Effects of deficit or excess in an expression of the human genes under this study on the rheumatoid arthritis (RA) according to our estimates made in this work (Tables S1 – S4)

#	Human Gene	Deficit (<)			Excess (<)		
		N_{SNP}	Effect on RA (Reference)	RA	N_{SNP}	Effect on RA (Reference)	RA
1	*ACKR1*	1	leukemia predictor from RA (Jones et al., 2006)	↑	1	more immune response in RA (Smith et al., 2008)	↑
2	*ADH7*	4	higher risks of RA (Hemminki et al., 2012)	↑		higher risks of RA (Jelski et al., 2008)	↑
3	*APOA1*	4	increased risks of cardiovascular events worsening RA (Kokkonen et al., 2017)	↑		increased risks of obesity worsening RA (Mendez-Lara et al., 2019)	↑
4	*ATF3*	7	olive oil relieves RA because of it contains natural ATF3-inhibitor (Rosillo et al., 2014)	↓	10	increased risks of RA-related lung diseases (Wu et al., 2019)	↑
5	*BCL6*	8	higher humoral autoimmunity (Fu et al., 2018)	↑	8	lesser humoral autoimmunity (Fu et al., 2018)	↓
6	*CCR6*	2	relieved adaptive immunity-driven RA (Bonelli et al., 2018)	↓		increased risks of RA (Jatczak-Pawlik et al., 2020)	↑
7	*CCR7*	5	weakened RA (Cai et al., 2016)	↓	1	more bone erosion in RA (Lee et al., 2017)	↑
8	*CD4*		reduced susceptibility to RA (Ehinger et al., 2001)	↓	3	improved self-tolerance reduces risks of RA (Haque et al., 2014)	↓
9	*CETP*	1	higher CETP-deficient mortality in RA (Ferraz-Amaro et al., 2013)	↑	5	higher risks of RA (Kim et al., 2016)	↑
10	*CNMD*	2	increased risks of inflammation, cartilage damage in RA (Zhu S et al., 2019)	↑	4	suppressed autoimmune response (Osiecka-Iwan et al., 2018)	↓
11	*COMT*	3	higher chronic pain sensitivity in RA (Finan, Zautra, 2013)	↑	14	lesser chronic pain sensitivity in RA (Finan, Zautra, 2013)	↓
12	*CTLA4*	4	higher autoimmune responses in RA (Alissafi et al., 2017)	↑	4	overlapping autoimmunity in RA (AlFadhli, 2013)	↑
13	*DHFR*	5	methotrexate susceptibility in RA (Bennike et al., 2017)	↓	2	methotrexate resistance in RA (Bennike et al., 2017)	↑
14	*DUSP1*	2	exceed proinflammatory cytokines worsens RA (Salojin et al., 2006)	↑	4	reduced inflammatory response in RA (Toh et al., 2004)	↓
15	*EBI3*	3	higher risks of RA (Iranshahi et al., 2019)	↑	2	suppressed RA (Nakano et al., 2015)	↓
16	*ESR2*	2	reduced ESR2-dependent suppression of inflammation (Armstrong et al., 2013)	↑		increases ESR2-dependent suppression of inflammation (Armstrong et al., 2013)	↓
17	*F3*	2	less migration of fibroblast-like synoviocyte (Lee et al., 2018)	↓	3	higher risks of RA (Lyberg et al., 1982)	↑
18	*F7*	3	higher risks of hemorrhagic forms of RA (Thornorsteinsson et al., 2004)	↑	8	recombinant F7 is a drug against hemophilia comorbid to RA (Drobiecki et al., 2013)	↓
19	*FGF21*	1	higher autoimmunity in RA (Yu et al., 2015)	↑	2	suppressed NFkB-passway relieves RA (Yu et al., 2015)	↓
20	*FGF22*	1	increased IL1B level rises circadian pain in RA (Olkkonen et al., 2015; Xu et al., 2017)	↑	11	reduced IL1B level relieves circadian pain in RA (Olkkonen et al., 2015; Xu et al., 2017)	↓
21	*FGFR2*	4	reduced neovascularization in RA (Brown et al., 1996)	↓	7	elevated neovascularization in RA (Brown et al., 1996)	↑

Notes: hereinafter, see under Tables S1 – S4. **N_{SNP}**, as the number of candidate SNP markers that significantly decrease or increase the affinity of the TATA-binding protein (TBP) for the promoters of the considered gene according to estimates made in this work (Tables S1 – S4) and, thereby, decrease (<) or increase (>) the expression of this gene, as has been repeatedly proven by many independent experiments (e.g., (Mogno et al., 2010)).

Table S5. Continued

#	Human Gene	Deficit (<)			Excess (<)		
		N_{SNP}	Effect on RA (Reference)	RA	N_{SNP}	Effect on RA (Reference)	RA
22	*FOXP3*	4	more spontaneous & aggressive RA (Nguyen et al., 1997)	↑	2	improved immunosuppression (Kondo et al., 2018)	↓
23	*GAS6*	1	higher risks of erosive RA (Bassyouni et al., 2017)	↑	3	weakened inflammation (Degboe et al., 2019)	↓
24	*GDF5*	3	more cartilage damage in RA (Wu et al., 2018)	↑	3	less cartilage damage in RA (Wu et al., 2018)	↓
25	*HBB*	8	thalassemia-related osteoporosis worsens RA (Giakoumi et al., 2005)	↑	3	hemolytically extracellular hemoglobin releases thrombogenic heme, which adds RA-related thrombogenesis (Bisoendial et al., 2010; Gall et al., 2018)	↑
26	*HBD*	3		↑	1		↑
27	*HLA-A*	5	reduced autoimmunity in RA (Rutten et al., 2014)	↓	5	reduced natural killer (NK) cell activity in RA (Zhang et al., 2007)	↓
28	*HSD17B1*	3	more breast cancer risk dystonically lows RA risks (Chen H et al., 2019; He et al., 2016)	↓	3	more breast cancer risk dystonically lows RA risks (Chen H et al., 2019; He et al., 2016)	↓
29	*HTR2C*		reduced serotonin-induced adipogenesis lows risks of the obesity-related RA (Priyadarshini et al., 2018)	↓	6	high risks of RA with obstructive sleep apnea (Jagannathan et al., 2017)	↑
30	*IL1B*	1	relieved RA (Rzepecka et al, 2015)	↓	1	circadian pain in RA (Olkkonen et al. 2015)	↑
31	*IL1R2*	2	more inflammation (Ocsko et al., 2018)	↑	2	less inflammation (Ocsko et al., 2018)	↓
32	*IL2RA*	4	increased risks of RA (Morita et al., 2016)	↑	4	relieved RA (Kikuchi et al., 2015)	↓
33	*IL2RB*	3	susceptible to anti-RA therapy (Sekiguchi et al., 2008)	↓		elevates amount of immune memory cells and, thus, risks of RA (Kurzweil et al., 2014)	↑
34	*IL3RA*	2	lower risk of RA conversion into cancer (Wang et al., 2019)	↓	3	more local inflammation of the synovial tissue in RA (Cavanagh et al., 2005)	↑
35	*IL4*	2	within cohort-based study: higher risks of RA (Park et al., 2017)	↑		recombinant fusion IL4-containing protein is an anti-RA drug (Hemmerle et al., 2014)	↓
36	*IL9R*	1	less inflammation of fibroblast-like synoviocyte (Raychaudhuri et al., 2018)	↓	1	more inflammation of fibroblast-like synoviocyte (Raychaudhuri et al., 2018)	↑
37	*IL10*	1	IL10-knockout micr model for human RA (Chen et al., 2017)	↑	2	weakened inflammation (Degboe et al., 2019)	↓
38	*IL10RA*	1	higher risks of autoimmune diseases (Qi et al., 2005)	↑	1	lower risk of autoimmune diseases (Qi et al., 2005)	↓
39	*IL10RB*	5		↑	11		↓
40	*IL23R*	6	less inflammation (Quiniou et al., 2014)	↓	6	more inflammation (Quiniou et al., 2014)	↑
41	*IL25*	1	less autoimmunity in RA (Xu et al., 2018)	↓	6	more bone erosion in RA (Lu et al., 2017)	↑
42	*INS*	1	less pain in RA (Abrahamson, 1952)	↓	4	more pain in RA (Abrahamson, 1952)	↑
43	*IRF2*	4	spontaneous T cell-caused inflammations in IRF2-knockout mice (Taki, 2002)	↑	3	better homeostasis in INFα/β system (Taki, 2002)	↓
44	*IRF4*	4	reduced RA pain (Cook et al., 2018)	↓		more risk of RA (Rodriguez-Carrio et al., 2019)	↑
45	*IRF5*	1	less inflammation (Duffau et al., 2015)	↓	17	more inflammation (Duffau et al., 2015)	↑
46	*IRF8*	14	higher risks of bone damage and osteoclastogenesis (Svensson et al., 2016)	↑	21	inhibited bone resorption and inflammatory osteoclastogenesis (Zhao et al., 2009; Ivashkiv et al., 2011)	↓

Table S5. Continued

#	Human Gene	Deficit (<)			Excess (<)		
		N_{SNP}	Effect on RA (Reference)	RA	N_{SNP}	Effect on RA (Reference)	RA
47	*LCK*	3	LCK-inhibitors are anti-RA drugs (Meyn, Smithgall, 2008)	↓	17	more autoimmunity (Meyn, Smithgall, 2008)	↑
48	*MBL2*	3	infection susceptibility in RA (Nisihara et al., 2016)	↑	1	increased risks of RA-caused cardiovascular diseases (Troelsen et al., 2010)	↑
49	*MLH1*	3	more risk of cancer development in RA *via* lowed DNA repair (Kullmann et al., 2000)	↑	5	increased risks of RA (Jeong et al., 2017)	↑
50	*MMP12*	2	reduced risks of RA (Liu et al., 2004)	↓	1	increased risks of RA (Liu et al., 2004)	↑
51	*NFKB1*	6	less bone erosion in RA (Torices et al., 2016)	↓	11	more inflammation (Cutolo et al., 2004)	↑
52	*NOS2*	1	relieved RA (i.e., RA-related chemotherapy target gene (Ohtsuka et al., 2002))	↓	1	worsened RA (i.e., RA-related chemotherapy target gene (Ohtsuka et al., 2002))	↑
53	*NPY*	4	lower risk of obesity-caused RA (Stofkova et al., 2009)	↓	4	higher risks of obesity-caused RA (Stofkova et al., 2009)	↑
54	*PADI4*	1	reduced arthritis severity (Seri et al., 2015)	↓	1	citrullinated protein excess (Seri et al., 2014)	↑
55	*PDCD1*	3	severe RA (Yang et al., 2016)	↑	7	improved immune tolerance (Raptopoulou et al., 2010)	↓
56	*PDYN*		pain hypersensitivity (Zheng et al., 2014)	↑	10	pain resistance (Zheng et al., 2014)	↓
57	*PIAS1*	4	more inflammation (Liu, Shuai, 2008)	↑	7	less inflammation (Liu, Shuai, 2008)	↓
58	*PTPN22*	2	reduced risks of mannan-induced RA (Sood et al., 2016)	↓	3	reduced risks of citrullination-induced RA (Chang et al., 2016)	↓
59	*RET*	3	complicated diagnosis of RA (Townsend et al., 1994; Bridgewater et al., 2008; Sarin et al., 2014)	↑	2	complicated diagnosis of RA (Townsend et al., 1994; Bridgewater et al., 2008; Sarin et al., 2014)	↑
60	*SOCS3*	1	acute inflammatory RA (Wong et al., 2006)	↑	2	inhibited immune signal transduction (Chen Y et al. 2019)	↓
61	*SOD1*	4	increased risks of RA (Staron et al., 2012)	↑		in oriental medicine, bee venom apitoxin is a drug in both RA and SOD1-deficient amyotrophic lateral sclerosis (Yang, Choi , 2013; Hemshekhar et al., 2017)	↓
62	*STAT4*	2	less severe RA (Hildner et al., 2007)	↓	5	more inflammation (Walker et al., 2006)	↑
63	*TGFB2*	1	better healing in inflammatory RA (Um et al., 2018)	↓		inhibited bone repair in RA (Um et al., 2018)	↑
64	*TGFBR2*	2	disrupted Treg-homeostasis (Wang et al., 2018)	↑	1	more proliferation of synovial fibroblast (Bira et al., 2005)	↑
65	*TNFRSF8*	1	lower risk of RA (Oflazoglu et al., 2009)	↓	2	lesser inflammation (Gerli et al., 2000)	↓
66	*TPI1*	3	TPI1-deficient neuromuscular diseases mimicks RA that hinderes RA early diagnosis in child (Richardson et al., 1984)	↑		higher risk of myocardial infarction comorbid to RA (Xu et al., 2019; Skielta et al., 2020)	↑
67	*TRAF1*		more risk of RA (Abdul-Sater et al., 2017)	↑	5	more inflammation (Shu et al., 2019)	↑
68	*ZBTB38*	18	higher chance of apoptosis, which can relieve RA (Oikawa et al., 2008)	↓	22	aggravates autoimmune RA (Ocsko et al., 2018)	↑

Supplementary Material

References

Abbas, A., Lechevrel, M., and Sichel, F. (2006) Identification of new single nucleotid polymorphisms (SNP) in alcohol dehydrogenase class IV ADH7 gene within a French population. *Arch Toxicol*. **80**, 201-205. doi: 10.1007/s00204-005-0031-7

Abrahamson, E.M. (1952) Hyperinsulinism--a factor in rheumatoid arthritis. *Am J Dig Dis*. **19**, 1-4. doi:10.1007/bf02893190

Abdul-Sater AA, Edilova MI, Clouthier DL, Mbanwi A, Kremmer E, Watts TH. (2017) The signaling adaptor TRAF1 negatively regulates Toll-like receptor signaling and this underlies its role in rheumatic disease. *Nat Immunol*. **18**, 26-35. doi: 10.1038/ni.3618

Al-Shakfa, F., Dulucq, S., Brukner, I., Milacic, I., Ansari, M., Beaulieu, P. et al. (2009) DNA variants in region for noncoding interfering transcript of dihydrofolate reductase gene and outcome in childhood acute lymphoblastic leukemia. *Clin Cancer Res*. **15**, 6931-6938. doi: 10.1158/1078-0432.CCR-09-0641

AlFadhli, S. (2013) Overexpression and secretion of the soluble CTLA-4 splice variant in various autoimmune diseases and in cases with overlapping autoimmunity. *Genet Test Mol Biomarkers*. **17**, 336-341. doi: 10.1089/gtmb.2012.0391

Armstrong, C.M., Billimek, A.R., Allred, K.F., Sturino, J.M., Weeks, B.R., and Allred, C.D. (2013) A novel shift in estrogen receptor expression occurs as estradiol suppresses inflammation-associated colon tumor formation. *Endocr Relat Cancer*. **20**, 515-525. doi: 10.1530/erc-12-0308

Arnaud, E., Barbalat, V., Nicaud, V., Cambien, F., Evans, A., Morrison, C. et al. (2000) Polymorphisms in the 5' regulatory region of the tissue factor gene and the risk of myocardial infarction and venous thromboembolism: the ECTIM and PATHROS studies. *Arterioscler Thromb Vasc Biol*. **20**, 892-898. doi: 10.1161/01.atv.20.3.892

Bassyouni, I.H., El-Wakd, M.M., Azab, N.A., and Bassyouni, R.H. (2017) Diminished soluble levels of growth arrest specific protein 6 and tyrosine kinase receptor Axl in patients with rheumatoid arthritis. *Int J Rheum Dis*. **20**, 53-59. doi:10.1111/1756-185x.12367

Bennike, T.B., Ellingsen, T., Glerup, H., Bonderup, O.K., Carlsen, T.G., Meyer, M.K. et al. (2017) Proteome analysis of rheumatoid arthritis gut mucosa. *J Proteome Res*. **16**, 346-354. doi: 10.1021/acs.jproteome.6b00598

Bira, Y., Tani, K., Nishioka, Y., Miyata, J., Sato, K., Hayashi, A. et al. (2005) Transforming growth factor beta stimulates rheumatoid synovial fibroblasts via the type II receptor. *Mod Rheumatol*. **15**, 108-113. doi: 10.1007/s10165-004-0378-2

Bisoendial, R.J., Levi, M., Tak, P.P., and Stroes, E.S. (2010) The prothrombotic state in rheumatoid arthritis: an additive risk factor for adverse cardiovascular events. *Semin Thromb Hemost*. **36**, 452-457. doi: 10.1055/s-0030-1254054

Boldt, A.B., Culpi, L., Tsuneto, L.T., de Souza, I.R., Kun, J.F., and Petzl-Erler M.L. (2006) Diversity of the MBL2 gene in various Brazilian populations and the case of selection at the mannose-binding lectin locus. *Hum Immunol*. **67**, 722-734. doi: 10.1016/j.humimm.2006.05.009

Bonelli, M., Puchner, A., Goschl, L., Hayer, S., Niederreiter, B., Steiner, G. et al. (2018) CCR6 controls autoimmune but not innate immunity-driven experimental arthritis. *J Cell Mol Med*. **22**, 5278-5285. doi: 10.1111/jcmm.13783

Bridgewater, D., Cox, B., Cain, J., Lau, A., Athaide, V., Gill, P.S. et al. (2008) Canonical WNT/beta-catenin signaling is required for ureteric branching. *Dev Biol*. **317**, 83-94. doi: 10.1016/j.ydbio.2008.02.010

Brown, K.J., Maynes, S.F., Bezos, A., Maguire, D.J., Ford, M.D., and Parish, C.R. (1996) A novel in vitro assay for human angiogenesis. *Lab Invest*. **75**, 539-555.

Supplementary Material

Burgner, D., Rockett, K., Ackerman, H., Hull, J., Usen, S., Pinder, M., and Kwiatkowski, D.P. (2003) Haplotypic relationship between SNP and microsatellite markers at the NOS2A locus in two populations. *Genes Immun*. **4**, 506-514. doi: 10.1038/sj.gene.6364022

Cai, L., Xu, H., Zhang, H., Zhang, L., Wang, G., and Nie, H.(2016) Blockade of IL-7Rα alleviates collagen-induced arthritis via inhibiting Th1 cell differentiation and CD4+ T cell migration. *Mol Immunol.* **79**, 83-91. doi: 10.1016/j.molimm.2016.09.017

Cavanagh, L.L., Boyce, A., Smith, L., Padmanabha, J., Filgueira, L., Pietschmann, P., and Thomas, R. (2005) Rheumatoid arthritis synovium contains plasmacytoid dendritic cells. *Arthritis Res Ther*. **7**, R230-R240. doi: 10.1186/ar1467

Cervera, A., Planas, A.M., Justicia, C., Urra, X., Jensenius, J.C., Torres, F.,et al. (2010) Genetically-defined deficiency of mannose-binding lectin is associated with protection after experimental stroke in mice and outcome in human stroke. *PLoS One*. **5**, e8433. doi:10.1371/journal.pone.0008433

Chadaeva, I.V., Rasskazov, D.A., Sharypova, E.B., Drachkova, I.A., Oshchepkova, E.A., Savinkova, L.K., et al. (2019). Candidate SNP-markers of rheumatoid arthritis that can significantly alter the affinity of the TATA-binding protein for human gene promoters. Vavilov Zh Genet Selektsii. 23:1047-1058. doi 10.18699/vj19.586

Chang, H.H., Liu, G.Y., Dwivedi, N., Sun, B., Okamoto, Y., Kinslow, J.D. et al. (2016) A molecular signature of preclinical rheumatoid arthritis triggered by dysregulated PTPN22. *JCI Insight*. **1**, e90045. doi: 10.1172/jci.insight.90045

Chen, S., Chen, B., Wen, Z., Huang, Z., and Ye, L. (2017) IL-33/ST2-mediated inflammation in macrophages is directly abrogated by IL-10 during rheumatoid arthritis. *Oncotarget*. **8**, 32407-32418. doi:10.18632/oncotarget.16299

Chen, H.H., Lin, C.H., Chen, D.Y., Chao, W.C., Chen, Y.H., Hung, W.T. et al. (2019) Risk of major autoimmune diseases in female breast cancer patients: A nationwide, population-based cohort study. *PLoS One*. **14**, e0222860. doi: 10.1371/journal.pone.0222860

Chen, Y., Wang, W., Chen, Y., Tang, Q., Zhu, W., Li, D., and Liao, L.(2019) MicroRNA-19a-3p promotes rheumatoid arthritis fibroblast-like synoviocytes via targeting SOCS3. *J Cell Biochem*. doi: 10.1002/jcb.28442

Collins, F.S., and Weissman, S.M. (1984) The molecular genetics of human hemoglobin. *Prog Nucleic Acid Res Mol Biol.* **31**, 315-462. doi:10.1016/s0079-6603(08)60382-7

Cook, A.D., Lee, M.C., Saleh, R., Khiew, H.W., Christensen, A.D., Achuthan, A. et al. (2018) TNF and granulocyte macrophage-colony stimulating factor interdependence mediates inflammation via CCL17. *JCI Insight*. **3**, e99249. doi: 10.1172/jci.insight.99249

Cutolo, M., Sulli, A., Capellino, S., Villaggio, B., Montagna, P., Seriolo, B., and Straub, R.H. (2004) Sex hormones influence on the immune system: basic and clinical aspects in autoimmunity. *Lupus*. **13**, 635-638. doi: 10.1191/0961203304lu1094oa

Degboe, Y., Rauwel, B., Baron, M., Boyer, J.F., Ruyssen-Witrand, A., Constantin, A., and Davignon, J.L. (2019) Polarization of rheumatoid macrophages by TNF targeting through an IL-10/STAT3 mechanism. *Front Immunol*. **10**, 3. doi: 10.3389/fimmu.2019.00003

Drobiecki, A., Pasiarski, M., Hus, I., Sokolowska, B., and Watek, M. (2013) Acquired hemophilia in the patient suffering from rheumatoid arthritis: case report. *Blood Coagul Fibrinolysis*. **24**, 874-880. doi: 10.1097/MBC.0b013e3283646635

Supplementary Material

Duffau, P., Menn-Josephy, H., Cuda, C.M., Dominguez, S., Aprahamian, T.R., Watkins, A.A. et al. (2015) Promotion of inflammatory arthritis by interferon regulatory factor 5 in a mouse model. *Arthritis Rheumatol*. **67**, 3146-3157. doi: 10.1002/art.39321

Ehinger, M., Vestberg, M., Johansson, A.C., Johannesson, M., Svensson, A., and Holmdahl, R. (2001) Influence of CD4 or CD8 deficiency on collagen-induced arthritis. *Immunology*. **103**, 291-300. doi:10.1046/j.1365-2567.2001.01257.x

Ferraz-Amaro, I., Gonzalez-Gay, M.A., Garcia-Dopico, J.A., and Diaz-Gonzalez, F. (2013) Cholesteryl ester transfer protein in patients with rheumatoid arthritis. *J Rheumatol*. **40**, 1040-1047. doi: 10.3899/jrheum.121507

Finan, P.H., and Zautra, A.J. (2013) Rheumatoid arthritis: stress affects rheumatoid arthritis, but via what mechanisms?. *Nat Rev Rheumatol*. **9**, 569-570. doi: 10.1038/nrrheum.2013.139

Fu, W., Liu, X., Lin, X., Feng, H., Sun, L., Li, S. et al. (2018) Deficiency in T follicular regulatory cells promotes autoimmunity. *J Exp Med*. **215**, 815-825. doi: 10.1084/jem.20170901

Gall, T., Petho, D., Nagy, A., Hendrik, Z., Mehes, G., Potor, L. et al. (2018) Heme induces endoplasmic reticulum stress (HIER stress) in human aortic smooth muscle cells. *Front Physiol*. **9**, 1595. doi: 10.3389/fphys.2018.01595

Gerli, R., Pitzalis, C., Bistoni, O., Falini, B., Costantini, V., Russano, A., and Lunardi, C. 2000() CD30+ T cells in rheumatoid synovitis: mechanisms of recruitment and functional role. *J Immunol*. **164**, 4399-4407. doi: 10.4049/jimmunol.164.8.4399

Giakoumi, X., Tsironi, M., Floudas, C., Polymeropoylos, E., Papalambros, E., and Aessopos, A. (2005) Rheumatoid arthritis in thalassemia intermedia: coincidence or association?. *Isr Med Assoc J*. **7**, 667-669.

Haque, M., Fino, K., Lei, F., Xiong, X., and Song, J. (2014) Utilizing regulatory T cells against rheumatoid arthritis. *Front Oncol*. **4**, 209. doi: 10.3389/fonc.2014.00209

He, W., Gauri, M., Li, T., Wang, R., and Lin, S.X. (2016) Current knowledge of the multifunctional 17β-hydroxysteroid dehydrogenase type 1 (HSD17B1). *Gene*. **588**, 54-61. doi: 10.1016/j.gene.2016.04.031

Hemmerle, T., Doll, F., and Neri, D. (2014) Antibody-based delivery of IL4 to the neovasculature cures mice with arthritis. *Proc Natl Acad Sci U S A*. **111**, 12008-12012. doi: 10.1073/pnas.1402783111

Hemminki, K., Liu, X., Ji, J., Sundquist, J., and Sundquist, K. (2012) Effect of autoimmune diseases on mortality and survival in subsequent digestive tract cancers. *Ann Oncol*. **23**, 2179-2184. doi: 10.1093/annonc/mdr590

Hemshekhar, M., Anaparti, V., Hitchon, C., and Mookherjee, N. (2017) Buprenorphine alters inflammatory and oxidative stress molecular markers in arthritis. *Mediators Inflamm*. 2017, 2515408. doi:10.1155/2017/2515408

Hildner, K.M., Schirmacher, P., Atreya, I., Dittmayer, M., Bartsch, B., Galle, P.R. et al. (2007) Targeting of the transcription factor STAT4 by antisense phosphorothioate oligonucleotides suppresses collagen-induced arthritis. *J Immunol*. **178**, 3427-3436. doi: 10.4049/jimmunol.178.6.3427

Hobbs, M., Udhayakumar, V., Levesque, M., Booth, J., Roberts, J., Tkachuk, A. et al. (2002) A new NOS2 promoter polymorphism associated with increased nitric oxide production and protection from severe malaria in Tanzanian and Kenyan children. *Lancet*. **360**, 1468-1475. doi: 10.1016/S0140-6736(02)11474-7

Hunninghake, G., Cho, M., Tesfaigzi, Y., Soto-Quiros, M., Avila, L., Lasky-Su, J. et al. (2009) MMP12, lung function, and COPD in high-risk populations. *N Engl J Med*. **361**, 2599-2608. doi:10.1056/nejmoa0904006

Supplementary Material

Iranshahi, N., Assar, S., Amiri, S.M., Zafari, P., Fekri, A., and Taghadosi, M. (2019) Decreased gene expression of Epstein-Barr Virus-Induced Gene 3 (EBI-3) may contribute to the pathogenesis of rheumatoid arthritis. *Immunol Invest.* **48**, 367-377. doi:10.1080/08820139.2018.1549066

Ivashkiv, L.B., Zhao, B., Park-Min, K.H., and Takami, M. (2011) Feedback inhibition of osteoclastogenesis during inflammation by IL-10, M-CSF receptor shedding, and induction of IRF8. *Ann N Y Acad Sci*. **1237**, 88-94. doi:10.1111/j.1749-6632.2011.06217.x

Jagannathan, R., Seixas, A., St-Jules, D., Jagannathan, L., Rogers, A., Hu, L., et al. (2017) Systems biology genetic approach identifies serotonin pathway as a possible target for obstructive sleep apnea: results from a literature search review. *Sleep Disord*. **2017**, 6768323. doi: 10.1155/2017/6768323

Jatczak-Pawlik I, Wolinski P, KsiAZek-Winiarek D, Pietruczuk M, Glabinski A. (2020) CCR6 blockade on regulatory T cells ameliorates experimental model of multiple sclerosis. *Cent Eur J Immunol*. **45**, 256-266. doi: 10.5114/ceji.2020.101241

Jeong, H., Baek, S.Y., Kim, S.W., Eun, Y.H., Kim, I.Y., Kim, H., Lee, J. et al. (2017). Comorbidities of rheumatoid arthritis: results from the Korean National Health and Nutrition Examination Survey. *PLoS One* **12**, e0176260. doi: 10.1371/journal.pone.0176260

Jelski, W., Chrostek, L., Zalewski, B., and Szmitkowski, M. (2008) Alcohol dehydrogenase (ADH) isoenzymes and aldehyde dehydrogenase (ALDH) activity in the sera of patients with gastric cancer. *Dig Dis Sci*. **53**, 2101-2105. doi: 10.1007/s10620-007-0135-4

Jones, O.Y., Spencer, C.H., Bowyer, S.L., Dent, P.B., Gottlieb, B.S., and Rabinovich, C.E. (2006) A multicenter case-control study on predictive factors distinguishing childhood leukemia from juvenile rheumatoid arthritis. *Pediatrics.* **117**, e840-e844. doi: 10.1542/peds.2005-1515

Kavlie, A., Hiltunen, L., Rasi, V., and Prydz, H. (2003) Two novel mutations in the human coagulation factor VII promoter. *Thromb Haemost*. **90**, 194-205. doi: 10.1160/th02-09-0050

Kikuchi, J., Hashizume, M., Kaneko, Y., Yoshimoto, K., Nishina, N., and Takeuchi, T. (2015) Peripheral blood CD4(+)CD25(+)CD127(low) regulatory T cells are significantly increased by tocilizumab treatment in patients with rheumatoid arthritis: increase in regulatory T cells correlates with clinical response. *Arthritis Res Ther*. **17**, 10. doi: 10.1186/s13075-015-0526-4

Kim, J.Y., Lee, E.Y., Park, J.K., Song, Y.W., Kim, J.R., and Cho, K.H. (2016) Patients with rheumatoid arthritis show altered lipoprotein profiles with dysfunctional high-density lipoproteins that can exacerbate inflammatory and atherogenic process. *PLoS One*. **11**, e0164564. doi: 10.1371/journal.pone.0164564

Kokkonen, H., Stenlund, H., and Rantapaa-Dahlqvist, S. (2017) Cardiovascular risk factors predate the onset of symptoms of rheumatoid arthritis: a nested case-control study. *Arthritis Res Ther*. **19**, 148. doi: 10.1186/s13075-017-1351-8

Kondo, Y., Yokosawa, M., Kaneko, S., Furuyama, K., Segawa, S., Tsuboi, H. et al. (2018) Review: Transcriptional regulation of CD4+ T cell differentiation in experimentally induced arthritis and rheumatoid arthritis. *Arthritis Rheumatol*. **70**, 653-661. doi: 10.1002/art.40398

Kullmann, F., Widmann, T., Kirner, A., Justen, H.P., Wessinghage, D., Dietmaier, W. et al. (2000) Microsatellite analysis in rheumatoid arthritis synovial fibroblasts. *Ann Rheum Dis.* **59**, 386-389. doi: 10.1136/ard.59.5.386

Kurzweil, V., LaRoche, A., and Oliver, P.M. (2014) Increased peripheral IL-4 leads to an expanded virtual memory CD8+ population. *J Immunol*. **192**, 5643-5651. doi: 10.4049/jimmunol.1301755

Supplementary Material

Landrum, M.J., Lee, J.M., Riley, G.R., Jang, W., Rubinstein, W.S., Church, D.M., et al. (2014). ClinVar: public archive of relationships among sequence variation and human phenotype. *Nucleic Acids Res*. **42**, D980-D985. doi:10.1093/nar/gkt1113

Lee, J., Park, C., Kim, H.J., Lee, Y.D., Lee, Z.H., Song, Y.W., and Kim, H.H. (2017) Stimulation of osteoclast migration and bone resorption by C-C chemokine ligands 19 and 21. *Exp Mol Med*. **49**, e358. doi: 10.1038/emm.2017.100

Lee, S., Kong, J.S., You, S., Kwon, H.M., Yoo, S.A., Cho, C.S., and Kim, W.U. (2018) Transcription factor NFAT5 promotes migration and invasion of rheumatoid synoviocytes via coagulation factor III and CCL2. *J Immunol.* **201**, 359-370. doi: 10.4049/jimmunol.1701097

Liu, M., Sun, H., Wang, X., Koike, T., Mishima, H., Ikeda, K. et al. (2004) Association of increased expression of macrophage elastase (matrix metalloproteinase 12) with rheumatoid arthritis. *Arthritis Rheum*. **50**, 3112-3117. doi:10.1002/art.20567

Liu, B., and Shuai, K. (2008) Targeting the PIAS1 SUMO ligase pathway to control inflammation. *Trends Pharmacol Sci.* 29, 505-509. doi: 10.1016/j.tips.2008.07.008

Lu, J., DA, M., Feng, Y., Liu, Y., Zhang, S., and Shen, H. (2017) Elevated serum IL-25 levels in rheumatoid arthritis patients with bone erosion and interstitial lung disease. *Xi Bao Yu Fen Zi Mian Yi Xue Za Zhi (Chinese Journal of Cellular and Molecular Immunology)* **33**, 1118-1122.

Lyberg, T., Prydz, H., Baklien, K., and Hoyeraal, H.M. (1982) Effect of immune complex-containing sera from patients with rheumatic diseases on thromboplastin activity of monocytes. *Thromb Res.* **25**, 193-202. DOI: 10.1016/0049-3848(82)90238-9

Martiney, J.A., Cerami, A., and Slater, A.F. (1996) Inhibition of hemozoin formation in Plasmodium falciparum trophozoite extracts by heme analogs: possible implication in the resistance to malaria conferred by the beta-thalassemia trait. *Mol Med*. **2**, 236-246.

Matsunaga, A., Sasaki, J., Han, H., Huang, W., Kugi, M., Koga, T. et al. (1999) Compound heterozygosity for an apolipoprotein A1 gene promoter mutation and a structural nonsense mutation with apolipoprotein A1 deficiency. *Arterioscler Thromb Vasc Biol*. **19**, 348-355. doi: 10.1161/01.atv.19.2.348

Mendez-Lara, K.A., Farre, N., Santos, D., Rivas-Urbina, A., Metso, J., Sanchez-Quesada, J.L. et al. (2019) Human ApoA-I overexpression enhances macrophage-specific reverse cholesterol transport but fails to prevent inherited diabesity in mice. *Int J Mol Sci*. **20**, 655. doi: 10.3390/ijms20030655

Meyn, M.A. 3rd, and Smithgall, T.E. Small molecule inhibitors of Lck: the search for specificity within a kinase family. *Mini Rev Med Chem*. **8**, 628-637. doi: 10.2174/138955708784534454

Michon, P., Woolley, I., Wood, E.M., Kastens, W., Zimmerman, P.A., and Adams, J.H. (2001) Duffy-null promoter heterozygosity reduces DARC expression and abrogates adhesion of the *P. vivax* ligand required for blood-stage infection. *FEBS Lett.* **495**, 111-114. doi: 10.1016/s0014-5793(01)02370-5

Mogno, I., Vallania, F., Mitra, R.D., and Cohen, B.A. (2010) TATA is a modular component of synthetic promoters. *Genome Res*. **20**, 1391–1397. doi:10.1101/gr.106732.110

Morita, T., Shima, Y., Wing, J.B., Sakaguchi, S., Ogata, A., and Kumanogoh, A. (2016) The proportion of regulatory T cells in patients with rheumatoid arthritis: a meta-analysis. *PLoS One*. **11**, e0162306. doi: 10.1371/journal.pone.0162306

Supplementary Material

Nakano, S., Morimoto, S., Suzuki, S., Tsushima, H., Yamanaka, K., Sekigawa, I., and Takasaki, Y. (2015) Immunoregulatory role of IL-35 in T cells of patients with rheumatoid arthritis. *Rheumatology (Oxford)*. **54**, 1498-506. doi: 10.1093/rheumatology/keu528

Nalls, M.A., Wilson, J.G., Patterson, N.J., Tandon, A., Zmuda, J.M., Huntsman, S., et al. (2008) Admixture mapping of white cell count: genetic locus responsible for lower white blood cell count in the Health ABC and Jackson Heart studies. *Am J Hum Genet*. **82,** 81-87. doi: 10.1016/j.ajhg.2007.09.003

Nguyen, L.T., Jacobs, J., Mathis, D., and Benoist, C. (2007) Where FoxP3-dependent regulatory T cells impinge on the development of inflammatory arthritis. *Arthritis Rheum*. **56**, 509-520. doi:10.1002/art.22272

Niemann, S., Broom, W.J., and Brown, R.H. Jr. (2007) Analysis of a genetic defect in the TATA box of the SOD1 gene in a patient with familial amyotrophic lateral sclerosis. *Muscle Nerve*. **36**, 704-707. doi:10.1002/mus.20855

Nisihara, R., Skare, T., Capeletto, C.M., Moreira, L., Goeldner, I., Messias-Reason, I., and Utiyama, S.R. (2016) Mannose binding lectin deficiency and susceptibility to infections in patients with rheumatoid arthritis. *Rheumatology (Oxford)*. **55**, 951-952. doi: 10.1093/rheumatology/kev413

Ocsko, T., Toth, D.M., Hoffmann, G., Tubak, V., Glant, T.T., and Rauch, T.A. (2018) Transcription factor Zbtb38 downregulates the expression of anti-inflammatory IL1r2 in mouse model of rheumatoid arthritis. *Biochim Biophys Acta Gene Regul Mech*. **1861**, 1040-1047. doi:10.1016/j.bbagrm.2018.09.007

Oflazoglu, E., Grewal, I.S., and Gerber, H. (2009) Targeting CD30/CD30L in oncology and autoimmune and inflammatory diseases. *Adv Exp Med Biol*. **647**, 174-185. doi:10.1007/978-0-387-89520-8_12

Ohtsuka, M., Konno, F., Honda, H., Oikawa, T., Ishikawa, M., Iwase, N. et al. (2002) PPA250 [3-(2,4-difluorophenyl)-6-[2-[4-(1H-imidazol-1-ylmethyl) phenoxy]ethoxy]-2-phenylpyridine], a novel orally effective inhibitor of the dimerization of inducible nitric-oxide synthase, exhibits an anti-inflammatory effect in animal models of chronic arthritis. *J Pharmacol Exp Ther*. **303**, 52-57. doi: 10.1124/jpet.102.035857

Oikawa, Y., Matsuda, E., Nishii, T., Ishida, Y., and Kawaichi, M. (2008) Down-regulation of CIBZ, a novel substrate of caspase-3, induces apoptosis. *J Biol Chem*. **283**, 14242-14247. doi: 10.1074/jbc.M802257200

Olkkonen, J., Kouri, V.P., Hynninen, J., Konttinen, Y.T., and Mandelin, J. (2015) Differentially Expressed in Chondrocytes 2 (DEC2) Increases the Expression of IL-1β and is abundantly present in synovial membrane in rheumatoid arthritis. *PLoS One*. **10**, e0145279. doi:10.1371/journal.pone.0145279

Osiecka-Iwan, A., Hyc, A., Radomska-Lesniewska, D.M., Rymarczyk, A., Skopinski, P. (2018) Antigenic and immunogenic properties of chondrocytes. Implications for chondrocyte therapeutic transplantation and pathogenesis of inflammatory and degenerative joint diseases. *Cent Eur J Immunol*. **43**, 209-219. doi: 10.5114/ceji.2018.77392

Park, H.K., Kim, S.K., Kweon, H.Y., Lee, K.G., Arasu, M.V., and Kim, Y.O. (2017) Promoter polymorphism (-590, T/C) of interleukin 4 (IL4) gene is associated with rheumatoid arthritis: An updated meta-analysis. *Saudi J Biol Sci*. **24**, 444-449. doi:10.1016/j.sjbs.2016.01.013

Peltoketo, H., Piao, Y., Mannermaa, A., Ponder, B., Isomaa, V., Poutanen, M. et al. (1994) A point mutation in the putative TATA box, detected in nondiseased individuals and patients with hereditary breast cancer, decreases promoter activity of the 17 beta-hydroxysteroid dehydrogenase type 1 gene 2 (EDH17B2) in vitro. *Genomics*. **23**, 250-252. doi: 10.1006/geno.1994.1487

Philippou, E,, and Nikiphorou, E. (2018) Are we really what we eat? Nutrition and its role in the onset of rheumatoid arthritis. *Autoimmun Rev*. **17,** 1074-1077. doi:10.1016/j.autrev.2018.05.009

Supplementary Material

Philips, S., Richter, A., Oesterreich, S., Rae, J.M., Flockhart, D.A., Perumal, N.B., and Skaar, T.C. (2012) Functional characterization of a genetic polymorphism in the promoter of the ESR2 gene. *Horm Cancer.* **3**, 37-43. doi: 10.1007/s12672-011-0086-2

Plengpanich, W., Le Goff, W., Poolsuk, S., Julia, Z., Guerin, M., and Khovidhunkit, W. (2011) CETP deficiency due to a novel mutation in the CETP gene promoter and its effect on cholesterol efflux and selective uptake into hepatocytes. *Atherosclerosis*. **216**, 370-373. doi: 10.1016/j.atherosclerosis.2011.01.051

Priyadarshini, S., Pradhan, B., Griebel, P., and Aich, P. (2018) Cortisol regulates immune and metabolic processes in murine adipocytes and macrophages through HTR2c and HTR5a serotonin receptors. *Eur J Cell Biol.* **97**, 483-492. doi: 10.1016/j.ejcb.2018.07.004

Qi, Z.M., Wang, J., Sun, Z.R., Ma, F.M., Zhang, Q.R., Hirose, S., and Jiang, Y. (2005) Polymorphism of the mouse gene for the interleukin 10 receptor alpha chain (Il10ra) and its association with the autoimmune phenotype. *Immunogenetics*. **57**, 697-702. doi: 10.1007/s00251-005-0036-7

Quiniou, C., Dominguez-Punaro, M., Cloutier, F., Erfani, A., Ennaciri, J., Sivanesan, D. et al. (2014) Specific targeting of the IL-23 receptor, using a novel small peptide noncompetitive antagonist, decreases the inflammatory response. *Am J Physiol Regul Integr Comp Physiol*. **307**, R1216-R1230. doi: 10.1152/ajpregu.00540.2013

Raptopoulou, A.P., Bertsias, G., Makrygiannakis, D., Verginis, P., Kritikos, I., Tzardi, M. et al. (2010) The programmed death 1/programmed death ligand 1 inhibitory pathway is up-regulated in rheumatoid synovium and regulates peripheral T cell responses in human and murine arthritis. *Arthritis Rheum*. **62**, 1870-1880. doi: 10.1002/art.27500

Raychaudhuri, S.K., Abria, C., Maverakis, E.M., and Raychaudhuri, S.P. (2018) IL-9 receptor: regulatory role on FLS and pannus formation. *Cytokine* **111**, 58-62. doi: 10.1016/j.cyto.2018.08.001

Richardson, M.L., Helms, C.A., Vogler, J.B. 3rd, and Genant, H.K. (1984) Skeletal changes in neuromuscular disorders mimicking juvenile rheumatoid arthritis and hemophilia. *AJR Am J Roentgenol*. **143**, 893-897. doi: 10.2214/ajr.143.4.893

Rodriguez-Carrio, J., Lopez, P., Alperi-Lopez, M., Caminal-Montero, L., Ballina-Garcia, F.J., and Suarez, A. (2019) IRF4 and IRGs delineate clinically relevant gene expression signatures in systemic lupus erythematosus and rheumatoid arthritis. *Front Immunol*. **9**, 3085. doi:10.3389/fimmu.2018.03085

Rosillo, M., Alcaraz, M.J., Sanchez-Hidalgo, M., Fernandez-Bolanos, J.G., Alarcon-de-la-Lastra, C., Ferrandiz, M.L. (2014) Anti-inflammatory and joint protective effects of extra-virgin olive-oil polyphenol extract in experimental arthritis. *J Nutr Biochem*. **25**, 1275-1281. doi: 10.1016/j.jnutbio.2014.07.006

Rutten, M..J., Dijk, F., Savci-Heijink, C.D., Buist, M.R., Kenter, G.G., van de Vijver, M.J., and Jordanova, E.S. (2014) HLA-G expression is an independent predictor for improved survival in high grade ovarian carcinomas. *J Immunol Res*. **2014**, 274584. doi: 10.1155/2014/274584

Rzepecka, J., Pineda, M.A., Al-Riyami, L., Rodgers, D.T., Huggan, J.K., Lumb, F.E. et al. (2015) Prophylactic and therapeutic treatment with a synthetic analogue of a parasitic worm product prevents experimental arthritis and inhibits IL-1β production via NRF2-mediated counter-regulation of the inflammasome. *J Autoimmun*. **60,** 59-73. doi:10.1016/j.jaut.2015.04.005

Salojin, K.V., Owusu, I.B., Millerchip, K.A., Potter, M., Platt, K.A., and Oravecz T. (2006) Essential role of MAPK phosphatase-1 in the negative control of innate immune responses. *J Immunol*. **176**, 1899-1907. doi:10.4049/jimmunol.176.3.1899

Supplementary Material

Sarin, S., Boivin, F., Li, A., Lim, J., Svajger, B., Rosenblum, N.D., and Bridgewater, D. (2014) β-Catenin overexpression in the metanephric mesenchyme leads to renal dysplasia genesis via cell-autonomous and non-cell-autonomous mechanisms. *Am J Pathol*. **184**, 1395-1410. doi: 10.1016/j.ajpath.2014.01.018

Sekiguchi, N., Kawauchi, S., Furuya, T., Inaba, N., Matsuda ,K., Ando, S. et al. (2008) Messenger ribonucleic acid expression profile in peripheral blood cells from RA patients following treatment with an anti-TNF-alpha monoclonal antibody, infliximab. *Rheumatology (Oxford)*. **47**, 780-788. doi: 10.1093/rheumatology/ken083

Seri, Y., Shoda, H., Matsumoto, I., Sumida, T., Fujio, K., and Yamamoto, K. (2014) Peptidylarginine deiminase type4 (PADI4) role in immune system. *Nihon Rinsho Meneki Gakkai Kaishi (Japanese Journal of Clinical Immunology)*. **37**, 154-159. doi: 10.2177/jsci.37.154

Seri, Y., Shoda, H., Suzuki, A., Matsumoto, I., Sumida, T., Fujio, K., and Yamamoto, K. (2015) Peptidylarginine deiminase type 4 deficiency reduced arthritis severity in a glucose-6-phosphate isomerase-induced arthritis model. *Sci Rep*. **5**, 13041. doi: 10.1038/srep13041

Shu, J.L., Zhang, X.Z., Han, L., Zhang, F., Wu, Y.J., Tang, X.Y. et al. (2019) Paeoniflorin-6'-O-benzene sulfonate alleviates collagen-induced arthritis in mice by downregulating BAFF-TRAF2-NF-κB signaling: comparison with biological agents. *Acta Pharmacol Sin*. **40**, 801-813. doi: 10.1038/s41401-018-0169-5

Skielta, M., Soderstrom, L., Rantapaa-Dahlqvist, S., Jonsson, S.W., and Mooe T. (2020) Trends in mortality, co-morbidity and treatment after acute myocardial infarction in patients with rheumatoid arthritis 1998-2013. *Eur Heart J Acute Cardiovasc Care*. 2048872619896069. doi: 10.1177/2048872619896069

Smith, E., McGettrick, H.M., Stone, M.A., Shaw, J.S., Middleton, J., Nash, G.B. et al. (2008) Duffy antigen receptor for chemokines and CXCL5 are essential for the recruitment of neutrophils in a multicellular model of rheumatoid arthritis synovium. *Arthritis Rheum.* **58**, 1968-1973. doi: 10.1002/art.23545

Sood, S., Brownlie, R.J., Garcia, C., Cowan, G., Salmond, R.J., Sakaguchi, S., and Zamoyska, R. (2016) Loss of the protein tyrosine phosphatase PTPN22 reduces mannan-induced autoimmune arthritis in SKG mice. *J Immunol*. **197,** 429-440. doi: 10.4049/jimmunol.1502656.

Staron, A., Makosa, G., and Koter-Michalak, M. (2012) Oxidative stress in erythrocytes from patients with rheumatoid arthritis. *Rheumatol Int.* **32**, 331-334. doi:10.1007/s00296-010-1611-2

Stofkova, A., Skurlova, M., Kiss, A., Zelezna, B., Zorad, S., Jurcovicova, J.(2009) Activation of hypothalamic NPY, AgRP, MC4R, AND IL-6 mRNA levels in young Lewis rats with early-life diet-induced obesity. *Endocr Regul*. **43**, 99-106. doi: 10.4149/endo_2009_03_99

Svensson, M.N., Erlandsson, M.C., Jonsson, I.M., Andersson, K.M., and Bokarewa, M.I.(2016) Impaired signaling through the Fms-like tyrosine kinase 3 receptor increases osteoclast formation and bone damage in arthritis. *J Leukoc Biol*. **99**, 413-423. doi: 10.1189/jlb.3hi1114-572rr

Sziller, I., Babula, O., Hupuczi, P., Nagy, B., Rigo, B., Szabo, G. et al. (2007) Mannose-binding lectin (MBL) codon 54 gene polymorphism protects against development of pre-eclampsia, HELLP syndrome and pre-eclampsia-associated intrauterine growth restriction. *Mol Hum Reprod*. **13**, 281-285. doi: 10.1093/molehr/gam003

Taki, S. (2002) Type I interferons and autoimmunity: lessons from the clinic and from IRF-2-deficient mice. *Cytokine Growth Factor Rev*. **13**, 379-391. doi: 10.1016/s1359-6101(02)00023-0

Thornorsteinsson, V., Magnusson, S., Hellman-Erlingsson, S., Gutmundsdottir, B.R., and Arnason A. (2004) Congenital deficiency of coagulation factor VII in an Icelandic family. *Laeknabladid.* **90**, 385-388.

Supplementary Material

Toh, M.L., Yang, Y., Leech, M., Santos, L., and Morand, E.F. 2004 Expression of mitogen-activated protein kinase phosphatase 1, a negative regulator of the mitogen-activated protein kinases, in rheumatoid arthritis: up-regulation by interleukin-1beta and glucocorticoids. *Arthritis Rheum.* **50**, 3118-3128. doi:10.1002/art.20580

Torices, S., Julia, A., Munoz, P., Varela, I., Balsa, A., Marsal, S. et al. (2016) A functional variant of TLR10 modifies the activity of NFkB and may help predict a worse prognosis in patients with rheumatoid arthritis. *Arthritis Res Ther*. **18**, 221. doi: 10.1186/s13075-016-1113-z

Townsend, R.R., McGinnis, P.A., Tuan, W.M., and Thrasher, K. (1994) Case report: bilateral adrenal pheochromocytoma. *Am J Med Sci*. **308**, 123-125. doi: 10.1097/00000441-199408000-00013

Troelsen, L.N., Garred, P., Christiansen, B., Torp-Pedersen, C., Christensen, I.J., Narvestad, E., and Jacobsen, S. (2010) Double role of mannose-binding lectin in relation to carotid intima-media thickness in patients with rheumatoid arthritis. *Mol Immunol*. **47**, 713-718. doi: 10.1016/j.molimm.2009.10.021

Um, S., Lee, J.H., and Seo, BM. (2018) TGF-β2 downregulates osteogenesis under inflammatory conditions in dental follicle stem cells. *Int J Oral Sci*. **10**, 29. doi: 10.1038/s41368-018-0028-8

Vives-Corrons, J.L, Rubinson-Skala, H., Mateo, M., Estella, J., Feliu, E., and Dreyfus J.C. (1978) Triosephosphate isomerase deficiency with hemolytic anemia and severe neuromuscular disease: familial and biochemical studies of a case found in Spain. *Hum Genet*. **42**, 171-180.

Walker, J.G., Ahern, M.J., Coleman, M., Weedon, H., Papangelis, V., Beroukas, D. et al. (2006) Expression of Jak3, STAT1, STAT4, and STAT6 in inflammatory arthritis: unique Jak3 and STAT4 expression in dendritic cells in seropositive rheumatoid arthritis. *Ann Rheum Dis*. **65**, 149-156. doi: 10.1136/ard.2005.037929

Wang, L., Wang, C., Jia, X., and Yu, J. (2018) Circulating exosomal miR-17 inhibits the induction of regulatory t cells via suppressing TGFBR II expression in rheumatoid arthritis. *Cell Physiol Biochem*. **50**, 1754-1763. doi:10.1159/000494793

Wang, M., Wu, H., Duan, M., Yang, Y., Wang, G., Che, F. et al. (2019) SS30, a novel thioaptamer targeting CD123, inhibits the growth of acute myeloid leukemia cells. *Life Sci*. **232**, 116663. doi: 10.1016/j.lfs.2019.116663

Watanabe, M., Zingg, B.C., and Mohrenweiser H.W. (1996) Molecular analysis of a series of alleles in humans with reduced activity at the triosephosphate isomerase locus. *Am J Hum Genet*. **58**, 308-316.

Wong, P.K., Egan, P.J., Croker, B.A., O'Donnell, K., Sims, N.A., Drake, S. et al. (2006) SOCS-3 negatively regulates innate and adaptive immune mechanisms in acute IL-1-dependent inflammatory arthritis. *J Clin Invest*. **116**, 1571-1581. doi: 10.1172/JCI25660

Wu, J., Zou, M., Ping, A., Deng, Z., and Cai, L. (2018) MicroRNA-449a upregulation promotes chondrocyte extracellular matrix degradation in osteoarthritis. *Biomed Pharmacother*. 105, 940-946. doi: 10.1016/j.biopha.2018.06.074

Wu, C., Lin, H., and Zhang, X. (2019) Inhibitory effects of pirfenidone on fibroblast to myofibroblast transition in rheumatoid arthritis-associated interstitial lung disease via the downregulation of activating transcription factor 3 (ATF3). *Int Immunopharmacol*. **74**, 105700. doi:10.1016/j.intimp.2019.105700

Xu, Y.H., Yu, M., Wei, H., Yao, S., Chen, S.Y., Zhu, X.L., and Li, Y.F. (2017) Fibroblast growth factor 22 is a novel modulator of depression through interleukin-1β. *CNS Neurosci Ther*. **23**, 907-916. doi: 10.1111/cns.12760

Xu, M., Lu, H., Lee, Y.H., Wu, Y., Liu, K., Shi, Y. et al. (2018) An interleukin-25-mediated autoregulatory circuit in keratinocytes plays a pivotal role in psoriatic skin inflammation. *Immunity.* **48**, 787-798.e4. doi: 10.1016/j.immuni.2018.03.019

Xu, S., Jiang, J., Zhang, Y., Chen, T., Zhu, M., Fang, C., and Mi, Y. (2019) Discovery of potential plasma protein biomarkers for acute myocardial infarction via proteomics. *J Thorac Dis*. **11**, 3962-3972. doi: 10.21037/jtd.2019.08.100

Yang, E.J., and Choi, S.M. (2013) α-Synuclein modification in an ALS animal model. *Evid Based Complement Alternat Med*. **2013**, 259381. doi:10.1155/2013/259381

Yang, L., Qiao, G., Hassan, Y., Li, Z., Zhang, X., Kong, H., Zeng, W. et al. (2016) Program Death-1 suppresses autoimmune arthritis by inhibiting Th17 response. *Arch Immunol Ther Exp (Warsz)*. **64**, 417-423. doi: 10.1007/s00005-016-0404-z

Yu, Y., Li, S., Liu, Y., Tian, G., Yuan, Q., Bai, F. et al. (2015) Fibroblast growth factor 21 (FGF21) ameliorates collagen-induced arthritis through modulating oxidative stress and suppressing nuclear factor-kappa B pathway. *Int Immunopharmacol*. **25**, 74-82. doi: 10.1016/j.intimp.2015.01.005

Zhang, J.Q., Xia, M., Shen, Y.Q., Xu, L.H., Yang, J., Miao, F.Q., and Xie, W. (2007) Research on the mechanisms and the function of abnormal HLA class I expression in hepatocellular carcinoma cell lines. *Tissue Antigens*. **69**, 415. doi: 10.1111/j.1399-0039.2007.00836.x

Zhao, B., Takami, M., Yamada, A., Wang, X., Koga, T., Hu, X. et al. (2009) Interferon regulatory factor-8 regulates bone metabolism by suppressing osteoclastogenesis. *Nat Med*. **15**, 1066-1071. doi: 10.1038/nm.2007

Zheng, B., Hu, L., Song, X., Wu, Z., Cai, R., He, L. et al. (2014) Analgesic effect of different moxibustion durations in rheumatoid arthritis rats. *J Tradit Chin Med*. **34**, 90-95. doi: 10.1016/s0254-6272(14)60060-1

Zhu, S., Qiu, H., Bennett, S., Kuek, V., Rosen, V., Xu, H., and Xu, J. (2019) Chondromodulin-1 in health, osteoarthritis, cancer, and heart disease. *Cell Mol Life Sci*. **76**, 4493-4502. doi: 10.1007/s00018-019-03225-y
