# Supplementary material for: Disruptive Selection of Human Immunostimulatory and Immunosuppressive Genes Both Provokes and Prevents Rheumatoid Arthritis, Respectively, as a Self-Domestication Syndrome
Source: Front Genet. 2021 Jun 22;12:610774. doi: 10.3389/fgene.2021.610774 (PMC8259950; doi:10.3389/fgene.2021.610774)
Supplement: Supplementary File 3 : Supplementary Keyword Search — Supplementary Figure 1. A decision logic of the keyword search for RA-related studies in the PubMed database (Lu, 2011). [file Data_Sheet_3.PDF]

Supplementary Material

Disruptive selection of human immunostimulatory and immunosuppressive genes both provokes and prevents rheumatoid arthritis, respectively, as a self-domestication syndrome

Natalya V. Klimova, Evgeniya Oshchepkova, Irina Chadaeva, Ekaterina Sharypova, Petr Ponomarenko, Irina Drachkova, Dmitry Rasskazov, Dmitry Oshchepkov, Mikhail Ponomarenko*, Ludmila Savinkova, Nikolay Kolchanov, and Vladimir Kozlov

***Correspondence:** Mikhail Ponomarenko (pon@bionet.nsc.ru)

Supplementary Keyword Search

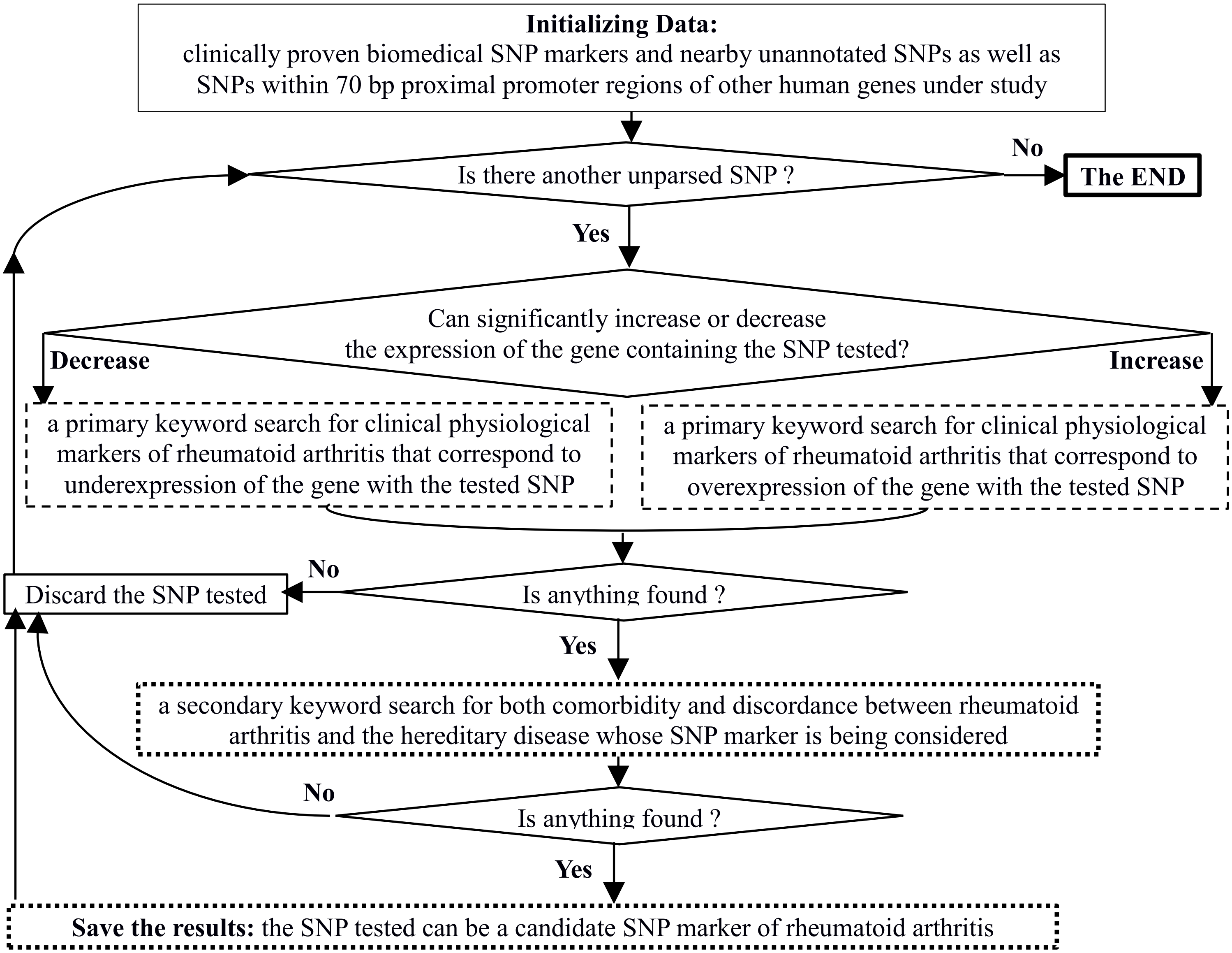

Figure S1. A decision logic of the keyword search in the PubMed database (Lu, 2011) for rheumatoid-arthritis–related studies.

References

Lu Z. (2011) PubMed and beyond: a survey of web tools for searching biomedical literature. *Database (Oxford)*. **2011**:baq036. doi: 10.1093/database/baq036
